# Supplementary material for: Self-recycling and partially conservative replication of mycobacterial methylmannose polysaccharides
Source: Commun Biol. 2023 Jan 27;6:108. doi: 10.1038/s42003-023-04448-3 (PMC9883506; doi:10.1038/s42003-023-04448-3)
Supplement: Supplementary file 2 — Supplementary Information [file 42003_2023_4448_MOESM2_ESM.pdf]

Supplementary information for:

Self-recycling and partially conservative replication of mycobacterial methylmannose polysaccharides

Ana Maranha [1, 2, 3]*, Mafalda Costa [1] *, Jorge Ripoll-Rozada [4, 5] *, José A. Manso [4, 5], Vanessa Miranda [6], Vera M. Mendes [1, 2], Bruno Manadas [1, 2], Sandra Macedo-Ribeiro [4, 5], M. Rita Ventura [6], Pedro José Barbosa Pereira [4, 5] §, Nuno Empadinhas [1, 2, 3] §

[1] CNC - Center for Neuroscience and Cell Biology, University of Coimbra, 3004-504 Coimbra, Portugal

[2] CIBB - Center for Innovative Biomedicine and Biotechnology, University of Coimbra, Coimbra, Portugal

[3] IIIUC - Institute of Interdisciplinary Research, University of Coimbra, 3030-789 Coimbra, Portugal

[4] IBMC - Instituto de Biologia Molecular e Celular, Universidade do Porto, 4200-135 Porto, Portugal

[5] Instituto de Investigação e Inovação em Saúde, Universidade do Porto, 4200-135 Porto, Portugal

[6] Bioorganic Chemistry Group, Instituto de Tecnologia Química Biológica António Xavier, Universidade Nova de Lisboa (ITQB NOVA), Av. da República, 2780-157 Oeiras, Portugal

§ Corresponding authors. Emails: numenius@cnc.uc.pt or ppereira@ibmc.up.pt

* These authors contributed equally to this work

Supplementary Methods

Chemical synthesis of 4α-oligomannosides

For the synthesis of any oligosaccharide, it is necessary to establish a glycosylation reaction between two precursors: a glycosyl donor and a glycosyl acceptor [1]. The glycosyl donor must have in its anomeric carbon a leaving group such as an halide, trichloroacetimidate, thioaryl or thioalkyl, acetate, phosphite or others, and the glycosyl acceptor must possess a free hydroxyl group so that it can react with the anomeric carbon of the donor [1]. As other sugars, mannose has several hydroxyl groups with very similar reactivity, hampering the regioselective reaction of only one of the hydroxyl groups. Thus, the synthesis of the oligomannosides posed three main challenges: 1) selective methylation of the 3-OH position; 2) differentiation of position C-4 for selective construction of the α-(1→4)-glycosidic bond in the glycosyl acceptor; and 3) differentiation of the anomeric position in the glycosyl donor for subsequent activation.

The synthesis of the unmethylated mannoside **7** (**scheme 1**) was accomplished by a process already described [2, 3, 4, 5]. The selective opening of the benzylidene acetal was performed with sodium cyanoborohydride, affording alcohol **8** (**scheme 1**), which will be used as the glycosyl acceptor in the glycosylation reaction. To obtain the glycosyl donor, the 4-OH position of alcohol **8** was protected with TBDMSOTf in dichloromethane, followed by the removal of the allyl group using $PdCl_2$ in methanol and the synthesis of mannosyl trichloroacetamidate **11** (**scheme 1**). The glycosylation reaction between donor **11** and acceptor **8**, using TMSOTf, resulted in dimannoside **12** (totally protected) and **13** (4-OH position is free) in 7% and 84% yield, respectively (**scheme 1**). Using the dimannoside **13** deprotected in the 4-OH position, it was possible to convert it to the

donor dimannoside **16** for the next glycosylation reaction through acetylation of the C-4, followed by removal of the allyl group at the anomeric position in 65% yield and the subsequent preparation of trichloroacetimidate **16** (**scheme 1**). To prepare the tetramannoside **17**, another glycosylation reaction was performed using the trichloroacetimidate **16** and acceptor **13** with a very good yield (80%), followed by the sequential deprotection reactions to obtain the final $sMan_4$ **1** (**scheme 2**). This strategy also enabled the synthesis of the trimannoside **19** through a glycosylation reaction between trichloroacetamidate **11** and acceptor **13** (**scheme 3**) and the sequential removal steps of protective groups resulted in $sMan_3$ **2** (**scheme 3**).

The strategies for synthesis of the methylated mannosides are similar to the procedures adopted for the unmethylated compound, but the selective introduction of the methyl group at C-3 was necessary, after protection of position C-1 with allyl and C-4 and C-6 positions with benzylidene acetal (**scheme 4**) [5]. In monomer **6**, the methyl group was regioselectively added, followed by the acetylation of the free 2-OH group and the selective opening of the benzylidene acetal as described above, resulting in glycosyl acceptor **23** (**scheme 4**). To obtain the glycosyl donor, sugar **23** was protected at the 4-OH position using two strategies: with TBDMSOTf and with acetic anhydride [5], affording the intermediates **24** and **27**, respectively (**scheme 4**). In both donors, the allyl group was removed and the mannosyl trichloroacetimidates **26** and **29** were synthesized (**scheme 4**). The synthesis of methylated dimannosides **30** and **31** were performed separately, using the same acceptor **23** and trichloroacetimidate **26** and **29**, respectively, in the presence of TMSOTf (**scheme 5**). For the synthesis of tetramannoside $sMetMan_4$ **3** (**scheme 5**), the methylated dimannoside **31** was converted to a glycosyl donor and the methylated tetramannoside **34** was synthesized using the tricloroacetimidate **33** and the acceptor **30** with a very good yield (86%, **scheme 6**), followed by the deprotection steps (**scheme 6**). To prepare the methylated trimannoside **36**, a glycosylation

reaction between the donor **30** and acceptor **23** was performed and the final trimannoside sMetMan$_3$ **4** was obtained after the deprotection reactions (**scheme 7**).

Allyl α-D-mannopyranoside (5)

D-mannose (20.00 g, 0.111 mol) was dissolved in distilled allyl alcohol (133.4 mL, 1.96 mol) and camphorsulfonic acid was added (133.4 mg, 0.58 mmol). The mixture was maintained under reflux, overnight. The solvent was removed under vacuum and the crude product was purified by flash column chromatography (9:1 CH_2Cl_2:MeOH, v/v) to afford compound **5** (19.4 g, 80%, α anomer) as a colorless oil.

FTIR (ATR): 3383.79 (O-H), 1647.0 (C=C), 1060.48 (C-O) cm^{-1}.

The NMR data for the α-anomer was in accordance to that described in the literature [4].

Allyl 4,6-*O*-benzylidene-α-D-mannopyranoside (6)

To a solution of **5** (18.9 g, 86 mmol) in dry THF (62.5 mL), benzaldehyde dimethyl acetal (25.8 mL, 172 mmol) and camphorsulfonic acid, in a catalytic amount, were added. The mixture was stirred and refluxed for 5 h. The mixture was quenched with a saturated aqueous $NaHCO_3$ solution (30 mL) and extracted with AcOEt (3 x 30 mL). The combined organic layers were dried with Na_2SO_4, filtered, and concentrated under vacuum. The residue was purified by recrystallization (9:1 hexane:ethyl acetate (Hex:AcOEt), v/v) to afford compound **6** (18.7 g, 75%, α anomer) as a white solid.

FTIR (ATR): 3384.58 (O-H), 1647.06 (C=C), 1094.67-1027.72 (C-O) cm^{-1}.

NMR data for the α-anomer was in accordance to that described in the literature [4].

Allyl 2,3-di-*O*-acetyl-4,6-*O*-benzylidene-α-D-mannopyranoside (7)

To a solution of **6** (6.2 g, 19.4 mmol) in dry pyridine (30 mL) at 0 °C, acetic anhydride (5.6 mL, 58.2 mmol) and a catalytic amount of DMAP were added. The mixture was stirred at 0 °C for 5 min and warmed to room temperature (rt). After 2 h, the mixture was quenched with water (30 mL) and extracted with AcOEt (3 x 30 mL). The combined organic layers were dried with Na_2SO_4, filtered, and then concentrated under vacuum. The crude product was purified by flash column chromatography (7:3 Hex:AcOEt, v/v) to afford compound **7** (6.7 g, 88%, α anomer) as a colorless oil.

NMR data for the α-anomer was in accordance to that described in the literature [4].

Allyl 2,3-di-*O*-acetyl-6-*O*-benzyl-α-D-mannopyranoside (8)

To a solution of **7** (6.69 g, 17.05 mmol) in dry THF (61 mL) at 0 °C, $NaBH_3CN$ (13.8 g, 0.2 mol) was added. The mixture was stirred at 0 °C and a solution of HCl in dry Et_2O (64 mL, 2M) was added dropwise until the reaction was completed, as judged by TLC. The solvent was removed under vacuum, re-dissolved in water (20 mL) and extracted with CH_2Cl_2 (3 x 20 mL). The combined organic layers were dried with anhydrous Na_2SO_4, filtered, and concentrated under vacuum. The crude product was purified by flash column chromatography (7:3 to 1:1 Hex:AcOEt, v/v) to afford compound **8** (5.95 g, 88%, α anomer) as a colorless oil.

FTIR (ATR): 3467.5 (O-H), 1746.9 (C=O) cm^{-1}.

^{1}H-NMR ($CDCl_3$): δ 7.39 – 7.27 (m, 5H, Ar), 6.00 - 5.77 (m, 1H, $OCH_2C\underline{H}=CH_2$), 5.39 – 5.15 (m, 4H, H-2, H-3 and $OCH_2CH=C\underline{H}_2$), 4.84 (s, 1H, H-1), 4.62 (dd, 2H, J = 24.0 Hz, J = 12.0 Hz,

$OC\underline{H}_2Ph$), 4.23 – 3.97 (m, 3H, H-4 and $OC\underline{H}_2CH{=}CH_2$), 3.91 – 3.72 (m, 3H, H-5 and H-6), 2.12 (s, 3H, $OCOC\underline{H}_3$), 2.07 (s, 3H, $OCOC\underline{H}_3$) ppm.

^{13}C-NMR ($CDCl_3$): δ 170.8 (C=O), 170.1 (C=O), 137.8 (C_q), 133.2 ($OCH_2\underline{C}H{=}CH_2$), 128.4 (Ar), 127.8 (Ar), 127.7 (Ar), 118.0 ($OCH_2CH{=}\underline{C}H_2$), 96.6 (C-1), 73.7 ($O\underline{C}H_2Ph$), 71.9 (C-3), 71.0 (C-5), 70.0 (C-6), 69.8 (C-2), 68.3 ($O\underline{C}H_2CH{=}CH_2$), 67.2 (C-4), 20.9 ($OCO\underline{C}H_3$), 20.9 ($OCO\underline{C}H_3$) ppm.

Allyl 2,3-di-*O*-acetyl-6-*O*-benzyl-4-*O*-*tert*-butyldimethylsilyl-α-D-mannopyranoside (9)

To a solution of **8** (5.81 g, 14.73 mmol) in dry dichloromethane (80 mL) at 0 °C, DIPEA (7.17 mL, 41.16 mmol) and TBDMSOTf (6.7 mL, 29.4 mmol) were added sequentially. The mixture was stirred for 20 min at 0 °C. TLC (7:3 Hex:AcOEt, v/v) analysis indicated that the reaction was completed. The mixture was washed with an aqueous solution of $NaHCO_3$ (saturated) and extracted with CH_2Cl_2. The organic layer was dried with Na_2SO_4, filtered and concentrated. Purification of the reaction crude product by flash column chromatography (eluent from 100% Hex (v/v) to 9:1 Hex:AcOEt, v/v) afforded **9** (4.13 g, 55%, α anomer) as a colorless oil.

FTIR (ATR): 1750.8 (C=O) cm^{-1}.

^{1}H-NMR ($CDCl_3$): δ 7.40 – 7.19 (m, 5H, Ar), 5.95 – 5.83 (m, 1H, $OCH_2C\underline{H}{=}CH_2$), 5.33 – 5.06 (m, 4H, H-2, H-3 and $OCH_2CH{=}C\underline{H}_2$), 4.81 (d, 1H, J = 1.5 Hz, H-1), 4.62 (t, 2H, J = 12.7 Hz, $OC\underline{H}_2Ph$), 4.20 (dd, 1H, J = 7.8 Hz, J = 5.1 Hz, 1x$OC\underline{H}_2CH{=}CH_2$), 4.05 – 3.95 (m, 2H, H-4 and 1x$OC\underline{H}_2CH{=}CH_2$), 3.84 – 3.79 (m, 1H, H-5), 3.70 (d, 2H, J = 3.4 Hz, H-6), 2.09 (s, 3H, $OCOC\underline{H}_3$), 2.00 (s, 3H, $OCOC\underline{H}_3$), 0.79 (s, 9H, $SiC(C\underline{H}_3)_3$), 0.07 (d, 6H, J = 6.7 Hz, $Si(C\underline{H}_3)_2$) ppm.

^{13}C-NMR ($CDCl_3$): δ 133.5 ($OCH_2\underline{C}H{=}CH_2$), 128.3 (Ar), 127.9 (Ar), 127.5 (Ar), 127.4 (Ar), 117. 9 ($OCH_2CH{=}\underline{C}H_2$), 96.4 (C-1), 74.9, 74. 6, 73.8, 73.3 ($O\underline{C}H_2Ph$), 72.7 (C-3), 72.5 (C-5), 69.9, 69.6, 69.1 (C-6), 68.2 ($O\underline{C}H_2CH{=}CH_2$), 66.2 (C-4), 25.7 ($SiC(\underline{C}H_3)_3$), 21.1 ($OCO\underline{C}H_3$), 20.7 ($OCO\underline{C}H_3$), -4.2 ($Si(\underline{C}H_3)_2$) ppm.

2,3-di-*O*-acetyl-6-*O*-benzyl-4-*O*-*tert*-butyldimethylsilyl-(α/β)-D-mannopyranoside (10)

To a solution of **9** (3.96 g, 7.77 mmol) in dry methanol (36 mL), $PdCl_2$ (0.275 g, 1.55 mmol) was added. The mixture was stirred at rt for 2 h and then the mixture was filtered through celite and washed with methanol. The filtrate was concentrated under vacuum and purified by flash column chromatography (7:3 to 1:1 Hex:AcOEt, v/v) to afford compound **10** (2.28 g, 63%, 14:1 (α:β)) as a colorless oil.

FTIR (ATR): 3419.1 (O-H), 1747.3 (C=O) cm^{-1}.

^{1}H-NMR ($CDCl_3$) δ 7.41 – 7.25 (m, 5H, Ar), 5.33 – 5.28 (m, 1H, H-2), 5.22 – 5.15 (m, 2H, H-1_α and H-3), 4.85 (d, 1H, H-1_β), 4.69 – 4.52 (m, 2H, $OC\underline{H}_2Ph$), 4.14 – 4.06 (m, 1H, H-5), 3.96 – 3.88 (m, 1H, H-4), 3.76 – 3.61 (m, 2H, H-6), 2.12 (s, 3H, $OCOC\underline{H}_3$), 2.03 (s, 3H, $OCOC\underline{H}_3$), 0.80 (s, 9H, $SiC(C\underline{H}_3)_3$), 0.05 – 0.01 (m, 6H, $Si(C\underline{H}_3)_2$) ppm.

^{13}C-NMR ($CDCl_3$): δ 170.2 (C=O), 137.7 (C_q), 128.47 (Ar), 128.0 (Ar), 127. 8 (Ar), 92.6 (C-1_β), 92.2 (C-1_α), 73.4 ($O\underline{C}H_2Ph$), 72.3 (C-3), 72.1 (C-5), 70.3 (C-2), 69.3 (C-6), 66.6 (C-4), 25.6 ($SiC(\underline{C}H_3)_3$), 21.0 ($OCO\underline{C}H_3$), 20.9 ($OCO\underline{C}H_3$), -4.2 ($Si(\underline{C}H_3)_2$) ppm.

2,3-di-*O*-acetyl-6-*O*-benzyl-4-*O*-*tert*-butyldimethylsilyl-α-D-mannopyranosyl trichloroacetimidate (11)

To a solution of **10** (2.28 g, 4.85 mmol) in dry CH_2Cl_2 (30 mL) at 0 °C, DBU (0.32 mL, 2.13 mmol) and trichloroacetonitrile (2.4 mL, 24.3 mmol) were added. The mixture was stirred for 10 min at 0 °C, warmed to rt and stirred for 2h. The solvent was evaporated under vacuum and the crude product was purified by flash column chromatography (7:3 Hex:AcOEt, v/v) to afford compound **11** (1.94 g, 65%) as a colorless oil.

$[\alpha]_D^{20}$ +45.4 (c 0.5, CH_2Cl_2).

FTIR (ATR): 3320.1 (N-H), 1754.5 (C=O) cm^{-1}.

^{1}H-NMR ($CDCl_3$): δ 8.69 (s, 1H, OC(N<u>H</u>)CCl_3), 7.40 – 7.22 (m, 5H, Ar), 6.26 (s, 1H, H-1), 5.50 – 5.47 (m, 1H, H-2), 5.18 (dd, 1H, *J* = 6.3 Hz, *J* = 3.2 Hz, H-3), 4.61 (s, 2H, OC<u>H</u>$_2$Ph), 4.19 (t, 1H, *J* = 9.5 Hz, H-4), 4.00 (dd, 1H, *J* = 6.6 Hz, *J* = 2.9, H-5), 3.83 – 3.69 (m, 2H, H-6), 2.14 (s, 3H, OCOC<u>H</u>$_3$), 2.03 (s, 3H, OCOC<u>H</u>$_3$), 0.83 (s, 9H, SiC(C<u>H</u>$_3$)$_3$), 0.06 (d, 6H, *J* = 3.6 Hz, Si(C<u>H</u>$_3$)$_2$) ppm.

^{13}C-NMR ($CDCl_3$): δ 170.0 (C=O), 169.8 (C=O), 160.1 (C=O), 138.3 (C_q), 128.3 (Ar), 127.5 (Ar), 127.4 (Ar), 95.1 (C-1), 75.7 (C-5), 73.3 (O<u>C</u>H$_2$Ph), 72.3 (C-3), 68.4 (C-6), 68.1 (C-2), 65.4 (C-4), 25.7 (SiC(<u>C</u>H$_3$)$_3$), 21.0 (OCO<u>C</u>H$_3$), 20.7 (OCO<u>C</u>H$_3$), -4.3 (Si(<u>C</u>H$_3$)$_2$ ppm.

Allyl 2,3-di-*O*-acetyl-6-*O*-benzyl-4-*O*-*tert*-butyldimethylsilyl-α-D-mannopyranosyl-(1→4)-2,3-di-*O*-acetyl-6-*O*-benzyl-α-D-mannopyranoside (12)

A suspension of **11** (1.88 g, 3.07 mmol), **8** (1.21 g, 3.07 mmol) and 4Å MS in CH_2Cl_2 (30 mL) was stirred at rt for 30 min. TMSOTf (0.556 mL, 3.07 mmol) was added at -20 °C and the mixture was stirred until 0 °C. After 30 min, saturated $NaHCO_3$ aqueous solution (30 mL) was added, and the mixture was extracted with CH_2Cl_2 (3 x 10 mL). The combined organic phases were dried with

anhydrous Na_2SO_4, filtered, and concentrated under vacuum. The crude product was purified by flash column chromatography (7:3 Hex:AcOEt, v/v) to afford compound **12** (0.174 g, 7%, α anomer) as a colorless oil.

FTIR (ATR): 1747.6 (C=O) cm^{-1}.

^{1}H-NMR ($CDCl_3$) δ 7.39 – 7.21 (m, 10H, Ar), 5.96 – 5.84 (m, 1H, $OCH_2C\underline{H}=CH_2$), 5.37 – 5.24 (m, 3H, H-2 B, H-3 B and 1x$OCH_2CH=C\underline{H}_2$), 5.24 – 5.17 (m, 2H, H-2 A and 1x$OCH_2CH=C\underline{H}_2$), 5.05 – 4.97 (m, 2H, H-1 A and H-3 A), 4.87 – 4.81 (m, 1H, H-1 B), 4.64 – 4.48 (m, 4H, $OC\underline{H}_2Ph$ A-B), 4.23 – 4.08 (m, 2H, H-4 B and 1x$OC\underline{H}_2CH=CH_2$), 4.07 – 3.96 (m, 2H, H-4 A and 1x$OC\underline{H}_2CH=CH_2$), 3.96 – 3.73 (m, 3H, H-5 A-B and H-6 B), 3.64 – 3.51 (m, 2H, H-6 A), 2.17 (s, 3H, $OCOC\underline{H}_3$), 2.11 – 2.03 (m, 6H, $(OCOC\underline{H}_3)_2$), 2.00 (s, 3H, $OCOC\underline{H}_3$), 0.82 (s, 9H, $SiC(C\underline{H}_3)_3$), 0.03 (s, 6H, $Si(C\underline{H}_3)_2$) ppm.

^{13}C-NMR ($CDCl_3$): δ 170.1 (C=O), 170.0 (C=O), 169.8 (C=O), 138.5 (C_q), 138.2 (C_q), 133.2 ($OCH_2\underline{C}H=CH_2$), 128.3 (Ar), 128.2 (Ar), 127.4 (Ar), 118.1 ($OCH_2CH=\underline{C}H_2$), 100.0 (C-1 A), 96.4 (C-1 B), 74.3 (C-4 B), 73.8 (C-5 A), 73.3 ($O\underline{C}H_2Ph$ A or B), 73.2 ($O\underline{C}H_2Ph$ A or B), 72.3(C-3 A), 71.5 (C-3 B), 71.04 (C-5 B), 70.0 (C-2 A), 69.8 (C-2 B), 69.1 (C-6 B), 68.9 (C-6 A), 68.4 ($O\underline{C}H_2CH=CH_2$), 65.9 (C-4 A), 25.7 ($SiC(\underline{C}H_3)_3$), 21.0 ($OCO\underline{C}H_3$), 20.9 ($OCO\underline{C}H_3$), 20.8 ($OCO\underline{C}H_3$), 20.7 ($OCO\underline{C}H_3$), -4.3 ($Si(\underline{C}H_3)_2$) ppm.

Allyl 2,3-di-*O*-acetyl-6-*O*-benzyl-α-D-mannopyranosyl-(1→4)-2,3-di-*O*-acetyl-6-*O*-benzyl-α-D-mannopyranoside (13)

A suspension of **11** (1.88 g, 3.07 mmol), **8** (1.21 g, 3.07 mmol) and 4Å MS in dry CH_2Cl_2 (30 mL) was stirred at rt for 30 min. TMSOTf (0.556 mL, 3.07 mmol) was added at -20 °C and the mixture

was stirred until 0 °C. After 30 min, saturated $NaHCO_3$ aqueous solution (30 mL) was added, and the mixture was extracted with CH_2Cl_2 (3 x 10 mL). The combined organic phases were dried with anhydrous Na_2SO_4, filtered, and concentrated under vacuum. The crude product was purified by flash column chromatography (7:3 Hex:AcOEt, v/v) to afford compound **13** (1.88 g, 84%, α anomer) as a colorless oil.

FTIR (ATR): 3479.7 (O-H), 1746.7 (C=O) cm^{-1}.

^{1}H-NMR ($CDCl_3$): δ 7.39 – 7.20 (m, 10H, Ar), 5.90 (m, 1H, $OCH_2C\underline{H}=CH_2$), 5.38 – 5.17 (m, 4H, $OCH_2CH=C\underline{H}_2$, H-2 B and H-3 B), 5.17 – 5.00 (m, 3H, H-1 A, H-2 A and H-3 A), 4.83 (d, 1H, *J* = 1.5 Hz, H-1 B), 4.55 (d, 3H, *J* = 12.5 Hz, $OC\underline{H}_2Ph$ A, 1x$OC\underline{H}_2Ph$ B), 4.44 (d, 1H, *J*=11.9 Hz, 1x$OC\underline{H}_2Ph$ B), 4.29 – 4.07 (m, 2H, H-4 B and 1x$OC\underline{H}_2CH=CH_2$), 4.07 – 3.70 (m, 6H, H-4 A, 1x$C\underline{H}_2Ph$, H-5 B, H-5 A and H-6 B), 3.68 - 3.62 (m, 1H, 1xH-6 A), 3.60 – 3.54 (m, 1H, 1xH-6 A), 2.13 – 2.03 (m, 12H, $(OCOC\underline{H}_3)_4$) ppm.

^{13}C-NMR ($CDCl_3$): δ 170.1 (C=O), 137.8 (C_q), 133.6 ($OCH_2\underline{C}H=CH_2$), 128.4 (Ar), 128.3 (Ar), 127.7 (Ar), 127.4 (Ar), 118.2 ($OCH_2CH=\underline{C}H_2$), 99.4 (C-1 A), 96.4 (C-1 B), 73.7 ($O\underline{C}H_2Ph$ B), 73.3 (C-4), 72.1 ($O\underline{C}H_2Ph$ A),72.2 (C-5 A), 71.8 (C-3 B), 71.4 (C-2 A or C-3 A), 70.9 (C-5 B), 69.9 (C-2 A or C-3 A), 69.5 (C-6 A), 69.1 (C-6 B), 68.4 ($O\underline{C}H_2CH=CH_2$), 67.1 (C-4 A), 20.9 ($OCO\underline{C}H_3$), 20.8 ($OCO\underline{C}H_3$) ppm.

Allyl 2,3,4-tri-*O*-acetyl-6-*O*-benzyl-α-D-mannopyranosyl-(1→4)-2,3-di-*O*-acetyl-6-*O*-benzyl-α-D-mannopyranoside (14)

To a solution of **13** (0.98 g, 1.34 mmol) in dry pyridine (8 mL) at 0 °C, acetic anhydride (194 µL, 2.05 mmol) and a catalytic amount of DMAP were added. The mixture was stirred at 0 °C for 5

min and warmed to rt. After 2 h, the mixture was quenched with water (5 mL) and extracted with AcOEt (3 x 10 mL). The combined organic layers were dried with anhydrous Na_2SO_4, filtered, and then concentrated under vacuum. The crude product was purified by flash column chromatography (7:3 Hex:AcOEt, v/v) to afford compound **14** (0.92 g, 89%, α anomer) as a colorless oil.

FTIR (ATR): 1747.1 (C=O) cm^{-1}.

^{1}H-NMR ($CDCl_3$): δ 7.39 – 7.22 (m, 10H, Ar), 5.96 – 5.84 (m, 1H, $OCH_2C\underline{H}=CH_2$), 5.38 – 5.05 (m, 8H, H-1 A, H-2 A-B, H-3 A-B, H-4 A and $OCH_2CH=C\underline{H}_2$), 4.84 (s, 1H, H-1 B), 4.54 (d, 3H, *J* = 13.1 Hz, $OC\underline{H}_2Ph$ A and 1x$OC\underline{H}_2Ph$ B), 4.37 (d, 1H, *J* = 11.9 Hz, 1x$OC\underline{H}_2Ph$ B), 4.23 – 4.09 (m, 2H, H-4 B and 1x$OC\underline{H}_2CH=CH_2$), 4.06 – 3.82 (m, 4H, 1x$OC\underline{H}_2CH=CH_2$, H-5 A-B and 1xH-6 B), 3.75 (d, 1H, *J* = 10.2 Hz, 1xH-6 B), 3.46 – 3.35 (m, 2H, H-6 A), 2.15 – 2.08 (m, 9H, $(OCOC\underline{H}_3)_3$), 1.98 (s, 3H, $OCOC\underline{H}_3$), 1.91 (s, 3H, $OCOC\underline{H}_3$) ppm.

^{13}C-NMR ($CDCl_3$): δ 170.0 (C=O), 169.7 (C=O), 138.4 (C_q), 133.2 ($OCH_2\underline{C}H=CH_2$), 128.3 (Ar), 128.3 (Ar), 127.9 (Ar), 127.7 (Ar), 127.4 (Ar), 118.2 ($OCH_2CH=\underline{C}H_2$), 99.4 (C-1 A), 96.4 (C-1 B), 73.9 (C-4 B), 73.5 ($O\underline{C}H_2Ph$ B), 73.3 ($O\underline{C}H_2Ph$ A), 71.6 (C-3 B), 71.0 (C-5 B), 70.6 (C-5 A), 69.9 (C-2 B), 69.8 (C-2 A), 69.1 (C-6 B), 68.9 (C-3 A), 68.7 ($O\underline{C}H_2CH=CH_2$), 68.5 (C-6 A), 66.6 (C-4 A), 20.9 ($OCO\underline{C}H_3$), 20.8 ($OCO\underline{C}H_3$), 20.7 ($OCO\underline{C}H_3$), 20.6 ($OCO\underline{C}H_3$) ppm.

2,3,4-tri-*O*-acetyl-6-*O*-benzyl-α-D-mannopyranosyl-(1→4)-2,3-di-*O*-acetyl-6-*O*-benzyl-(α/β)-D-mannopyranoside (15)

To a solution of **14** (0.92 g, 1.19 mmol) in dry methanol (17.5 mL), $PdCl_2$ (42.2 mg, 0.238 mmol) was added. The mixture was stirred at rt for 2 h and then the mixture was filtered through celite

and washed with methanol. The filtrate was concentrated under vacuum and purified by flash column chromatography (7:3 to 1:1 Hex:AcOEt, v/v) to afford compound **15** (0.778 g, 89%, 16:1 (α:β)) as a colorless oil.

FTIR (ATR): 3467.0 (O-H), 1746.9 (C=O) cm^{-1}.

^{1}H-NMR ($CDCl_3$): δ 7.38 – 7.20 (m, 10H, Ar), 5.37 (dd, 1H, *J* = 6.2 Hz, *J* = 3.3 Hz, H-3 B), 5.35 – 5.27 (m, 2H, H-2 B and H-4 A), 5.25 – 5.18 (m, 2H, H-1 B_α and H-3 A), 5.15 – 5.11 (m, 1H, H-2 A), 5.07 (d, 1H, *J* = 2.0 Hz, H-1 A), 4.93 (s, 1H, H-1 B_β) 4.59 – 4.49 (m, 3H, $OC\underline{H}_2Ph$ A and 1x$OC\underline{H}_2Ph$ B), 4.38 (d, 1H, *J* = 12.0 Hz, 1x$OC\underline{H}_2Ph$ B), 4.19 – 4.04 (m, 2H, H-4 B and H-5 B), 3.94 – 3.87 (m, 1H, H-5 A), 3.85 – 3.79 (m, 1H, 1xH-6 B), 3.78 – 3.73 (m, 1H, 1xH-6 B), 3.40 (dd, 1H, *J* = 7.9Hz, *J* = 4.7 Hz, 1xH-6 A), 3.34 (dd, 1H, *J* = 7.9 Hz, *J* = 2.8 Hz, 1xH-6 A), 2.11 (d, 6H, *J* = 6.7 Hz, $(OCOC\underline{H}_3)_2$), 2.05 (s, 3H, $OCOC\underline{H}_3$), 1.98 (s, 3H, $OCOC\underline{H}_3$), 1.92 (s, 3H, $OCOC\underline{H}_3$) ppm.

^{13}C-NMR ($CDCl_3$): δ 170.1 (C=O), 170.0 (C=O), 169.8 (C=O), 169.70 (C=O), 138.2 (C_q), 137.7 (C_q), 128.3 (Ar), 127.9 (Ar), 127.7 (Ar), 127.6 (Ar), 127.6 (Ar) 99.4 (C-1 A), 92.6 (C-1 B_β), 92.1 (C-1 B_α), 74.1 (C-4 B), 73.5 ($O\underline{C}H_2Ph$ B), 73.4 ($O\underline{C}H_2Ph$ B), 71.17 (C-3 B), 70.8 (C-5 B), 70.7 (C-5 A), 70.1 (C-2 B), 69.8 (C-2 A), 69.4 (C-6 B), 68.9 (C-3 A), 68.5 (C-6 A), 66.6 (C-4 A), 20.9 ($OCO\underline{C}H_3$), 20.8 ($OCO\underline{C}H_3$), 20.7 ($OCO\underline{C}H_3$), 20.6 ($OCO\underline{C}H_3$) ppm.

2,3,4-tri-*O*-acetyl-6-*O*-benzyl-α-D-mannopyranosyl-(1→4)-2,3-di-*O*-acetyl-6-*O*-benzyl-α-D-mannopyranosyl trichloroacetimidate (16)

To a solution of **15** (0.693 g, 0.945 mmol) in dry CH_2Cl_2 (6 mL) at 0 °C, DBU (62 μL, 0.416 mmol) and trichloroacetonitrile (0.474 mL, 4.73 mmol) were added. The mixture was stirred for

10 min at 0 °C, warmed to rt and stirred for 2 h. The solvent was evaporated under vacuum and the crude product was purified by flash column chromatography (6:4 Hex:AcOEt, v/v) to afford compound **16** (0.666 g, 80%) as a colorless oil.

$[\alpha]_D^{20}$ +76.0 (c 0.33, CH_2Cl_2).

FTIR (ATR): 3350.5 (N-H), 1748.2 (C=O) cm^{-1}.

^{1}H-NMR ($CDCl_3$): δ 8.71 (s, 1H, OC(N<u>H</u>)CCl_3), 7.36 – 7.23 (m, 10H, Ar), 6.26 (d, 1H, *J* = 1.5 Hz, H-1 B), 5.51 – 5.47 (m, 1H, H-2 B), 5.40 – 5.29 (m, 2H, H-3 B and H-4 A), 5.24 (dd, 1H, *J* = 3.1 Hz, H-3 A), 5.18 – 5.13 (m, 2H, H-2 A and H-1 A), 4.59 – 4.50 (s, 3H, OC<u>H</u>$_2$Ph A and 1xOC<u>H</u>$_2$Ph B), 4.34 (d, 1H, *J* = 11.0 Hz, 1xOC<u>H</u>$_2$Ph B), 4.39 – 4.29 (m, 2H, 1xOC<u>H</u>$_2$Ph B and H-4 B), 4.11 – 4.05 (m, 1H, H-5 B), 3.99 – 3.89 (m, 2H, H-5 A and 1xH-6 B), 3.76 (d, 1H, *J* = 11.6 Hz, 1xH-6 B), 3.45 – 3.35 (m, 2H, H-6 A), 2.15 – 2.11 (m, 6H, (OCOC<u>H</u>$_3$)$_2$), 2.06 (s, 3H, OCOC<u>H</u>$_3$), 1.98 (s, 3H, OCOC<u>H</u>$_3$), 1.90 (s, 3H, OCOC<u>H</u>$_3$) ppm.

^{13}C-NMR ($CDCl_3$): δ 170.0 (C=O), 169.9 (C=O), 169.7 (C=O), 160.1 (C_q), 138.4 (C_q), 137.7 (C_q), 128.3 (Ar), 127.9 (Ar), 127.7 (Ar), 127.5 (Ar), 99.4 (C-1 A), 94.8 (C-1 B), 74.0 (C-5 B), 73.5 (O<u>C</u>H$_2$Ph B), 73.4 (O<u>C</u>H$_2$Ph A), 72.5 (C-4 B), 71.2 (C-3 B), 70.7 (C-5 A), 69.7 (C-2 A), 69.0 (C-3 A), 68.7 (C-6 A) 68.5 (C-6 B), 68.1 (C-2 B), 66.5 (C-4 A), 20.9 (OCO<u>C</u>H$_3$), 20.8 (OCO<u>C</u>H$_3$), 20.7 (OCO<u>C</u>H$_3$), 20.6 (OCO<u>C</u>H$_3$) ppm.

Allyl 2,3,4-tri-*O*-acetyl-6-*O*-benzyl-α-D-mannopyranosyl-(1→4)-2,3-di-*O*-acetyl-6-*O*-benzyl-α-D-mannopyranosyl-(1→4)-2,3-di-*O*-acetyl-6-*O*-benzyl-α-D-mannopyranosyl-(1→4)-2,3-di-*O*-acetyl-6-*O*-benzyl-α-D-mannopyranoside (17)

A suspension of **16** (0.597 g, 0.68 mmol), **13** (0.497 g, 0.68 mmol) and 4Å MS in dry CH_2Cl_2 (15 mL) was stirred at rt for 30 min. TMSOTf (0.123 mL, 0.68 mmol) was added at -20 °C and the mixture was stirred until 0 °C. After 30 min, saturated $NaHCO_3$ aqueous solution (10 mL) was added, and the mixture was extracted with CH_2Cl_2 (3 x 10 mL). The combined organic phases were dried with anhydrous Na_2SO_4, filtered, and concentrated under vacuum. The crude product was purified by flash column chromatography (7:3 Hex:AcOEt, v/v) to afford compound **17** (0.798 g, 80%, α anomer) as a colorless oil.

FTIR (ATR): 1747.1 (C=O) cm^{-1}.

^{1}H-NMR ($CDCl_3$): δ 7.37 – 7.21 (m, 20H, Ar), 5.97 – 5.84 (m, 1H, $OCH_2C\underline{H}=CH_2$), 5.36 – 5.02 (m, 16H, H-1 A-C, H-2 A-D, H-3 A-D, H-4 A-C, $OCH_2CH=C\underline{H}_2$), 4.84 (d, 1H, *J* = 1.3 Hz, H-1 D), 4.63 – 4.30 (m, 8H, $OC\underline{H}_2Ph$ A-D), 4.23 – 3.68 (m, 12H, H-4 D, H-5 A-D, 5xH-6, $OC\underline{H}_2CH=CH_2$), 3.54 (d, 1H, *J* = 11.6 Hz, 1xH-6), 3.42 – 3.31 (m, 2H, H-6), 2.13 – 1.88 (m, 27H, $(OCOC\underline{H}_3)_9$) ppm.

^{13}C-NMR ($CDCl_3$): δ 170.0 (C=O), 169.9 (C=O), 169.8 (C=O), 169.8 (C=O), 169.7 (C=O), 138.5 (C_q) 138.4 (C_q), 138.3 (C_q), 137.7 (C_q), 133.2 ($OCH_2\underline{C}H=CH_2$), 128.4 (Ar), 128.3 (Ar), 128.2 (Ar), 127.9 (Ar), 127.7 (Ar), 127.5 (Ar), 127.4 (Ar), 118.2 ($OCH_2CH=\underline{C}H_2$), 99.4 (C-1 A or B or C), 99.2 (C-1 A or B or C), 99.1 (C-1 A or B or C), 96.4 (C-1 D), 73.9, 73.6 ($\underline{C}H_2Ph$ A or B or C or D), 73.5 ($\underline{C}H_2Ph$ A or B or C or D), 73.3 ($\underline{C}H_2Ph$ A or B or C or D), 73.2 ($\underline{C}H_2Ph$ A or B or C or D), 73.2, 73.1, 72.2, 72.0, 71.7, 71.4, 71.2, 71.1, 70.9, 70.6, 70.0, 69.9, 69.8, 68.9 (C-6), 68.6 (C-6), 68.4 (C-6), 66.7, 20.9 ($OCO\underline{C}H_3$), 20.8 ($OCO\underline{C}H_3$), 20.7 ($OCO\underline{C}H_3$), 20.6 ($OCO\underline{C}H_3$) ppm.

Allyl 6-*O*-benzyl-α-D-mannopyranosyl-(1→4)-6-*O*-benzyl-α-D-mannopyranosyl-(1→4)-6-*O*-benzyl-α-D-mannopyranosyl-(1→4)-6-*O*-benzyl-α-D-mannopyranoside (18)

To a solution of **17** (0.798 g, 0.55 mmol) in MeOH (7 mL), MeONa (17.8 mg, 0.33 mmol) was added. After complete conversion of the starting material, previously activated Dowex H^+ resin was added until neutral pH. After filtration with MeOH, the solvent was removed under vacuum to give compound **18** (0.561 g, quantitative, α anomer) as a colorless oil.

FTIR (ATR): 3396.0 (O-H) cm^{-1}.

^{1}H-NMR (MeOH-d4): δ 7.37 – 7.21 (m, 20H, Ar), 6.02 – 5.91 (m, 1H, $OCH_2C\underline{H}{=}CH_2$), 5.34 – 5.17 (m, 5H, H-1 A-C and $OCH_2CH{=}C\underline{H}_2$), 4.80 (d, 1H, *J* = 1.7 Hz, H-1 D), 4.58 – 4.30 (m, 8H, $OC\underline{H}_2Ph$ A-C), 4.25 – 4.18 (m, 1H, 1x$OC\underline{H}_2CH{=}CH_2$), 4.06 – 3.99 (m, 2H, H-4 A and 1x$OC\underline{H}_2CH{=}CH_2$), 3.95 – 3.64 (m, 23H, H-2 A-D, H-3 A-D, H-4 B-D, H-5 A-D and H-6 A or B or C or D), 3.57 (s, 2H, H-6 A or B or C or D) ppm.

^{13}C-NMR (MeOH-d4): δ 138.2 (C_q), 134.0 ($OCH_2\underline{C}H{=}CH_2$), 128.1 (Ar), 127.9 (Ar), 127.7 (Ar), 127.6 (Ar), 127.4 (Ar), 127.2 (Ar), 116.0 ($OCH_2CH{=}\underline{C}H_2$), 102.1 (C-1 A or B or C), 101.7 (C-1 A or B or C) 101.5 (C-1 A or B or C), 99.2 (C-1 D), 74.9, 74.7, 74.4, 73.2 ($O\underline{C}H_2Ph$ A or B or C or D), 73.1 ($O\underline{C}H_2Ph$ A or B or C or D), 73.0 ($O\underline{C}H_2Ph$ A or B or C or D), 72.8 ($O\underline{C}H_2Ph$ A or B or C or D), 71.9, 71.6, 71.5, 71.3, 71.2, 70.9, 70.6, 70.0 (C-6 A or B or C or D), 69.9 (C-6 A or B or C or D), 69.7 (C-6 A or B or C or D), 67.6 ($O\underline{C}H_2CH{=}CH_2$), 67.4 (C-4 A) ppm.

Propyl α-D-mannopyranosyl-(1→4)-α-D-mannopyranosyl-(1→4)-α-D-mannopyranosyl-(1→4)-α-D-mannopyranoside (1)

Compound **18** (0.520 g, 0.51 mmol) in AcOEt/EtOH (2:1 – 3 mL) was hydrogenated at 50 psi in the presence of Pd/C 10% (0.25 equiv). After 7 h, the reaction mixture was filtered through celite and the solvent was removed under vacuum to afford compound **1** (0.179 g, 52%, α anomer) as a viscous colorless foam.

FTIR (ATR): 3350.6 (O-H) cm^{-1}.

^{1}H-NMR (D_2O): δ 5.18 – 5.13 (m, 3H, H-1 A-C), 4.78 (s, 1H, H-1 D), 4.00 – 3.91 (m, 3H, H-2 A-C), 3.90 – 3.54 (m, 22H, H-2 D, H-3 A-D, H-4 A-D, H-5 A-D, H-6 A-D and 1xOC$\underline{H}_2$CH$_2$CH$_3$), 3.47 – 3.39 (m, 1H, 1xOC$\underline{H}_2$CH$_2$CH$_3$), 1.59 – 1.47 (m, 2H, OCH$_2$C$\underline{H}_2$CH$_3$), 0.84 (t, 3H, *J* = 7.4 Hz, OCH$_2$CH$_2$C$\underline{H}_3$) ppm.

^{13}C-NMR (D_2O): δ 101.4 (C-1 A or B or C), 101.3 (C-1 A or B or C), 99.5 (C-1 D), 74.3, 74.1, 74.1, 73.7, 72.2, 72.2, 71.1, 70.8, 70.7, 70.5, 70.3, 70.2, 69.6 (O$\underline{C}$H$_2$CH$_2$CH$_3$), 66.5, 61.0 (C-6 A or B or C or D) 60.9 (C-6 A or B or C or D), 60.8 (C-6 A or B or C or D), 21.9 (OCH$_2\underline{C}$H$_2$CH$_3$), 9.9 (OCH$_2$CH$_2\underline{C}$H$_3$) ppm.

HR-MS: calcd. for $C_{27}H_{49}O_{21}$ $[M + H]^+$: 709.2761; found: 709.2763.

Allyl 2,3,4-tri-*O*-acetyl-6-*O*-benzyl-α-D-mannopyranosyl-(1→4)-2,3-di-*O*-acetyl-6-*O*-benzyl-α-D-mannopyranosyl-(1→4)-2,3-di-*O*-acetyl-6-*O*-benzyl-α-D-mannopyranose (19)

A suspension of **11** (78.8 mg, 0.13 mmol), **13** (94 mg, 0.13 mmol) and 4Å MS in dry CH_2Cl_2 (3 mL) was stirred at rt for 30 min. TMSOTf (23.3 µL, 0.13 mmol) was added at -20 °C and the mixture was stirred until 0 °C. After 30 min, saturated $NaHCO_3$ aqueous solution (10 mL) was added, and the mixture was extracted with CH_2Cl_2 (3 x 10 mL). The combined organic phases were dried with anhydrous Na_2SO_4, filtered, and concentrated under vacuum. The crude product

was purified by flash column chromatography (7:3 Hex:AcOEt, v/v) to afford compound **19** (92 mg, 67%, α anomer) as a colorless oil.

FTIR (ATR): 3478.9 (O-H), 1747.2 (C=O) cm^{-1}.

^{1}H-NMR ($CDCl_3$): δ 7.38 – 7.20 (m, 15H, Ar), 5.97 – 5.83 (m, 1H, $OCH_2C\underline{H}=CH_2$), 5.36 – 5.28 (m, 3H, H-2 C, H-3 C, 1x$OCH_2CH=C\underline{H}_2$), 5.24 – 5.17 (m, 3H, H-2 A ou B, H-3 A ou B, 1x$OCH_2CH=C\underline{H}_2$), 5.13 – 5.01 (m, 4H, H-1 A-B, H-2 A or B, H-3 A or B), 4.84 (d, 1H, *J* = 1.4 Hz, H-1 C), 4.59 – 4.39 (m, 6H, $OC\underline{H}_2Ph$ A-C), 4.24 – 4.08 (m, 3H, H-4 A ou B, H-4 C, 1x$OC\underline{H}_2CH=CH_2$), 4.05 – 3.85 (m, 5H, H-4 A or B, H-5 C, H-6 A ou B, 1x$OC\underline{H}_2CH=CH_2$), 3.80 – 3.59 (m, 4H, H-5 A-B and H-6 C), 3.54 (dd, 2H, *J* = 10.1 Hz, *J* = 4.1 Hz, H-6 A or B), 2.11 – 2.00 (m, 21H, $(OCOC\underline{H}_3)_7$) ppm.

^{13}C NMR ($CDCl_3$): δ 169.8 (C=O), 133.2 ($OCH_2\underline{C}H=CH_2$), 128.4 (Ar), 128.3 (Ar), 127.8 (Ar), 127.7 (Ar), 127.4 (Ar), 127.3 (Ar), 118.2 ($OCH_2CH=\underline{C}H_2$), 99.5 (C-1 A or B), 99.1 (C-1 A or B), 96.4 (C-1 C), 74.0, 73.7 ($O\underline{C}H_2Ph$ A or B or C), 73.3 ($O\underline{C}H_2Ph$ A or B or C), 72.7, 71.9, 71.6, 71.4, 70.9, 70.0, 68.9, 68.4 ($O\underline{C}H_2CH=CH_2$), 20.9 ($OCO\underline{C}H_3$), 20.8 ($OCO\underline{C}H_3$), 20.7 ($OCO\underline{C}H_3$) ppm.

Allyl 6-*O*-benzyl-α-D-mannopyranosyl-(1→4)-6-*O*-benzyl-α-D-mannopyranosyl-(1→4)-6-*O*-benzyl-α-D-mannopyranoside (20)

To a solution of **19** (70 mg, 0.108 mmol) in MeOH (2 mL), MeONa (2.12 mg, 39.3 µmol) was added. After complete conversion of the starting material, previously activated Dowex H^+ resin was added until neutral pH. After filtration with MeOH, the solvent was removed under vacuum to give compound **20** (46 mg, 87%, α anomer) as a colorless oil.

FTIR (ATR): 3401.1 (O-H) cm^{-1}.

^{1}H-NMR (MeOH-d4): δ 7.39 – 7.21 (m, 15H, Ar), 6.01 – 5.90 (m, 1H, $OCH_2C\underline{H}=CH_2$), 5.34 – 5.16 (m, 4H, H-1 A-B and $OCH_2CH=C\underline{H}_2$), 4.79 (d, 1H, J = 1.6 Hz, H-1 C), 4.56 – 4.36 (m, 6H, $OC\underline{H}_2Ph$ A-C), 4.21 (dd, 1H, J = 7.9 Hz, J = 5.1 Hz, 1x$OC\underline{H}_2CH=CH_2$), 4.06 – 3.62 (m, 19H, 1x$OC\underline{H}_2CH=CH_2$, H-2 A-C, H-3 A-C, H-4 A-C, H-5 A-C and H-6 A-C) ppm.

^{13}C-NMR (MeOH-d4): δ 138.3 (C_q), 134.0 ($OCH_2\underline{C}H=CH_2$), 128.0 (Ar), 127.9 (Ar), 127.7 (Ar), 127.6 (Ar), 127.5 (Ar), 127.3 (Ar), 127.2 (Ar), 116.0 ($OCH_2CH=\underline{C}H_2$), 102.0 (C-1 A or B), 101.7 (C-1 A or B), 99.2 (C-1 C), 74.9, 74.8, 73.2 ($O\underline{C}H_2Ph$ A or B or C), 73.0 ($O\underline{C}H_2Ph$ A or B or C), 72.9, 72.8 ($O\underline{C}H_2Ph$ A or B or C), 71.8, 71.5, 71.3, 71.2, 71.1, 70.8, 70.7, 70.0 (C-6 A or B or C), 69.7 (C-6 A or B or C), 67.6 ($O\underline{C}H_2CH=CH_2$), 67.3 ppm.

Propyl-α-D-mannopyranosyl-(1→4)-α-D-mannopyranosyl-(1→4)-α-D-mannopyranoside (2)
Compound **20** (46 mg, 56.4 µmol) in AcOEt/EtOH (2:1 – 3 mL) was hydrogenated at 50 psi in the presence of Pd/C 10% (0.25 equiv). After 7 h, the reaction mixture was filtered through celite and the solvent was removed under vacuum to afford compound **2** (30 mg, quantitative, α anomer) as a viscous colorless foam.

FTIR (ATR): 3306.5 (O-H) cm^{-1}.

^{1}H-NMR (D_2O): δ 5.16 (d, J = 1.9 Hz, 2H, H-1 A and B), 4.78 (d, 1H, J = 1.1 Hz, H-1 C), 4.00 – 3.91 (m, 2H, H-2 A-B), 3.88 – 3.54 (m, 17H, H-2 C, H-3 A-C, H-4 A-C, H-5 A-C, H-6 A-C and 1x$OC\underline{H}_2CH_2CH_3$), 3.47 – 3.39 (m, 1H, 1x$OC\underline{H}_2CH_2CH_3$), 1.59 – 1.47 (m, 2H, $OCH_2C\underline{H}_2CH_3$), 0.83 (t, 3H, J = 7.4 Hz, $OCH_2CH_2C\underline{H}_3$) ppm.

^{13}**C-NMR** (D_2O): δ 101.4 (C-1 A or B), 101.3 (C-1 A or B), 99.5 (C-1 C), 74.3, 74.0, 73.7, 72.2, 71.1, 70.8, 70.7, 70.5 (C-2 C), 70.3 (C-2 A or B), 70.2 (C-2 A or B), 69.6 ($O\underline{C}H_2CH_2CH_3$), 66.5, 61.0 (C-6 A or B or C), 60.9 (C-6 A or B or C), 60.8 (C-6 A or B or C), 21.90 ($OCH_2\underline{C}H_2CH_3$), 9.84 ($OCH_2CH_2\underline{C}H_3$) ppm.

HR-MS: calcd. for $C_{21}H_{38}NaO_{16}$ $[M + Na]^+$: 569.2052; found: 569.2056.

Allyl 4,6-*O*-benzylidene-3-*O*-methyl-α-D-mannopyranoside (21)

To a solution of **6** (7.2 g, 23.4 mmol) in dry methanol (30 mL), dibutyltin oxide (6.7 g, 26.9 mmol) was added. The mixture was stirred under reflux for 3 h and the solvent was removed under vacuum. The crude product was re-dissolved in dry DMF (45 mL) and iodomethane (7.3 mL, 117 mmol) was added and the mixture was heated at 50 °C, overnight. The solvent was first removed under vacuum, then the mixture was re-dissolved in CH_2Cl_2 (30 mL) and the white solid formed was filtered. The solvent was removed under vacuum and the reaction mixture was purified by silica gel flash column chromatography (7:3 to 1:1 Hex:AcOEt, v/v) to afford compound **21** (4.83 g, 58%, α anomer) as a yellow oil.

FTIR (ATR): 3461.67 (O-H), 1646.98 (C=C), 1093.78-1034.83 (C-O) cm^{-1}.

NMR data for the α-anomer described according to the literature [2].

Allyl 2,4-di-*O*-acetyl-4,6-*O*-benzylidene-3-*O*-methyl-α-D-mannopyranoside (22)

To a solution of **21** (4.39 g, 13.6 mmol) in dry pyridine (30 mL) at 0 °C, distilled acetic anhydride (1.93 mL, 20.4 mmol) and a catalytic amount of DMAP were added. The mixture was stirred at 0 °C for 5 min and warmed to rt. After 2 h, the mixture was quenched with water (30 mL) and

extracted with AcOEt (3 x 30 mL). The combined organic layers were dried with Na_2SO_4, filtered, and concentrated under vacuum. The crude product was purified by silica gel flash column chromatography (7:3 Hex:AcOEt, v/v) to afford compound **22** (4.52 g, 92%, α anomer) as a colorless oil.

FTIR (ATR): 1746.45 (C=O), 1646.97 (C=C) cm^{-1}.

NMR data for the α-anomer described according to the literature [5].

Allyl 2-*O*-acetyl-6-*O*-benzyl-3-*O*-methyl-α-D-mannopyranoside (23)

To a solution of **22** (4.5 g, 12.4 mmol) in dry THF (35 mL) at 0 °C, $NaBH_3CN$ (9.36 g, 0.15 mol) was added. The mixture was stirred at 0 °C, and a solution of HCl in dry Et_2O (51 mL, 1M) was added dropwise until the reaction was completed as judged by TLC. The solvent was removed under vacuum, re-dissolved in water (20 mL) and extracted with CH_2Cl_2 (3 x 20 mL). The combined organic layers were dried with Na_2SO_4, filtered, and concentrated under vacuum. The crude product was purified by silica gel flash column chromatography (7:3 to 1:1 Hex:AcOEt, v/v) to afford compound **23** (3.54 g, 78%, α anomer) as a colorless oil.

FTIR (ATR): 3467.07 (O-H), 1744.5 (C=O), 1646.98 (C=C) cm^{-1}.

NMR data for the α-anomer described according to the literature [5].

Allyl 2-*O*-acetyl-6-*O*-benzyl-4-*O*-*tert*-butyldimethylsilyl-3-*O*-methyl-α-D-mannopyranoside (24)

To a solution of **23** (4.37 g, 11.9 mmol) in dry dichloromethane (15 or 20 mL) at 0 °C, DIPEA (5.8 mL, 33.3 mmol) and TBDMSOTf (3 mL, 13.1 mmol) were added sequentially. The mixture was stirred for 90 min at 0 °C. TLC (7:3 Hex:AcOEt, v/v) indicated that the reaction was

completed. The mixture was washed with a saturated aqueous solution of $NaHCO_3$ and extracted with CH_2Cl_2. The organic layer was dried with Na_2SO_4, filtered and concentrated. Purification of the reaction crude by silica gel flash column chromatography (eluent from 100% (v/v) Hex to 9:1 Hex:AcOEt, v/v) afforded **24** (3.65 g, 64%, α anomer) as a colorless oil.

FTIR (ATR): 1747.5 (C=O) cm^{-1}.

^{1}H-NMR ($CDCl_3$): δ 7.38 – 7.23 (m, 5H, Ar), 5.98 – 5.86 (m, 1H, $OCH_2C\underline{H}=CH_2$), 5.32 (s, 1H, H-2), 5.28 (d, 1H, *J* = 1.4 Hz, 1x$OCH_2CH=C\underline{H}_2$), 5.21 (d, 1H, *J* = 11.1 Hz, 1x$OCH_2CH=C\underline{H}_2$), 4.86 (s, 1H, H-1), 4.60 (q, 2H, *J* = 8 Hz, *J* = 12 Hz, $C\underline{H}_2Ph$), 4.21 (dd, 1H, *J* = 7.6 Hz, *J* = 5.2 Hz, 1x$OC\underline{H}_2CH=CH_2$), 4.05 – 3.97 (m, 1H, 1x$OC\underline{H}_2CH=CH_2$), 3.82 – 3.72 (m, 3H, H-4 and H-6), 3.72 – 3.66 (m, 1H, H-5), 3.46 – 3.40 (m, 1H, H-3), 3.30 (s, 3H, $OC\underline{H}_3$), 2.10 (s, 3H, $OCOC\underline{H}_3$), 0.87 (s, 9H, $SiC(C\underline{H}_3)_3$), 0.03 (d, *J* = 21.1 Hz, 6H, $Si(C\underline{H}_3)_2$) ppm.

^{13}C-NMR ($CDCl_3$): δ 170.5 (C=O), 138.5 (C_q), 133.6 ($OCH_2\underline{C}H=CH_2$) 128.4 (Ar), 128.3 (Ar), 127.9 (Ar), 127.7 (Ar), 127.4 (Ar), 117.8 ($OCH_2CH=\underline{C}H_2$) 96.8 (C-1), 79.8 (C-3), 73.2 ($\underline{C}H_2Ph$), 72.8 (C-5), 69.6 (C-6), 68.2 ($O\underline{C}H_2CH=CH_2$), 67.9 (C-4), 67.7 (C-2), 56.8 ($O\underline{C}H_3$), 26.0 ($SiC(\underline{C}H_3)_3$), 21.0 ($OCO\underline{C}H_3$), -4.05 ($Si(\underline{C}H_3)_2$) ppm.

2-*O*-acetyl-6-*O*-benzyl-4-*O*-*tert*-butyldimethylsilyl-3-*O*-methyl-(α/β)-D-mannopyranoside (25)

To a solution of **24** (3.2 g, 6.65 mmol) in dry methanol (30 mL), $PdCl_2$ (0.236 g, 1.33 mmol) was added. The mixture was stirred at rt for 2 h and then the mixture was filtered through celite and washed with methanol. The filtrate was concentrated under vacuum and purified by flash column chromatography (7:3 to 1:1 Hex:AcOEt, v/v), to provide compound **25** (1.47 g, 50%, 6:1 (α:β)) as a colorless oil.

^{1}H-NMR ($CDCl_3$): δ 7.40 – 7.26 (m, 5H, Ar), 5.35 (dd, 1H, *J* = 1.2 Hz, *J* = 2 Hz, H-2), 5.23 (s, 1H, H-1_α), 4.86 (s, 1H, H-1_β), 4.70 – 4.61 (m, 1H, 1xOC$\underline{H}_2$Ph), 4.59 – 4.52 (m, 1H, 1xOC$\underline{H}_2$Ph), 4.05 – 3.99 (m, 1H, H-5), 3.78 (dd, 1H, *J* = 8.4 Hz, *J* = 2 Hz, 1xH-6), 3.72 – 3.57 (m, 2H, H-4 and 1xH-6), 3.49 (dd, 1H, *J* = 6 Hz, *J* = 3.2 Hz, H-3), 3.32 (s, 3H, OC$\underline{H}_3$), 2.12 (s, 3H, OCOC$\underline{H}_3$), 0.82 (s, 9H, SiC(C$\underline{H}_3$)$_3$), 0.05 (m, 6H, Si(C$\underline{H}_3$)$_2$) ppm.

^{13}C-NMR ($CDCl_3$): δ 170.4 (C=O), 138.0 (C_q), 128.4 (Ar), 127.8 (Ar), 127.7 (Ar), 93.1 (C-1_α), 92.5 (C-1_β), 79.3 (C-3), 73.4 (O$\underline{C}H_2$Ph), 72.5 (C-5), 69.9 (C-6), 68.2 (C-4), 67.9 (C-2), 56.8 (O$\underline{C}H_3$), 25.9 (SiC($\underline{C}H_3$)$_3$), 21.0 (OCO$\underline{C}H_3$), -4.1 (Si($\underline{C}H_3$)$_2$) ppm.

2-*O*-acetyl-6-*O*-benzyl-4-*O*-*tert*-butyldimethylsilyl-3-*O*-methyl-α-D-mannopyranoside trichloroacetimidate (26)

To a solution of **25** (0.714 g, 1.62 mmol) in dry CH_2Cl_2 (7 mL) at 0 °C, DBU (110 µL, 0.71 mmol) and trichloroacetonitrile (0.474 mL, 4.73 mmol) were added. The mixture was stirred for 10 min at 0 °C, warmed to rt and stirred for 2 h. The solvent was evaporated under vacuum and the crude product was purified by silica gel flash column chromatography (8:2 Hex:AcOEt, v/v) to afford compound **26** (0.54 g, 57%) as a colorless oil.

^{1}H-NMR ($CDCl_3$): δ 8.69 (s, 1H, OC(N$\underline{H}$)CCl_3), 7.38 – 7.23 (m, 5H, Ar), 6.29 (d, 1H, *J* = 1.6 Hz, H-1), 5.51 – 5.48 (m, 1H, H-2), 4.59 (s, 2H, OC$\underline{H}_2$Ph), 4.00 - 3.91 (m, 2H, H-4 and H-5), 3.75 (d, 2H, *J* = 2.2 Hz, H-6), 3.47 (dd, 1H, *J* = 5.6 Hz, *J* = 2.8 Hz, H-3), 3.33 (s, 3H, OC$\underline{H}_3$), 2.13 (s, 3H, OCOC$\underline{H}_3$), 0.84 (s, 9H, SiC(C$\underline{H}_3$)$_3$), 0.05 (d, 6H, *J* = 19.5 Hz, Si(C$\underline{H}_3$)$_2$) ppm.

^{13}**C-NMR** ($CDCl_3$): δ 170.1 (C=O), 160.0 (C=O), 138.4 (C_q), 128.4 (Ar), 128.2 (Ar), 127.8 (Ar), 127.7 (Ar), 127.6 (Ar), 127.4 (Ar), 95.4 (C-1), 79.9 (C-3), 75.8 (C-5), 73.2 (O$\underline{C}H_2$Ph), 68.9 (C-6), 67.1 (C-4), 66.1 (C-2), 57.0 (O$\underline{C}H_3$), 26.0 (SiC($\underline{C}H_3$)$_3$), 20.9 (OCO$\underline{C}H_3$), -4.1 (Si($\underline{C}H_3$)$_2$) ppm.

Allyl 2,4-di-*O*-acetyl-6-*O*-benzyl-3-*O*-methyl-α-D-mannopyranoside (27)

To a solution of **23** (1.72 g, 4.68 mmol) in dry pyridine (5 mL) at 0 °C, acetic anhydride (0.66 mL, 7.02 mmol) and a catalytic amount of DMAP were added. The mixture was stirred at 0 °C for 5 min, warmed to rt and stirred for 90 min. The mixture was quenched with water (20 mL) and extracted with AcOEt (3 x 20 mL). The combined organic layers were dried with Na_2SO_4, filtered, and concentrated under vacuum. The crude product was purified by silica gel flash column chromatography (7:3 Hex:AcOEt, v/v) to afford **27** (1.26 g, 83%, α anomer) as a colorless oil.

FTIR (ATR): 1743.29 (C=O), 1647.14 (C=C) cm^{-1}.

NMR data for the α-anomer described according to the literature [5].

2,4-Di-*O*-acetyl-6-*O*-benzyl-3-*O*-methyl-(α/β)-D-mannopyranoside (28)

To a solution of **27** (1.23 g, 3.02 mmol) in dry methanol (10 mL), $PdCl_2$ (0.107 g, 0.604 mmol) was added. The mixture was stirred at rt for 2 h and then the mixture was filtered through celite and washed with methanol. The filtrate was concentrated under vacuum and purified by flash column chromatography (7:3 to 1:1 Hex:AcOEt, v/v) to afford compound **28** (0.99 g, 89%, 15:1 (α/β)) as a colorless oil.

FTIR (ATR): 3419.51 (O-H), 1743.65 (C=O) cm^{-1}.

NMR data for the α-anomer described according to the literature [5].

2,4-Di-*O*-acetyl-6-*O*-benzyl-3-*O*-methyl-α-D-mannopyranosyl trichloroacetimidate (29)

To a solution of **28** (0.96 g, 2.6 mmol) in dry CH_2Cl_2 (10 mL) at 0 °C, DBU (0.171 mL, 1.14 mmol) and trichloroacetonitrile (1.3 mL, 13 mmol) were added. The mixture was stirred for 10 min at 0 °C, warmed to rt and stirred for 2 h. The solvent was evaporated under vacuum and the crude product was purified by flash column chromatography (7:3 Hex:AcOEt, v/v) to afford compound **29** (1.27 g, 70%) as a colorless oil.

$[\alpha]_D^{20}$ +38.8 (c 0.95, CH_2Cl_2).

FTIR (ATR): 3316.9 (N-H), 1748.9 (C=O) cm^{-1}.

NMR data for the α-anomer described according to the literature [5].

Allyl 2-*O*-acetyl-6-*O*-benzyl-3-*O*-methyl-α-D-mannopyranosyl-(1→4)-2-*O*-acetyl-6-*O*-benzyl-3-*O*-methyl-α-D-mannopyranoside (30)

A suspension of **26** (0.51 g, 0.87 mmol), **23** (0.35 g, 0.87 mmol) and 4Å MS in dry CH_2Cl_2 (30 mL) was stirred at rt for 30 min. TMSOTf (0.16 mL, 0.87 mmol) was added at -20 °C and the mixture was stirred until 0 °C. After 30 min, saturated $NaHCO_3$ aqueous solution (30 mL) was added, and the mixture was extracted with CH_2Cl_2 (3 x 10 mL). The combined organic phases were dried with anhydrous Na_2SO_4, filtered, and concentrated under vacuum. The crude product was purified by flash column chromatography (7:3 Hex:AcOEt, v/v) to afford compound **30** (0.274 g, 48%, α anomer) as a colorless oil.

FTIR (ATR): 3468.2 (O-H), 1743.5 (C=O) cm^{-1}.

^{1}H-NMR ($CDCl_3$): δ 7.38 – 7.20 (m, 10H, Ar), 5.98 – 5.83 (m, 1H, $OCH_2C\underline{H}=CH_2$), 5.36 (dd, 1H, *J* = 1.2 Hz, *J* = 2.0 Hz, H-2 A), 5.34 – 5.27 (m, 2H, H-2 B and 1x$OCH_2CH=C\underline{H_2}$), 5.24 (d, 1H, *J* = 1.6 Hz, H-1 A), 5.21 (d, 2H, *J* = 1.6 Hz, 1x$OCH_2CH=C\underline{H_2}$), 4.86 (d, 1H, *J* = 1.6 Hz, H-1 B), 4.60 – 4.44 (m, 4H, $OC\underline{H_2}Ph$ A-B), 4.24 – 4.15 (m, 1H, 1x$OC\underline{H_2}CH=CH_2$), 4.05 – 3.98 (m, 1H, 1x$OC\underline{H_2}CH=CH_2$), 3.96 – 3.73 (m, 6H, H-4 A-B, H-5 A-B and H-6 B), 3.70 – 3.65 (m, 1H, H-3 B), 3.65 – 3.55 (m, 2H, H-6 A), 3.48 – 3.44 (m, 1H, H-3 A), 3.42 (s, 3H, $OC\underline{H_3}$ A or B), 3.40 (s, 3H, $OC\underline{H_3}$ A or B), 2.09 (m, 6H, *J* = 0.8 Hz, $(OCOC\underline{H_3})_2$) ppm.

^{13}C-NMR ($CDCl_3$): δ 170.3 (C=O), 170.1 (C=O), 138.4 (C_q), 138.1 (C_q), 133.4 ($OCH_2\underline{C}H=CH_2$), 128.6 (Ar), 128.3 (Ar), 127.6 (Ar), 127.4 (Ar), 118.0 ($OCH_2CH=\underline{C}H_2$), 99.7 (C-1 A), 96.7 (C-1 B), 80.1 (C-3 B), 79.1 (C-3 A), 73.6 ($O\underline{C}H_2Ph$ A or B), 73.5 (C-4 B), 73.3 ($O\underline{C}H_2Ph$ A or B), 72.0 (C-5 A or B), 70.9 (C-5 A or B), 70.1 (C-6 A), 69.6 (C-6 B), 68.4 ($O\underline{C}H_2CH=CH_2$), 67.6 (C-4 A), 67.6 (C-2 B), 67.3 (C-2 A), 57.4 ($O\underline{C}H_3$ A or B), 57.2 ($O\underline{C}H_3$ A or B), 21.0 ($OCO\underline{C}H_3$) ppm.

Allyl 2,4-di-*O*-acetyl-6-*O*-benzyl-3-*O*-methyl-α-D-mannopyranosyl-(1→4)-2-*O*-acetyl-6-*O*-benzyl-3-*O*-methyl-α-D-mannopyranoside (31)

A suspension of **29** (0.91 g, 1.77 mmol), **23** (0.65 g, 1.77 mmol) and 4Å MS in CH_2Cl_2 (10 mL) was stirred at rt for 30 min. TMSOTf (319 μL, 1.77 mmol) was added at -20 °C and the mixture was stirred until 0 °C. After 30 min, saturated $NaHCO_3$ aqueous solution (10 mL) was added, and the mixture was extracted with CH_2Cl_2 (3 x 10 mL). The combined organic phases were dried with Na_2SO_4, filtered, and concentrated under vacuum. The crude product was purified by flash column chromatography (7:3 Hex:AcOEt, v/v) to afford compound **31** (0.81 g, 65%, α anomer) as a colorless oil.

FTIR (ATR): 1746.63 (C=O), 1652.68 (C=C) cm^{-1}.

NMR data for the α-anomer described according to the literature [5].

2,4-Di-*O*-acetyl-6-*O*-benzyl-3-*O*-methyl-α-D-mannopyranosyl-(1→4)-2-*O*-acetyl-6-*O*-benzyl-3-*O*-methyl-α-D-mannopyranose (32)

To a solution of **31** (0.78 g, 1.09 mmol) in MeOH (5 mL), $PdCl_2$ (38.7 mg ,0.22 mmol) was added. After 2h at rt, the mixture was filtered through celite and washed with MeOH. The crude product was purified by silica gel flash column chromatography (1:1 Hex:AcOEt, v/v) to afford compound **32** (0.54 g, 73%, α anomer) as a colorless oil.

FTIR (ATR): 3418.6 (O-H),1743.5 (C=O) cm^{-1}.

NMR data for the α-anomer described according to the literature [5].

2,4-Di-*O*-acetyl-6-*O*-benzyl-3-*O*-methyl-α-D-mannopyranosyl-(1→4)-2-*O*-acetyl-6-*O*-benzyl-3-*O*-methyl-α-D-mannopyranosyl trichloroacetimidate (33)

To a solution of **32** (0.59 g, 0.87 mmol) in dry CH_2Cl_2 (7 mL) at 0 °C, DBU (110 μL, 0.71 mmol) and trichloroacetonitrile (0.474 mL, 4.73 mmol) were added. The mixture was stirred for 10 min at 0 °C, warmed to rt and stirred for 2 h. The solvent was evaporated under vacuum and the crude product was purified by flash column chromatography (8:2 Hex:AcOEt, v/v) to afford compound **33** (0.504 g, 68%) as a colorless oil.

$[\alpha]_D^{20}$ +28.8 (c 1, CH_2Cl_2).

FTIR (ATR): 3302.9 (O-H), 1747.1 (C=O) cm^{-1}.

^{1}H-NMR ($CDCl_3$): δ 8.74 (s, 1H, OC(N$\underline{H}$)CCl_3), 7.35 – 7.21 (m, 10H, Ar), 6.29 (d, 1H, *J* = 1.6 Hz, H-1 B), 5.51 – 5.46 (m, 1H, H-2 B), 5.41 – 5.38 (m, 1H, H-2 A), 5.30 (d, 1H, *J* = 2 Hz, H-1 A), 5.19 (t, 1H, *J* = 10.0 Hz, H-4 B), 4.60 – 4.36 (m, 4H, OC$\underline{H}_2$Ph A- B), 4.08 – 3.97 (m, 2H, H-4 A and H-5 A), 3.91 – 3.82 (m, 1H, H-5 B), 3.82 – 3.73 (m, 2H, H-6 A), 3.71 (dd, 1H, *J* = 5.6 Hz, *J* = 3.4 Hz, H-3 A), 3.58 (dd, 1H, *J* = 6.4 Hz, *J* = 3.2 Hz, H-3 B), 3.49 – 3.40 (m, 5H, H-6 B and OC$\underline{H}_3$ A or B), 3.39 – 3.33 (m, 3H, OC$\underline{H}_3$ A or B), 2.11 (d, 6H, *J* = 3.4 Hz, (OCOC$\underline{H}_3$)$_2$), 1.97 (s, 3H, OCOC$\underline{H}_3$) ppm.

^{13}C-NMR ($CDCl_3$): δ 170.2 (C=O), 169.9 (C=O), 138.4 (C_q), 138.0 (C_q), 128.5 (Ar), 128.3 (Ar), 128.2 (Ar), 127.8 (Ar), 127.6 (Ar), 127.5 (Ar), 99.4 (C-1 A), 95.1 (C-1 B), 80.0 (C-3 A), 76.7 (C-3 B), 73.9 (C-5 A), 73.5 (O$\underline{C}H_2$Ph A or B), 73.3 (O$\underline{C}H_2$Ph A or B), 72.6 (C-4 A), 70.8 (C-5 B), 69.3 (C-6 A), 69.2 (C-6 B), 68.2 (C-4 A), 67.7 (C-2 A), 66.0 (C-2 B), 57.6 (O$\underline{C}H_3$ A or B), 57.2 (O$\underline{C}H_3$ A or B), 21.0 (OCO$\underline{C}H_3$), 20.9 (OCO$\underline{C}H_3$), 20.8 (OCO$\underline{C}H_3$) ppm.

Allyl 2,4-di-*O*-acetyl-6-*O*-benzyl-3-*O*-methyl-α-D-mannopyranosyl-(1→4)-2-*O*-acetyl-6-*O*-benzyl-3-*O*-methyl-α-D-mannopyranosyl-(1→4)-2-*O*-acetyl-6-*O*-benzyl-3-*O*-methyl-α-D-mannopyranosyl-(1→4)-2-*O*-acetyl-6-*O*-benzyl-3-*O*-methyl-α-D-mannopyranoside (34)

A suspension of **33** (0.304 g, 0.371 mmol), **30** (0.25 g, 0.371 mmol) and 4Å MS in dry CH_2Cl_2 (8 mL) was stirred at rt for 30 min. TMSOTf (70 μL, 0.371 mmol) was added at -30 °C and the mixture was stirred until 0 °C. After 30 min, saturated $NaHCO_3$ aqueous solution (10 mL) was added, and the mixture was extracted with CH_2Cl_2 (3 x 10 mL). The combined organic phases were dried with anhydrous Na_2SO_4, filtered, and concentrated under vacuum. The crude product was purified by flash column chromatography (7:3 Hex:AcOEt, v/v) to afford compound **34** (0.423 g, 86%, α anomer) as a colorless oil.

FTIR (ATR): 1743.3 (C=O) cm^{-1}.

^{1}H-NMR ($CDCl_3$): δ 7.40 – 7.17 (m, 20H, Ar), 5.98 – 5.85 (m, 1H, $OCH_2C\underline{H}=CH_2$), 5.43 – 5.14 (m, 10H, H-1 A-C, H-2 A-D, H-4 A, $OCH_2CH=C\underline{H}_2$), 4.86 (d, 1H, *J* = 1.6 Hz, H-1 D), 4.65 – 4.37 (m, 8H, $OC\underline{H}_2Ph$ A-D) 4.21 (dd, 1H, *J* = 7.2 Hz, *J* = 5.6 Hz, 1x$OC\underline{H}_2CH=CH_2$), 4.02 (dd, 1H, *J* = 12.8 Hz, *J* = 6.2 Hz, 1x$OC\underline{H}_2CH=CH_2$), 3.96 – 3.51 (m, 17H, H-3 A-D, H-4 A-D, H-5 A-D and H-6 A-C), 3.47 – 3.27 (m, 14H, H-6 D and $OC\underline{H}_3$ A-D), 2.16 – 1.95 (m, 15H, $(OCOC\underline{H}_3)_5$) ppm.

^{13}C-NMR ($CDCl_3$): δ 170.3 (C=O), 170.1 (C=O), 170.0 (C=O), 169.9 (C=O), 169.8 (C=O), 138.5 (C_q), 138.0 (C_q), 133.4 ($OCH_2\underline{C}H=CH_2$), 128.5 (Ar), 128.4 (Ar), 128.3 (Ar), 128.2 (Ar), 128.1 (Ar), 127.8 (Ar), 127.7 (Ar), 127.6 (Ar), 127.5 (Ar), 127.4 (Ar), 118.1 ($OCH_2CH=\underline{C}H_2$), 99.7 (C-1 A or B or C), 99.6 (C-1 A or B or C), 99.4 (C-1 A or B or C), 96.7 (C-1 D), 79.9 (C-3 A or B or C or D), 79.7 (C-3 A or B or C or D), 79.0 (C-3 A or B or C or D), 76.9 (C-3 A or B or C or D), 74.7, 74.2, 73.6, 73.5 ($O\underline{C}H_2Ph$ A or B or C or D), 73.4 ($O\underline{C}H_2Ph$ A or B or C or D), 73.3 ($O\underline{C}H_2Ph$ A or B or C or D), 73.2 ($O\underline{C}H_2Ph$ A or B or C or D), 71.9, 71.8, 71.1, 70.8, 70.0 (C-6 A or B or C or D), 69.9 (C-6 A or B or C or D), 69.8 (C-6 A or B or C or D), 69.4 (C-6 A or B or C or D), 68.5 ($O\underline{C}H_2CH=CH_2$), 68.3 (C-4 A), 67.7 (C-2 A or B or C or D), 67.5 (C-2 A or B or C or D), 67.4 (C-2 A or B or C or D), 67.3 (C-2 A or B or C or D), 57.5 ($O\underline{C}H_3$ A or B or C or D), 57.2 ($O\underline{C}H_3$ A or B or C or D), 57.1 ($O\underline{C}H_3$ A or B or C or D), 57.0 ($O\underline{C}H_3$ A or B or C or D), 21.0 ($OCO\underline{C}H_3$), 20.9 ($OCO\underline{C}H_3$) ppm.

Allyl 6-*O*-benzyl-3-*O*-methyl-α-D-mannopyranosyl-(1→4)-6-*O*-benzyl-3-*O*-methyl-α-D-mannopyranosyl-(1→4)-6-*O*-benzyl-3-*O*-methyl-α-D-mannopyranosyl-(1→4)-6-*O*-benzyl-3-*O*-methyl-α-D-mannopyranoside (35)

To a solution of **34** (408 mg, 0.304 mmol) in MeOH (5 mL), MeONa (9.84 mg, 0.18 mmol) was added. After complete conversion of the starting material, previously activated Dowex H^+ resin was added until neutral pH. After filtration with MeOH, the solvent was removed under vacuum to give compound **35** (285 mg, 84%, α anomer) as a colorless oil.

FTIR (ATR): 3422.7 (O-H) cm^{-1}.

^{1}H-NMR (MeOH-d4): δ 7.41 – 7.16 (m, 20H, Ar), 6.03 – 5.92 (m, 1H, $OCH_2C\underline{H}=CH_2$), 5.32 (dd, 1H, *J* = 15.6 Hz, *J* = 1.6 Hz, $1xOCH_2CH=C\underline{H}_2$), 5.23 – 5.16 (m, 4H, H-1 A-C and $1xOCH_2CH=C\underline{H}_2$), 4.85 (s, 1H, H-1 D), 4.58 – 4.31 (m, 8H, $OC\underline{H}_2Ph$ A-D), 4.23 (dd, 1H, *J* = 8 Hz, *J* = 5 Hz, $1xOC\underline{H}_2CH=CH_2$), 4.17 – 4.00 (m, 5H, $1xOC\underline{H}_2CH=CH_2$ and H-2 A-D) 3.97 – 3.64 (m, 15H, H-4 A-D, H-5 A-D, H-6 A or B or C or D), 3.57 (d, 1H, *J* = 4.1 Hz, 1xH-6 A or B or C or D), 3.55 – 3.49 (m, 2H, H-3 A or B or C or D), 3.49 – 3.38 (m, 14H, $OC\underline{H}_3$ A-D and H-3 A or B or C or D) ppm.

^{13}C-NMR (MeOH-d4): δ 128.1 (Ar) 127.9 (Ar), 127.7 (Ar), 127.6 (Ar), 127.2 (Ar), 116.3 ($OCH_2CH=\underline{C}H_2$), 102.4 (C-1 A or B or C), 102.0 (C-1 A or B or C), 101.8 (C-1 A or B or C), 99.1 (C-1 D), 81.9 (C-3 A or B or C or D), 81.5 (C-3 A or B or C or D), 80.8 (C-3 A or B or C or D) 73.6, 73.4, 73.3, 73.2 ($O\underline{C}H_2Ph$ A or B or C or D), 73.1 ($O\underline{C}H_2Ph$ A or B or C or D), 73.0 ($O\underline{C}H_2Ph$ A or B or C or D), 72.8 ($O\underline{C}H_2Ph$ A or B or C or D), 71.8, 71.6, 71.5, 70.7, 70.0 (C-6 A or B or C or D), 69.9 (C-6 A or B or C or D), 67.8 ($O\underline{C}H_2CH=CH_2$), 66.7 (C-2 A or B or C or D), 66.3 (C-2 A or B or C or D), 66.1 (C-2 A or B or C or D), 56.0 ($O\underline{C}H_3$ A or B or C or D), 55.3 ($O\underline{C}H_3$ A or B or C or D) 55.2 ($O\underline{C}H_3$ A or B or C or D) ppm.

Propyl 3-*O*-methyl-α-D-mannopyranosyl-(1→4)-3-*O*-methyl-α-D-mannopyranosyl-(1→4)-3-*O*-methyl-α-D-mannopyranosyl-(1→4)-3-*O*-methyl-α-D-mannopyranoside (3)

Compound **35** (246 mg, 219 µmol) in AcOEt/EtOH (2:1 – 3 mL) was hydrogenated at 50 psi in the presence of Pd/C 10% (0.25 equiv). After 7 h, the reaction mixture was filtered through celite and the solvent was removed under vacuum to afford compound **3** (160 mg, quantitative, α anomer) as a viscous colorless foam.

FTIR (ATR): 3306.2 (O-H) cm^{-1}.

^{1}**H-NMR** (D_2O): δ 5.14 (s, 3H, H-1 A-C), 4.83 (d, 1H, *J* = 1.5 Hz, H-1 D), 4.20 – 4.04 (m, 4H, H-2 A-D), 3.86 – 3.56 (m, 20H, H-4 A-D, H-5 A-D, H-6 A-D, 1x$OC\underline{H}_2CH_2CH_3$, H-3 A or B or C or D), 3.48 – 3.32 (m, 14H, $OC\underline{H}_3$ A-D, 1x$OC\underline{H}_2CH_2CH_3$ and H-3 A or B or C or D), 1.59 – 1.46 (m, 2H, $OCH_2C\underline{H}_2CH_3$), 0.85 (t, 3H, *J* = 7.6 Hz, $OCH_2CH_2C\underline{H}_3$) ppm.

^{13}**C-NMR** (D_2O): δ 101.3 (C-1 A or B or C), 101.2 (C-1 A or B or C), 101.1 (C-1 A or B or C), 99.4 (C-1 D), 81.0, 80.7, 79.7, 73.7, 72.6, 72.3, 72.2, 72.1, 71.0, 69.7 ($O\underline{C}H_2CH_2CH_3$), 66.1 (C-2 A or B or C or D), 66.0 (C-2 A or B or C or D), 65.8 (C-2 A or B or C or D), 65.4 (C-2 A or B or C or D), 60.9 (C-6 A or B or C or D), 60.8 (C-6 A or B or C or D), 56.1 ($O\underline{C}H_3$ A-D), 21.9 ($OCH_2\underline{C}H_2CH_3$), 9.7 ($OCH_2CH_2\underline{C}H_3$) ppm.

HR-MS: calcd. for $C_{31}H_{57}O_{21}$ $[M + H]^+$: 765.3387; found: 765.3388.

Allyl 2,4-di-*O*-acetyl-6-*O*-benzyl-3-*O*-methyl-α-D-mannopyranosyl-(1→4)-2-*O*-acetyl-6-*O*-benzyl-3-*O*-methyl-α-D-mannopyranosyl-(1→4)-2-*O*-acetyl-6-*O*-benzyl-3-*O*-methyl-α-D-mannopyranoside (36)

A suspension of **30** (207 mg, 0.252 mmol), **23** (92.4 mg, 0.252 mmol) and 4Å MS in dry CH_2Cl_2 (4 mL) was stirred at rt for 30 min. TMSOTf (45.7 µL, 0.13 mmol) was added at -30 °C and the mixture was stirred until 0 °C. After 30 min, saturated $NaHCO_3$ aqueous solution (10 mL) was

added, and the mixture was extracted with CH_2Cl_2 (3 x 10 mL). The combined organic phases were dried with anhydrous Na_2SO_4, filtered, and concentrated under vacuum. The crude product was purified by flash column chromatography (7:3 Hex:AcOEt, v/v) to afford compound **36** (219 mg, 85%, α anomer) as a colorless oil.

FTIR (ATR): 1743.8 (C=O) cm^{-1}.

^{1}H-NMR ($CDCl_3$): δ 7.36 – 7.21 (m, 15H, Ar), 5.99 – 5.83 (m, 1H, $OCH_2C\underline{H}=CH_2$), 5.40 – 5.36 (m, 2H, H-2 A-B), 5.34 – 5.30 (m, 1H, H-2 C), 5.29 (d, 1H, *J* = 1.5 Hz, $1xOCH_2CH=C\underline{H}_2$), 5.24 (s, 1H, H-1 A or B), 5.23 – 5.21 (m, 1H, $1xOCH_2CH=C\underline{H}_2$), 5.17 (dd, 2H, *J* = 2 Hz, *J* = 4 Hz,H-1 A or B and H-4 A), 4.86 (d, 1H, *J* = 1.7 Hz, H-1 C) , 4.66 – 4.38 (m, 6H, $OC\underline{H}_2Ph$ A-C), 4.25 – 4.18 (m, 1H, $1xOC\underline{H}_2CH=CH_2$), 4.02 (dd, 1H, *J* = 6.4 Hz, *J* = 6.4 Hz, $1xOC\underline{H}_2CH=CH_2$), 3.94 – 3.54 (m, 12H, H-3 A-C, H-4 B-C, H-5 A-C and H-6 A or B or C), 3.49 – 3.31 (m, 11H, $OC\underline{H}_3$ A-C and H-6 A or B or C), 2.12 – 2.04 (m, 9H, $(OCOC\underline{H}_3)_3$), 1.98 (s, 3H, $OCOC\underline{H}_3$) ppm.

^{13}C-NMR ($CDCl_3$): δ 170.1 (C=O), 170.0 (C=O), 138.5 (C_q), 138.0 (C_q), 133.4 ($OCH_2\underline{C}H=CH_2$), 128.3 (Ar), 128.2 (Ar), 127.8 (Ar), 127.6 (Ar), 127.4 (Ar), 118.0 ($OCH_2CH=\underline{C}H_2$), 99.7 (C-1 A or B), 99.4 (C-1 A or B), 96.7 (C-1 C), 79.9 (C-3 A or B or C), 79.8 (C-3 A or B or C), 76.8 (C-3 A or B or C), 74.6, 73.6, 73.5 ($O\underline{C}H_2Ph$ A or B or C), 73.4 ($O\underline{C}H_2Ph$ A or B or C), 73.3 ($O\underline{C}H_2Ph$ A or B or C), 71.9, 71.0, 70.8, 69.9 (C-6 A or B or C), 69.4 (C-6 A or B or C), 68.5 ($O\underline{C}H_2CH=CH_2$), 68.3 (C-4 A), 67.7 (C-2 A or B or C), 67.5 (C-2 A or B or C), 67.4 (C-2 A or B or C), 57.6 ($O\underline{C}H_3$ A or B or C), 57.2 ($O\underline{C}H_3$ A or B or C), 57.1 ($O\underline{C}H_3$ A or B or C), 21.0 ($OCO\underline{C}H_3$), 20.9 ($OCO\underline{C}H_3$) ppm.

Allyl 6-*O*-benzyl-3-*O*-methyl-α-D-mannopyranosyl-(1→4)-6-*O*-benzyl-3-*O*-methyl-α-D-mannopyranosyl-(1→4)-6-*O*-benzyl-3-*O*-methyl-α-D-mannopyranoside (37)

To a solution of **36** (111 mg, 0.108 mmol) in MeOH (2 mL), MeONa (3.5 mg, 65 μmol) was added. After complete conversion of the starting material, previously activated Dowex H^+ resin was added until neutral pH. After filtration with MeOH, the solvent was removed under vacuum to give compound **37** (70 mg, 76%, α anomer) as a colorless oil.

FTIR (ATR): 3415.7 (O-H) cm^{-1}.

^{1}H-NMR (MeOH-d4): δ 7.42 – 7.22 (m, 15H, Ar), 6.03 – 5.90 (m, 1H, $OCH_2C\underline{H}=CH_2$), 5.43 – 5.12 (m, 4H, H-1 A-B and $OCH_2CH=C\underline{H_2}$), 4.84 (d, 1H, J = 1.6 Hz, H-1 C), 4.69 – 4.35 (m, 6H, $OC\underline{H_2}Ph$ A-C), 4.26 – 4.16 (m, 1H, $1xOC\underline{H_2}CH=CH_2$), 4.15 – 3.99 (m, 4H, H-2 A-C and $1xOC\underline{H_2}CH=CH_2$), 3.95 – 3.61 (m, 12H, H-4 A-C, H-5 A-C and H-6 A-C), 3.59 – 3.36 (m, 12H, OCH_3 A-C) ppm.

^{13}C-NMR (MeOH-d4): δ 133.9 ($OCH_2\underline{C}H=CH_2$), 128.0 (Ar), 127.9 (Ar), 127.6 (Ar), 127.5 (Ar), 127.3 (Ar), 127.2 (Ar), 127.1 (Ar), 116.3 ($OCH_2CH=\underline{C}H_2$), 102.3 (C-1 A or B), 102.0 (C-1 A or B), 99.1 (C-1 C), 81.8 (C-3 A or B or C), 81.5 (C-3 A or B or C), 80.7 (C-3 A or B or C), 73.7, 73.5, 73.2 ($O\underline{C}H_2Ph$ A or B or C), 73.1 ($O\underline{C}H_2Ph$ A or B or C), 72.9 ($O\underline{C}H_2Ph$ A or B or C), 71.8, 70.8, 69.9 (C-6 A or B or C), 69.8 (C-6 A or B or C), 69.6 (C-6 A or B or C), 67.8 ($O\underline{C}H_2CH=CH_2$), 66.7 (C-2 A or B or C), 66.4 (C-2 A or B or C), 66.1 (C-2 A or B or C), 66.0, 55.9 ($O\underline{C}H_3$ A or B or C), 55.3 ($O\underline{C}H_3$ A or B or C) ppm.

Propyl 3-*O*-methyl-α-D-mannopyranosyl-(1→4)-3-*O*-methyl-α-D-mannopyranosyl-(1→4)-3-*O*-methyl-α-D-mannopyranoside (4)

Compound **37** (50 mg, 58.3 µmol) in AcOEt/EtOH (2:1 – 3 mL) was hydrogenated at 50 psi in the presence of Pd/C 10% (0.25 equiv). After 7 h, the reaction mixture was filtered through celite and the solvent was removed under vacuum to afford compound **4** (37 mg, quantitative, α anomer) as a viscous colorless foam.

FTIR (ATR): 3351.8 (O-H) cm^{-1}.

^{1}H-NMR (D_2O): δ 5.15 – 5.12 (m, 2H, H-1 A and B), 4.87 – 4.82 (m, 1H, H-1 C), 4.16 – 4.09 (m, 3H, H-2 A-C), 3.83 – 3.53 (m, 17H, H-3 A-C, H-4 A-C, H-5 A-C, H-6 A-C and $OC\underline{H}_2CH_2CH_3$), 3.47 – 3.33 (m, 9H, $OC\underline{H}_3$ A-C), 1.58 – 1.51 (m, 2H, $OCH_2C\underline{H}_2CH_3$), 0.85 (dd, 3H, J = 12.4 Hz, J = 5.1 Hz, $OCH_2CH_2C\underline{H}_3$).

^{13}C-NMR (D_2O): δ 101.3 (C-1 A or B), 101.2 (C-1 A or B), 99.4 (C-1 C), 81.0, 80.7, 80.2, 80.0, 79.7, 73.7, 72.7, 72.3, 72.2, 71.0, 69.7 ($O\underline{C}H_2CH_2CH_3$), 69.5, 66.1, 66.0, 65.8, 65.4, 60.9(C-6 A or B or C), 60.8 (C-6 A or B or C), 56.2 ($O\underline{C}H_3$ A or B or C), 56.1 ($O\underline{C}H_3$ A or B or C), 56.0 ($O\underline{C}H_3$ A or B or C), 21.9 ($OCH_2\underline{C}H_2CH_3$), 9.9 ($OCH_2CH_2\underline{C}H_3$).

HR-MS: calcd for $C_{24}H_{44}NaO_{16}$ $[M + Na]^+$: 611.2522; found: 611.2521.

Scheme 1

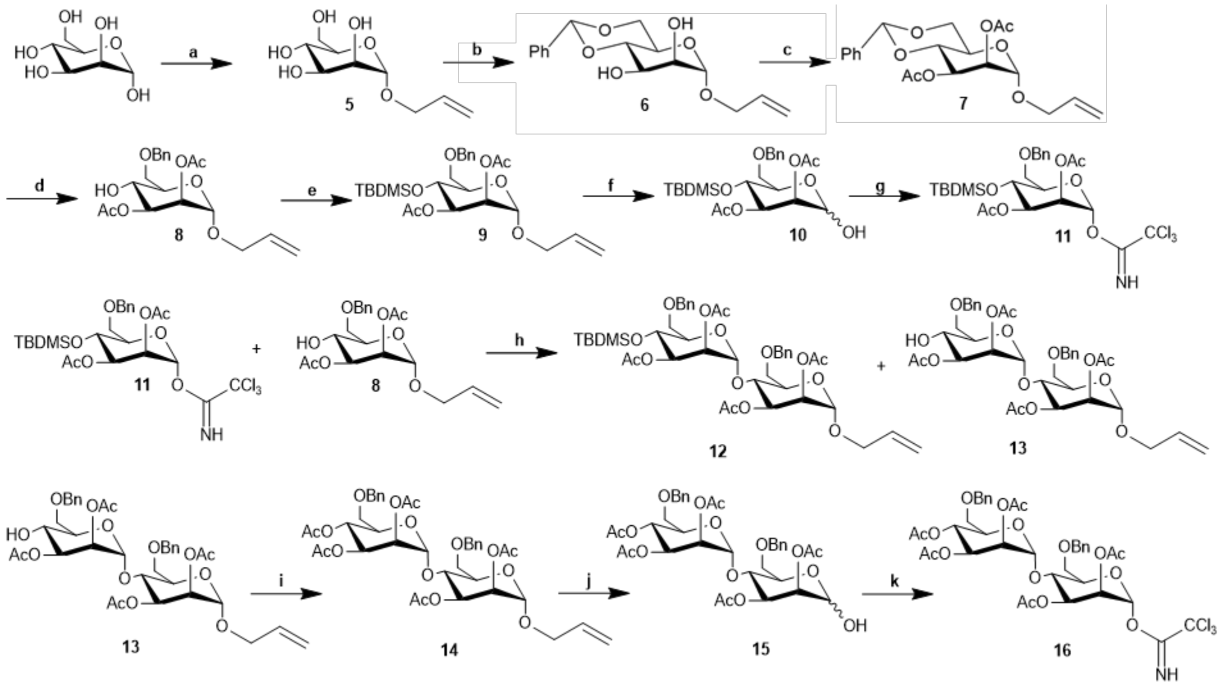

a) Allyl alcohol, reflux, 80%; b) $PhCH(OMe)_2$, THF, reflux, 75%; c) Ac_2O, DMAP, pyridine, 88%; d) $NaBH_3CN$, HCl, Et_2O, 0 °C, 88%; e) TBDMSOTf, DIPEA, CH_2Cl_2, 0 °C to rt, 55%; f) $PdCl_2$, MeOH, rt, 63%; g) CCl_3CN, DBU, CH_2Cl_2, 0 °C to rt, 65%; h) TMSOTf, CH_2Cl_2, 4Å MS, -20 °C, 80%; i) Ac_2O, DMAP, pyridine, 89%; j) $PdCl_2$, MeOH, rt, 89%; k) CCl_3CN, DBU, CH_2Cl_2, 0 °C to rt, 80%.

Scheme 2

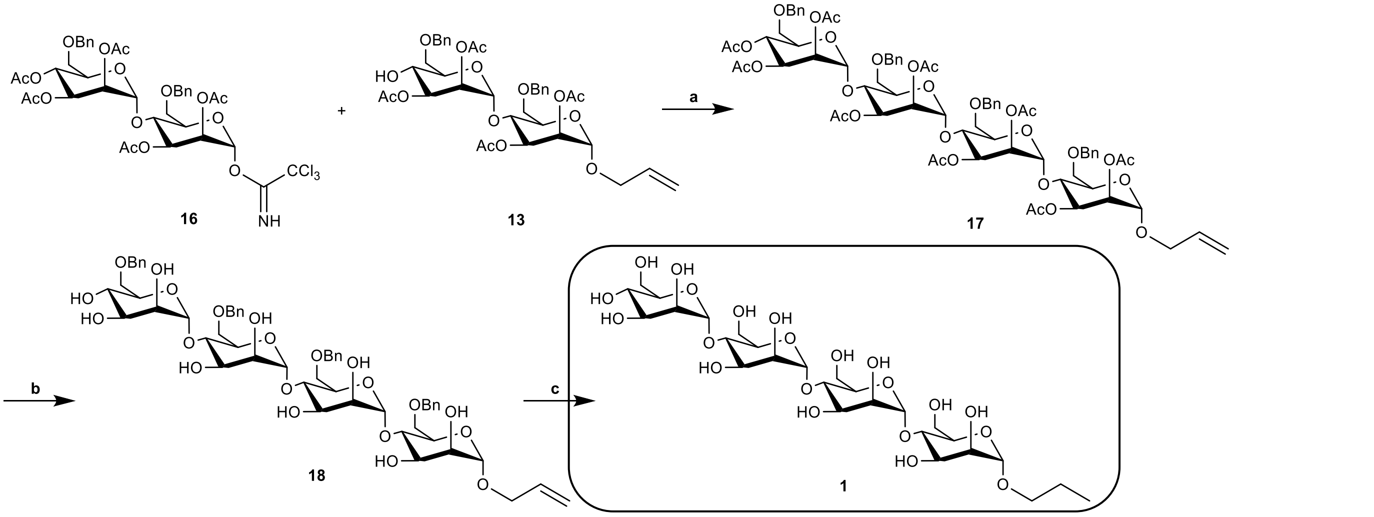

a) TMSOTf, CH_2Cl_2, 4Å MS, -20 °C, 80%; b) MeONa, MeOH, 0 °C to rt, quant.; c) H_2, Pd/C, AcOEt, 50 psi, 52%.

Scheme 3

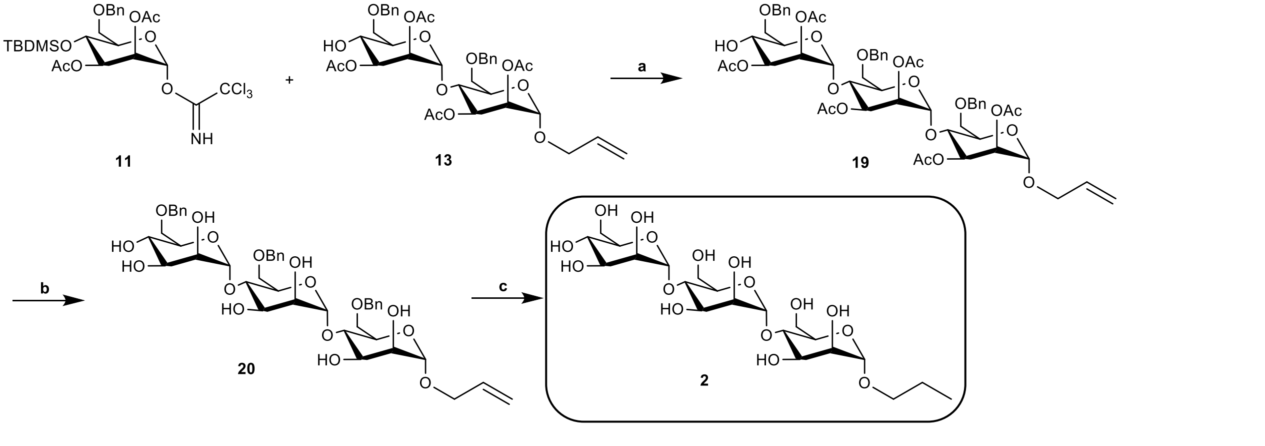

a) TMSOTf, CH_2Cl_2, 4Å MS, -20 °C, 67%; b) MeONa, MeOH, 0 °C to rt, 67%; c) H_2, Pd/C, AcOEt, 50 psi, quant.

Scheme 4

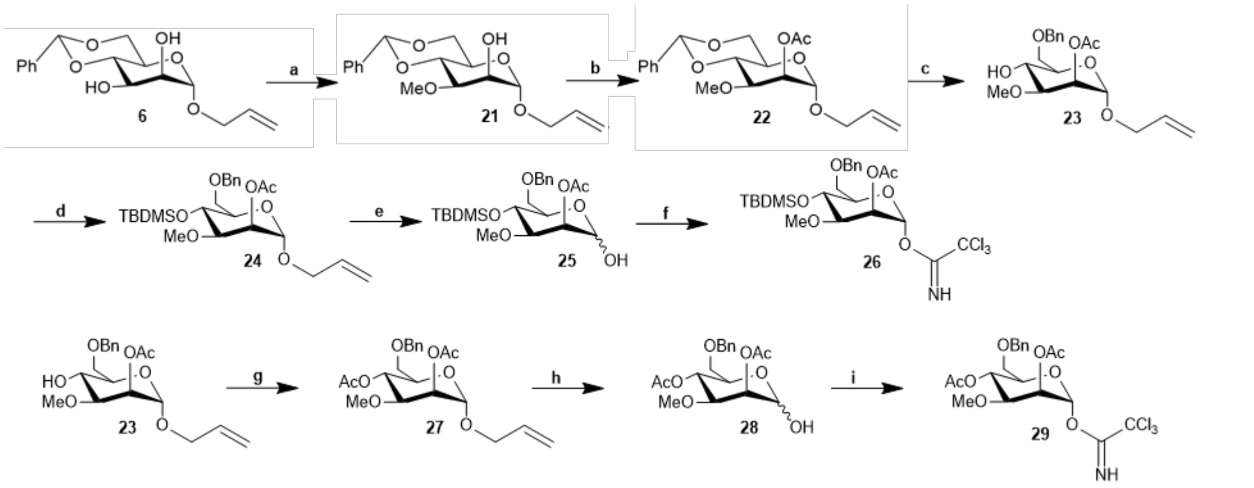

a) i. Bu_2SnO, MeOH, reflux; ii. MeI, DMF, 50 °C, 58%; b) Ac_2O, DMAP, pyridine, rt, 92%; c) $NaBH_3CN$, HCl, Et_2O, 0 °C, 78%; d) TBDMSOTf, DIPEA, CH_2Cl_2, 0 °C to rt, 64%; e) $PdCl_2$, MeOH, rt, 50%; f) CCl_3CN, DBU, CH_2Cl_2, 0 °C to rt, 57%; g) Ac_2O, DMAP, pyridine, rt, 83%; h) $PdCl_2$, MeOH, rt, 89%; i) CCl_3CN, DBU, CH_2Cl_2, 0 °C to rt, 70%.

Scheme 5

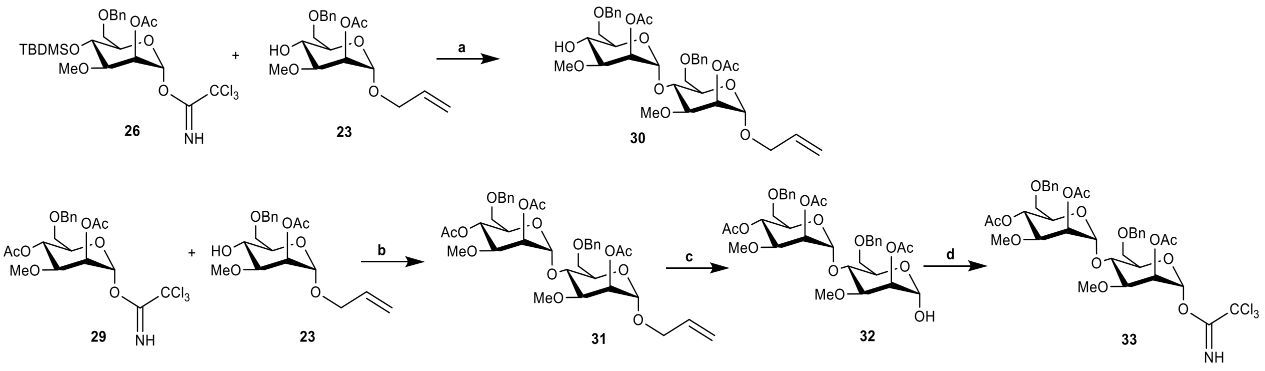

a) TMSOTf, CH_2Cl_2, 4Å MS, -20 °C, 48%; b) TMSOTf, CH_2Cl_2, 4Å MS, -20 °C, 65%; c) $PdCl_2$, MeOH, rt, 73%; d) CCl_3CN, DBU, CH_2Cl_2, 0 °C to rt, 68%.

Scheme 6

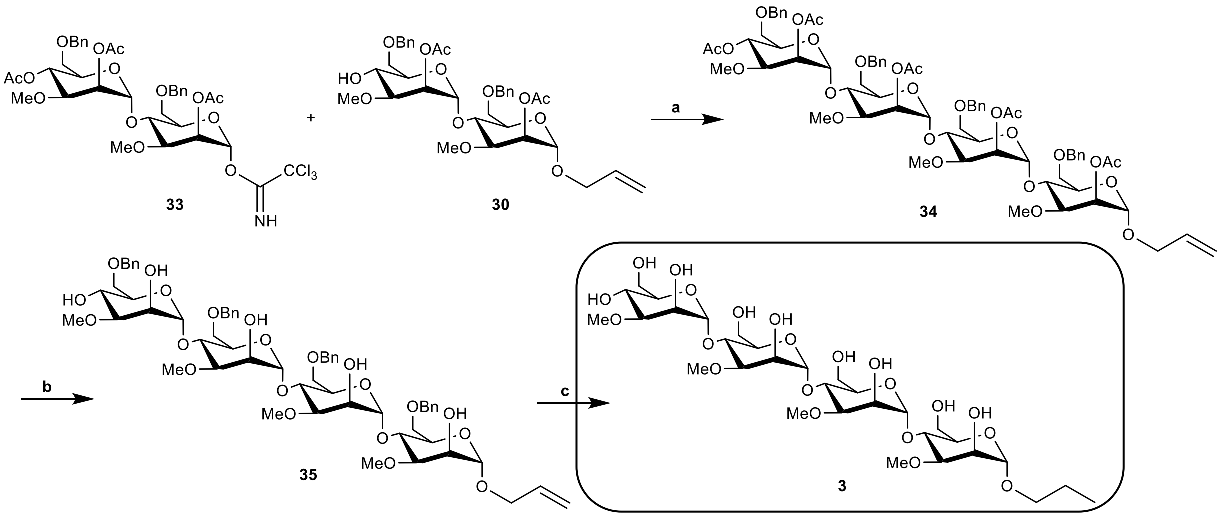

a) TMSOTf, CH_2Cl_2, 4Å MS, -20 °C, 86%; b) MeONa, MeOH, 0 °C to rt, 84%; c) H_2, Pd/C, AcOEt, 50 psi, quant.

Scheme 7

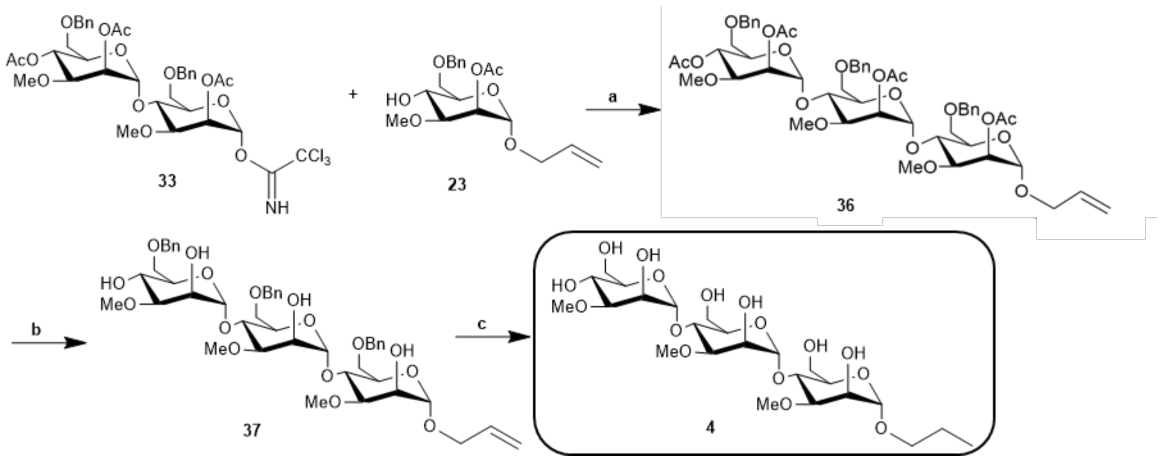

a) TMSOTf, CH_2Cl_2, 4Å MS, -20 °C, 85%; b) MeONa, MeOH, 0 °C to rt, 76%; c) H_2, Pd/C, AcOEt, 50 psi, quant.

NMR spectra of newly synthetized compounds

^{1}H-NMR (400 MHz) spectrum of allyl 2,3-di-*O*-acetyl-6-*O*-benzyl-α-D-mannopyranoside **8**.

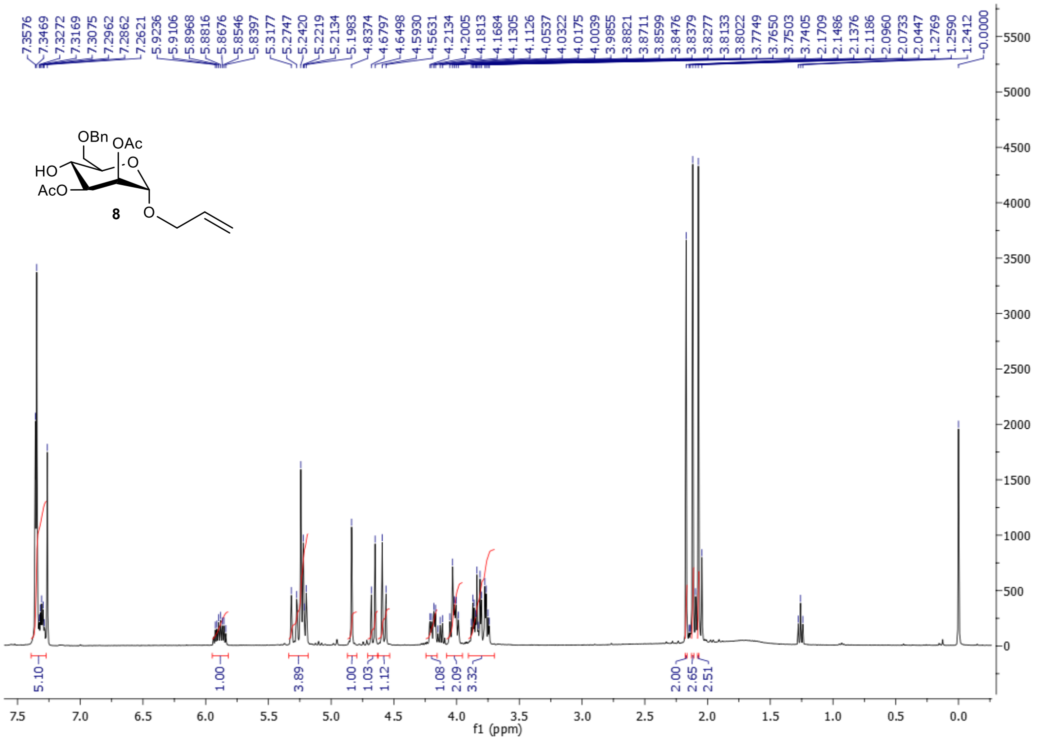

^{13}C-NMR (100.61 MHz) spectrum of allyl 2,3-di-*O*-acetyl-6-*O*-benzyl-α-D-mannopyranoside **8**.

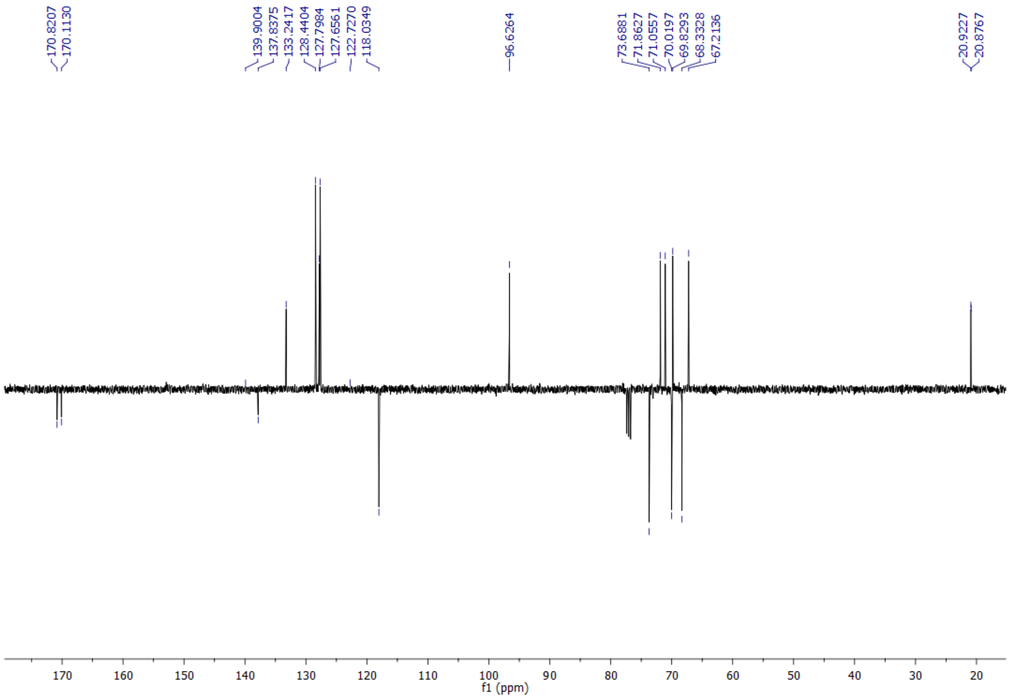

^{1}H-NMR (400 MHz) spectrum of allyl 2,3-di-*O*-acetyl-6-*O*-benzyl-4-*O*-tert-butyldimethylsilyl-α-D-mannopyranoside **9**.

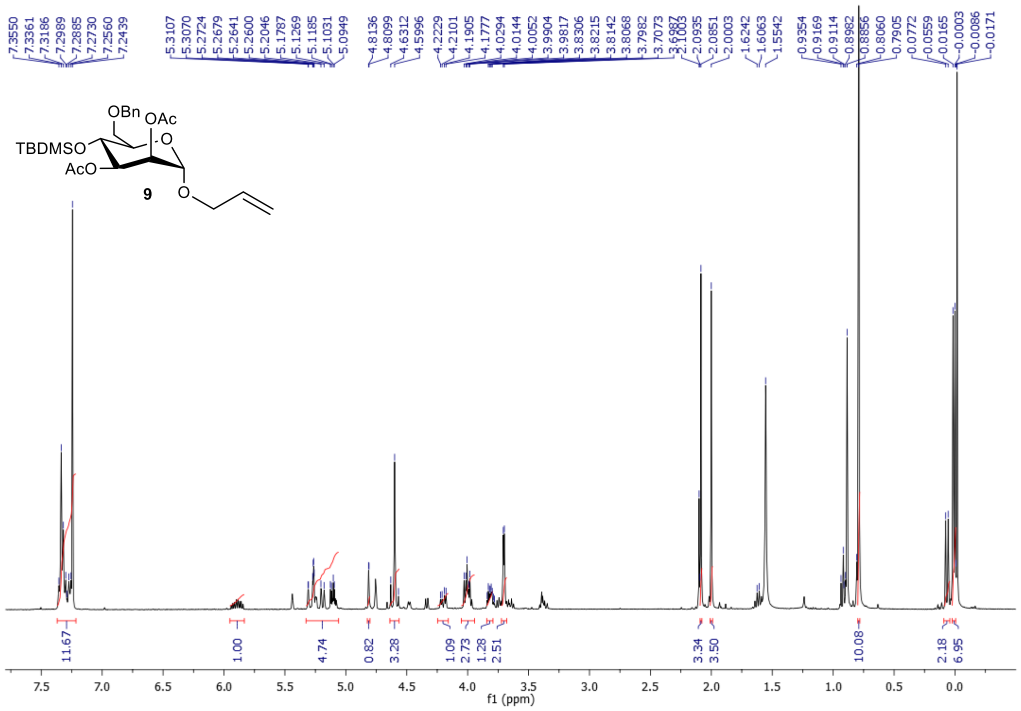

^{13}C-NMR (100.61 MHz) spectrum of allyl 2,3-di-*O*-acetyl-6-*O*-benzyl-4-*O*-tert-butyldimethylsilyl-α-D-mannopyranoside **9**.

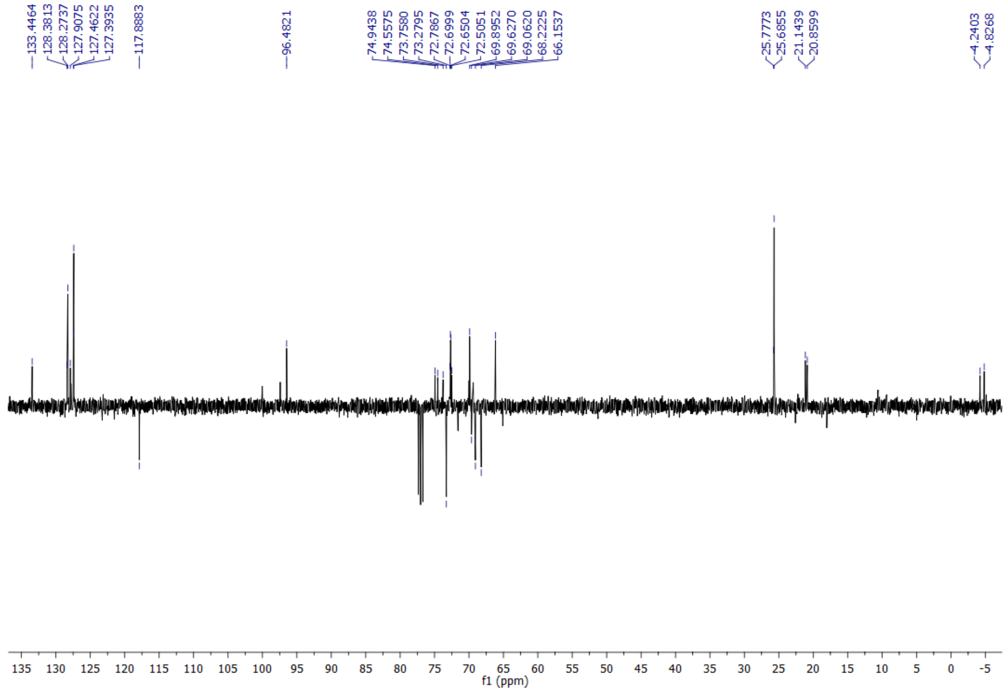

^{1}H-NMR (400 MHz) spectrum of 2,3-di-*O*-acetyl-6-*O*-benzyl-4-*O*-*tert*-butyldimethylsilyl-(α/β)-D-mannopyranoside **10**.

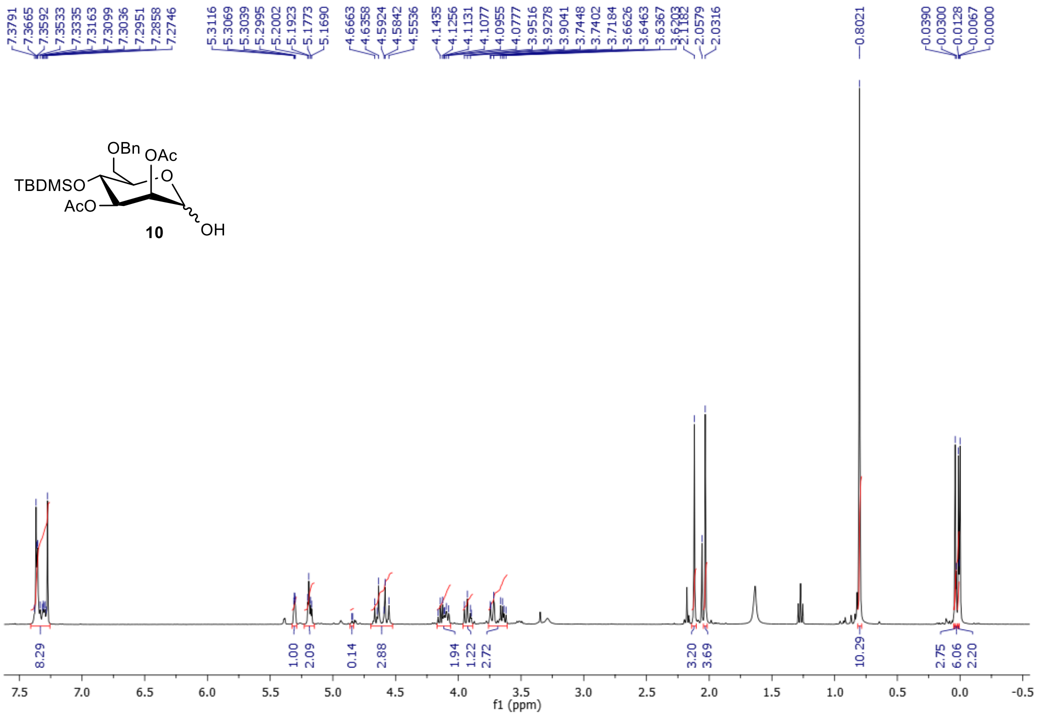

^{13}C-NMR (100.61 MHz) spectrum of 2,3-di-*O*-acetyl-6-*O*-benzyl-4-*O*-*tert*-butyldimethylsilyl-(α/β)-D-mannopyranoside **10**.

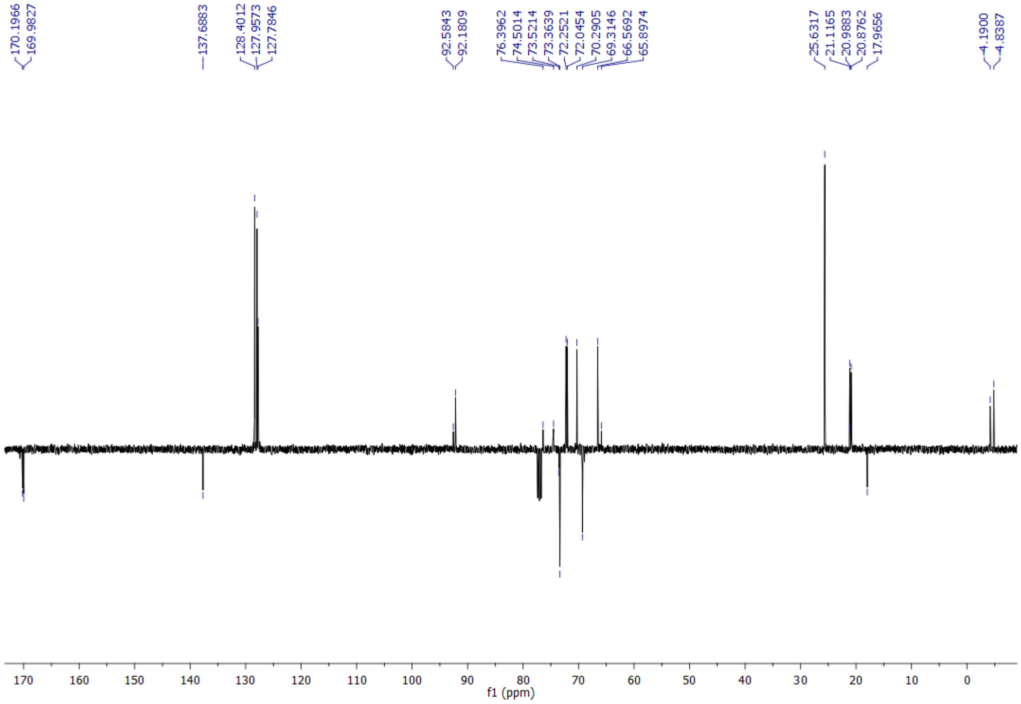

^{1}H-NMR (400 MHz) spectrum of 2,3-di-*O*-acetyl-6-*O*-benzyl-4-*O*-tert-butyldimethylsilyl-α-D-mannopyranosyl trichloroacetimidate **11**.

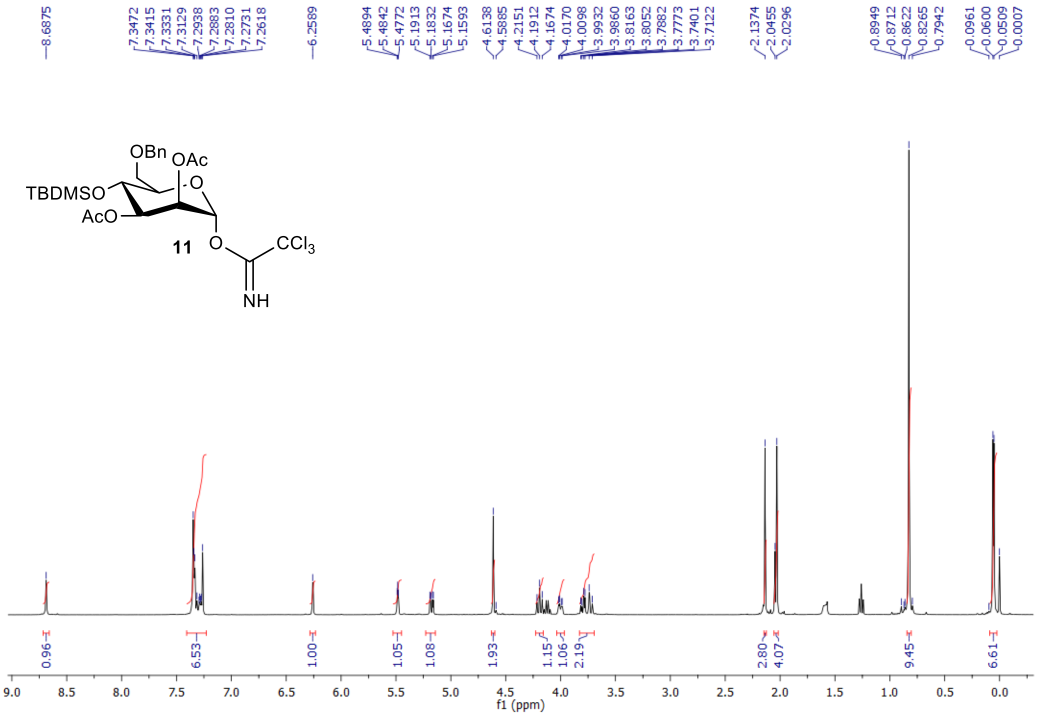

^{13}C-NMR (100.61 MHz) spectrum of 2,3-di-*O*-acetyl-6-*O*-benzyl-4-*O*-tert-butyldimethylsilyl-α-D-mannopyranosyl trichloroacetimidate **11**.

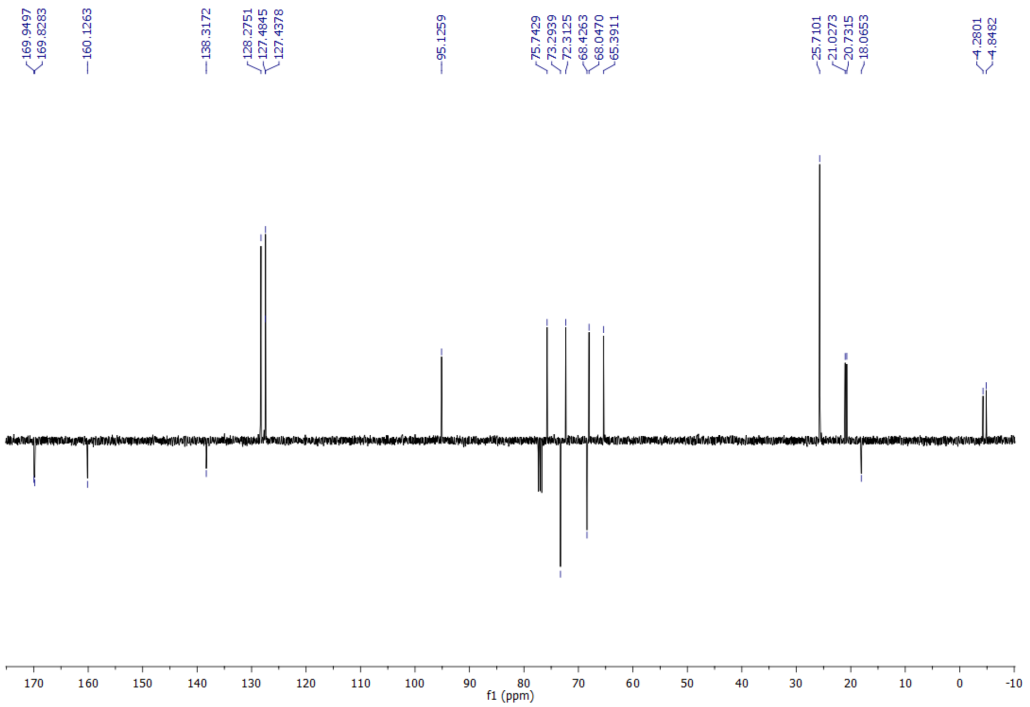

^{1}H-NMR (400 MHz) spectrum of allyl 2,3-di-*O*-acetyl-6-*O*-benzyl-4-*O*-*tert*-butyldimethylsilyl-α-D-mannopyranosyl-(1→4)-2,3-di-*O*-acetyl-6-*O*-benzyl-α-D-mannopyranoside **12**.

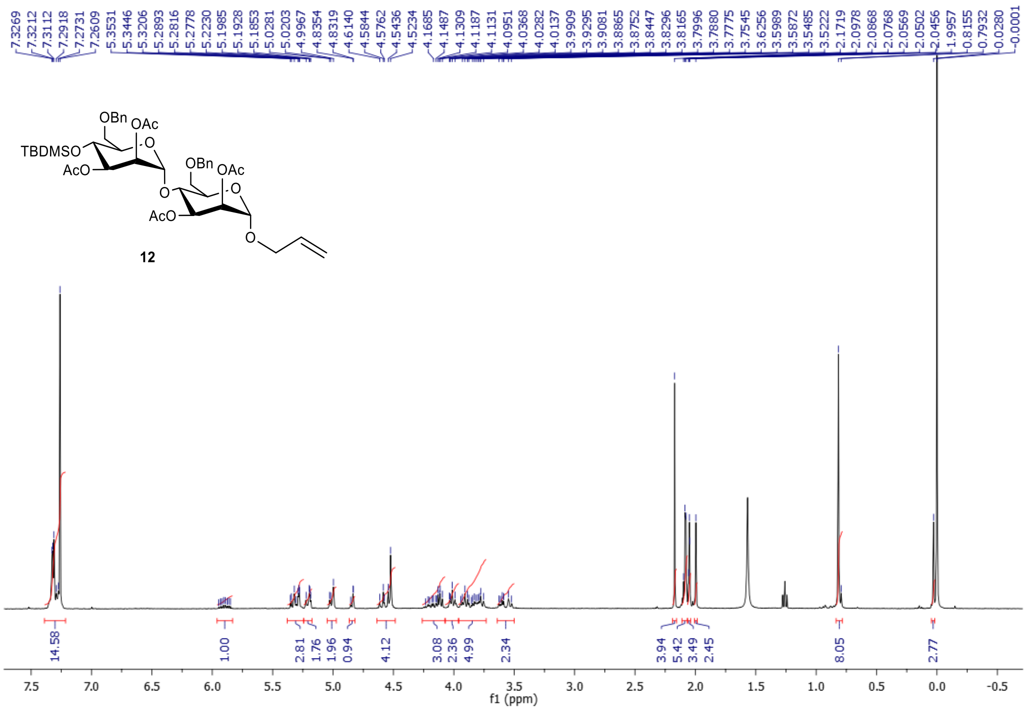

^{13}C-NMR (100.61 MHz) spectrum of allyl 2,3-di-*O*-acetyl-6-*O*-benzyl-4-*O*-*tert*-butyldimethylsilyl-α-D-mannopyranosyl-(1→4)-2,3-di-*O*-acetyl-6-*O*-benzyl-α-D-mannopyranoside **12**.

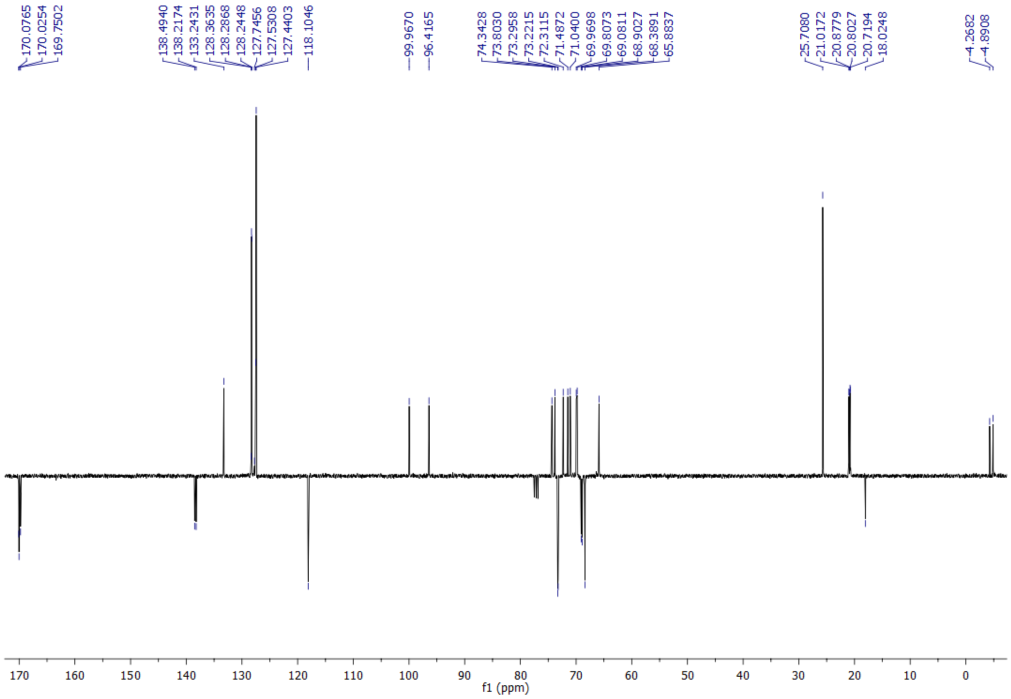

^{1}H-NMR (400 MHz) spectrum of allyl 2,3-di-*O*-acetyl-6-*O*-benzyl-α-D-mannopyranosyl-(1→4)-2,3-di-*O*-acetyl-6-*O*-benzyl-α-D-mannopyranoside **13**.

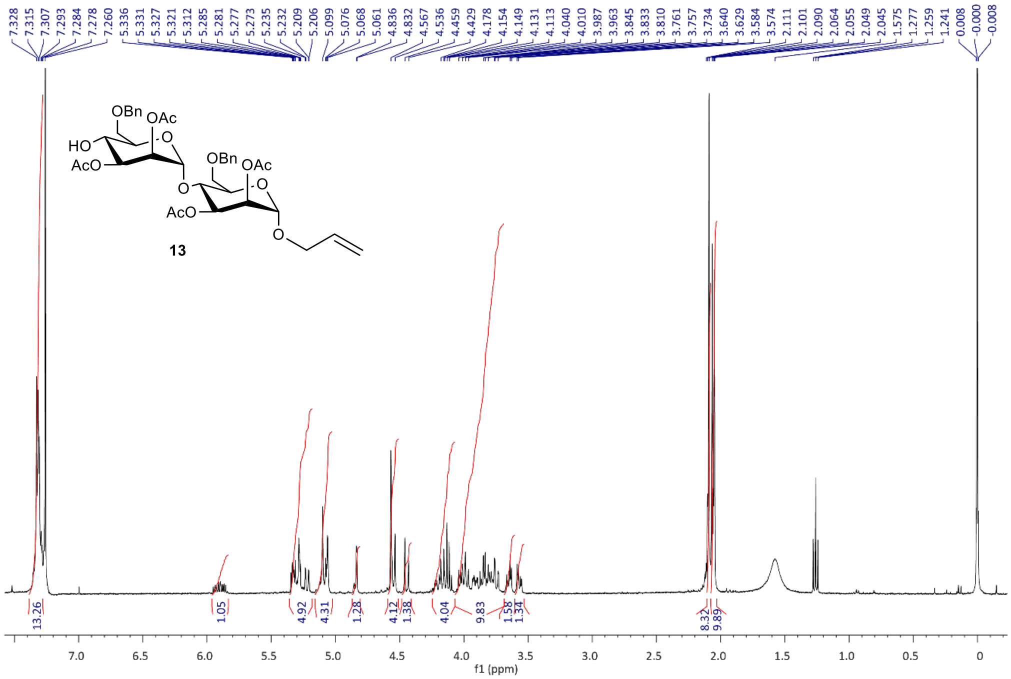

^{13}C-NMR (100.61 MHz) spectrum of allyl 2,3-di-*O*-acetyl-6-*O*-benzyl-α-D-mannopyranosyl-(1→4)-2,3-di-*O*-acetyl-6-*O*-benzyl-α-D-mannopyranoside **13**.

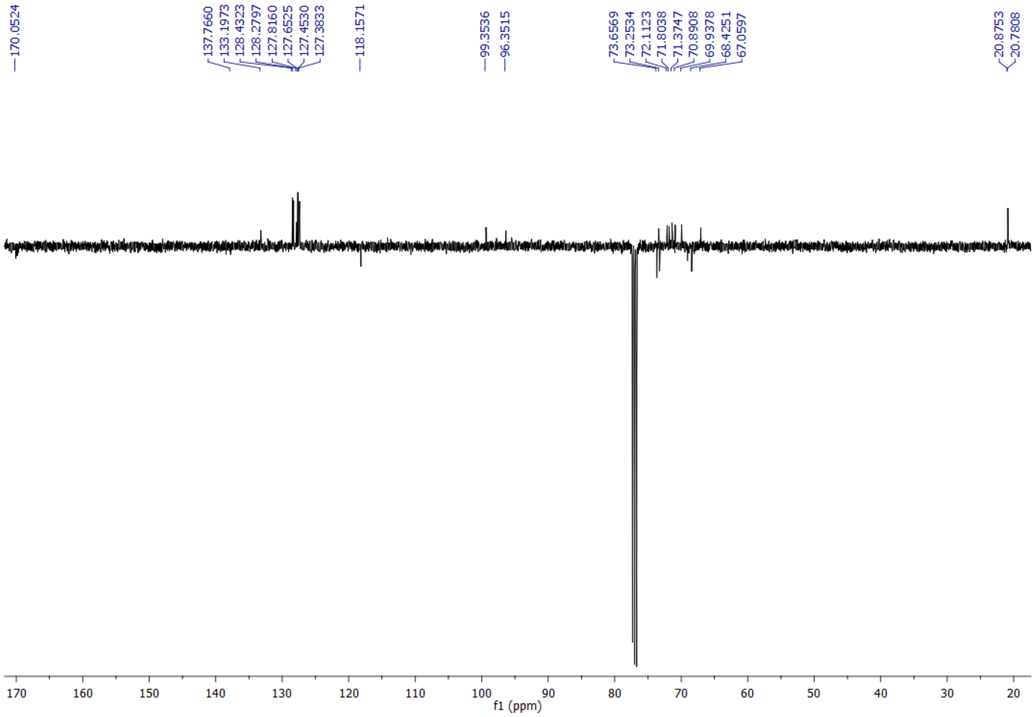

^{1}H-NMR (400 MHz) spectrum of allyl 2,3,4-tri-*O*-acetyl-6-*O*-benzyl-α-D-mannopyranosyl-(1→4)-2,3-di-*O*-acetyl-6-*O*-benzyl-α-D-mannopyranoside **14**.

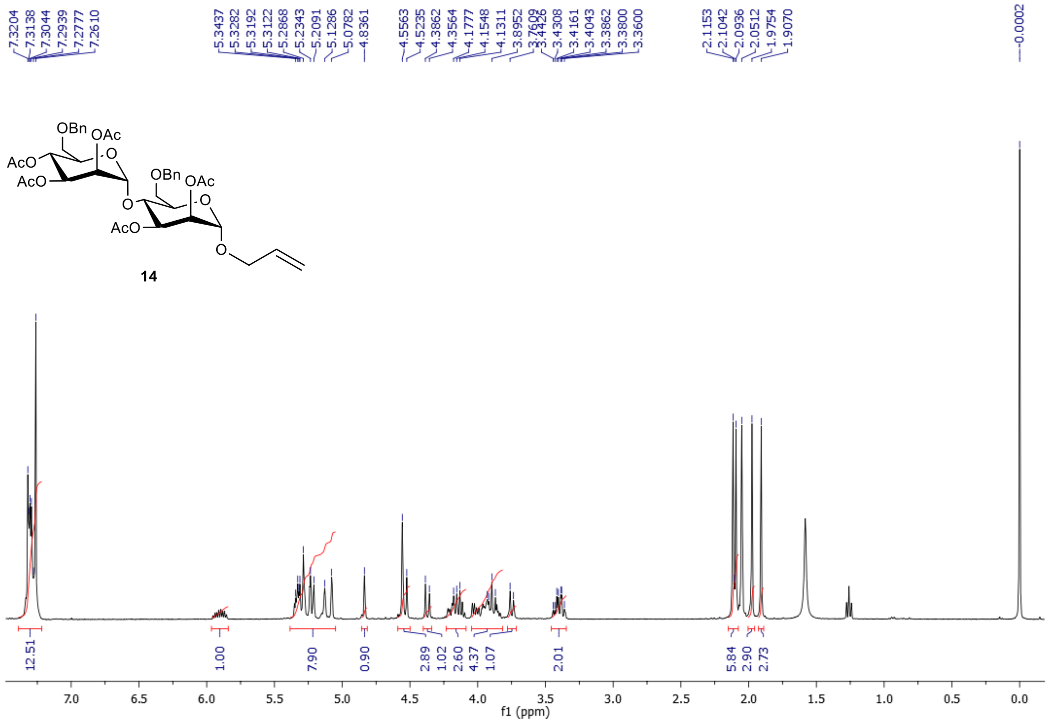

^{13}C-NMR (100.61 MHz) spectrum of allyl 2,3,4-tri-*O*-acetyl-6-*O*-benzyl-α-D-mannopyranosyl-(1→4)-2,3-di-*O*-acetyl-6-*O*-benzyl-α-D-mannopyranoside **14**.

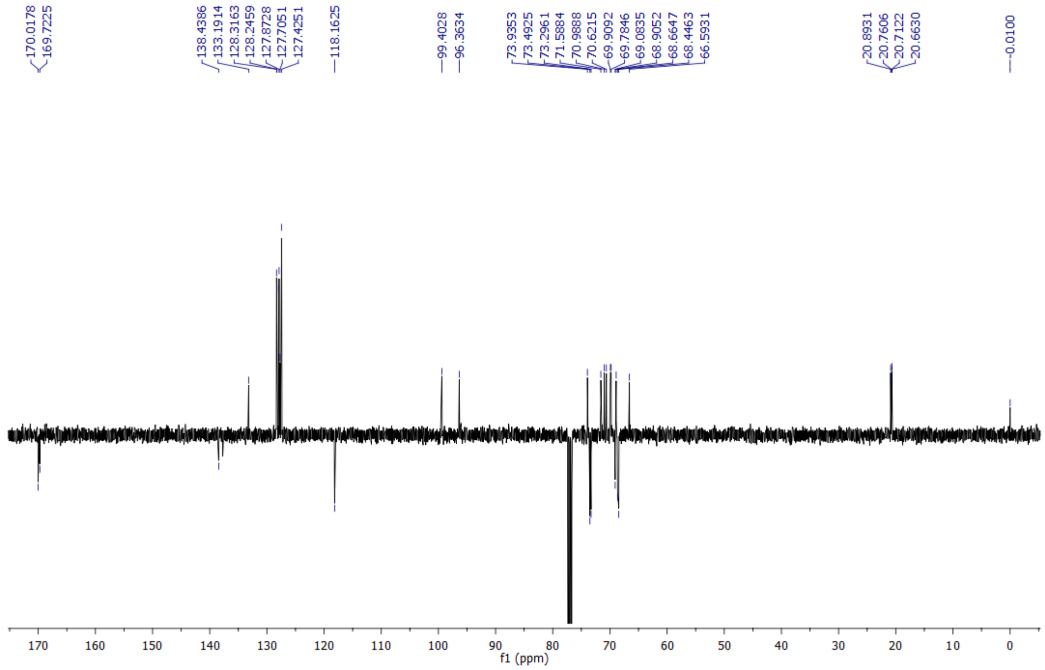

^{1}H-NMR (400 MHz) spectrum of 2,3,4-tri-*O*-acetyl-6-*O*-benzyl-α-D-mannopyranosyl-(1→4)-2,3-di-*O*-acetyl-6-*O*-benzyl-(α/β)-D-mannopyranoside **15**.

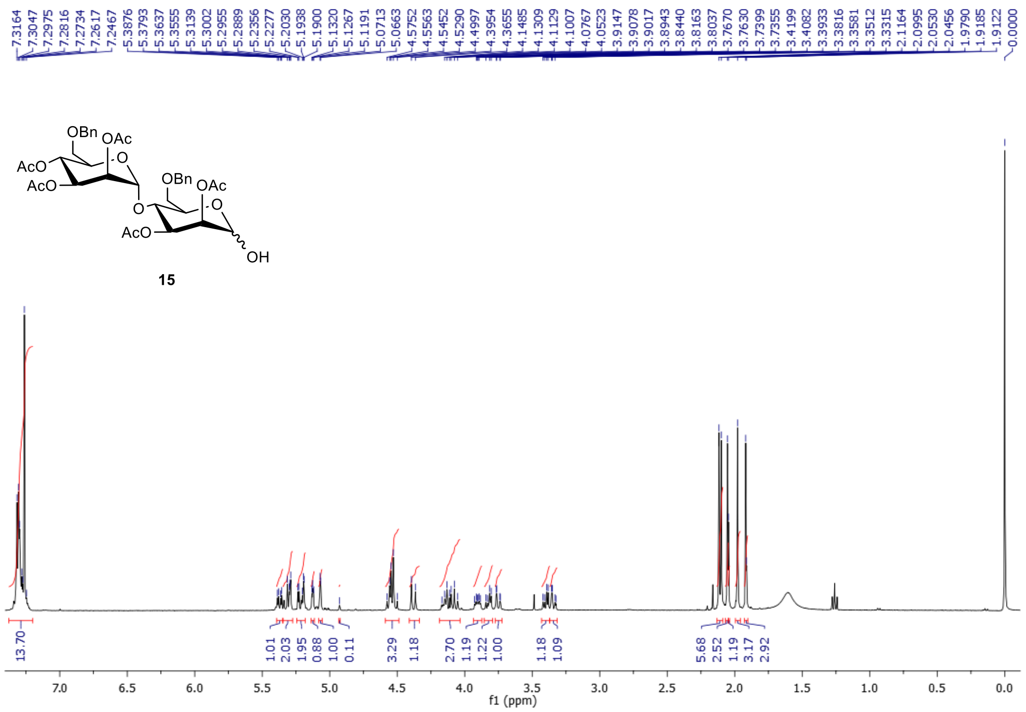

^{13}C-NMR (100.61 MHz) spectrum of 2,3,4-tri-*O*-acetyl-6-*O*-benzyl-α-D-mannopyranosyl-(1→4)-2,3-di-*O*-acetyl-6-*O*-benzyl-(α/β)-D-mannopyranoside **15**.

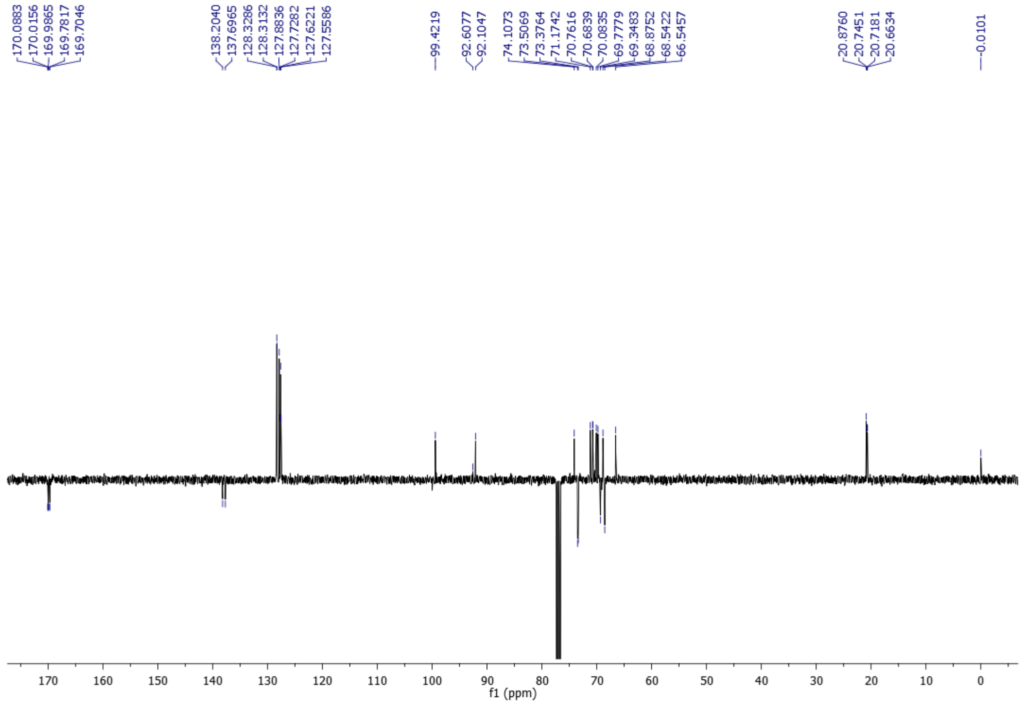

^{1}H-NMR (400 MHz) spectrum of 2,3,4-tri-*O*-acetyl-6-*O*-benzyl-α-D-mannopyranosyl-(1→4)-2,3-di-*O*-acetyl-6-*O*-benzyl-α-D-mannopyranosyl trichloroacetimidate **16**.

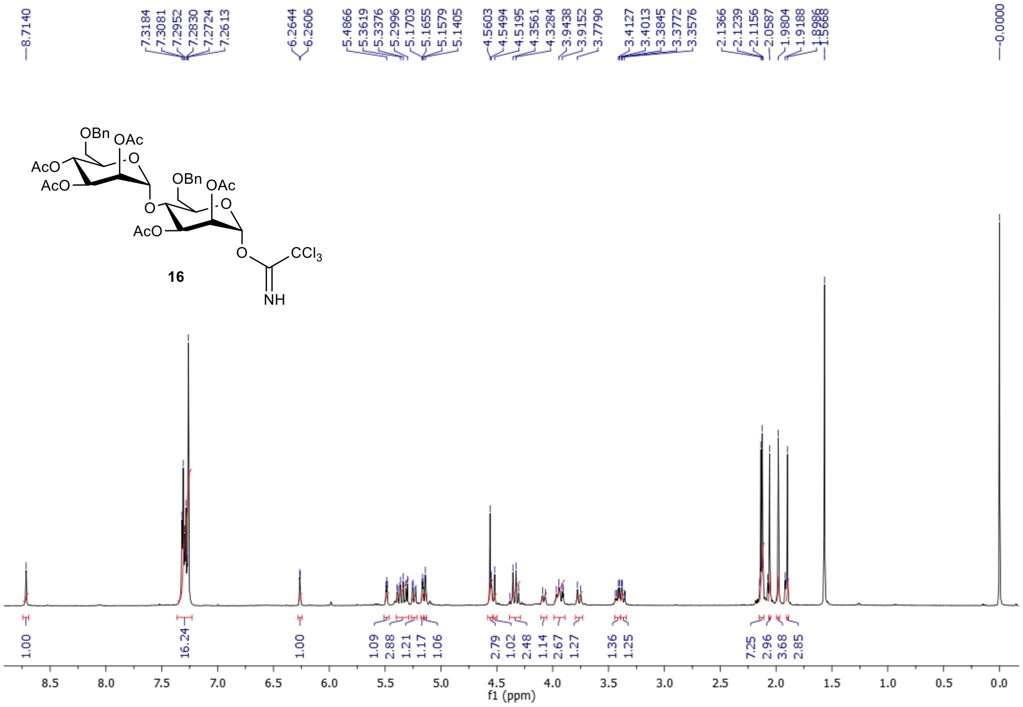

^{13}C-NMR (100.61 MHz) spectrum of 2,3,4-tri-*O*-acetyl-6-*O*-benzyl-α-D-mannopyranosyl-(1→4)-2,3-di-*O*-acetyl-6-*O*-benzyl-α-D-mannopyranosyl trichloroacetimidate **16**.

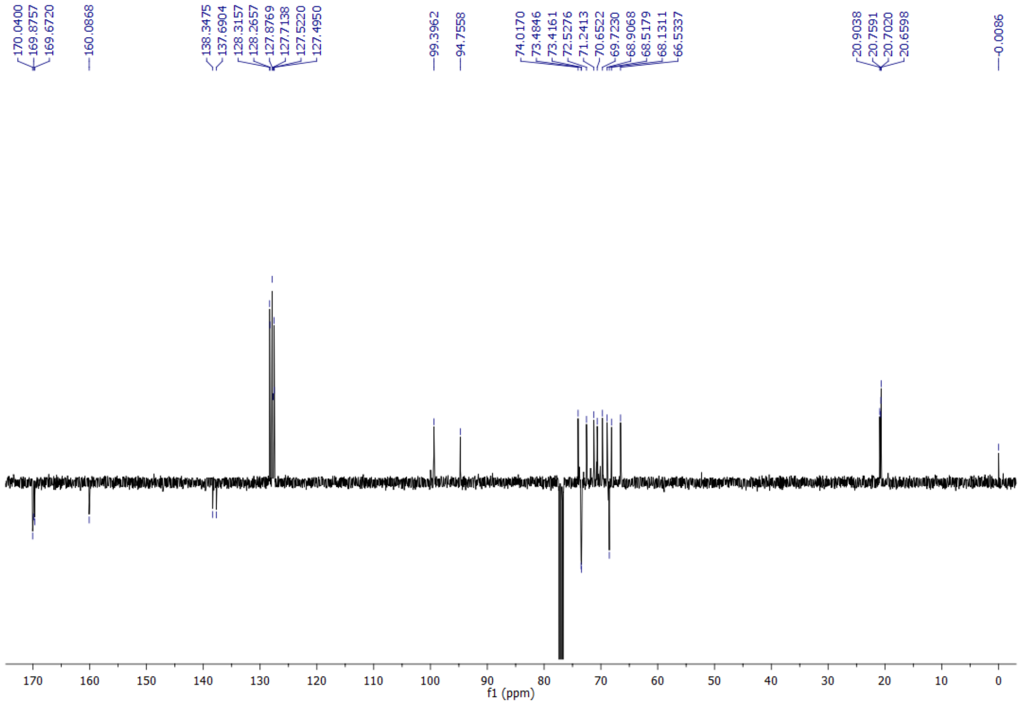

^{1}H-NMR (400 MHz) spectrum of allyl 2,3,4-tri-*O*-acetyl-6-*O*-benzyl-α-D-mannopyranosyl-(1→4)-2,3-di-*O*-acetyl-6-*O*-benzyl-α-D-mannopyranosyl-(1→4)-2,3-di-*O*-acetyl-6-*O*-benzyl-α-D-mannopyranosyl-(1→4)-2,3-di-*O*-acetyl-6-*O*-benzyl-α-D-mannopyranoside **17**.

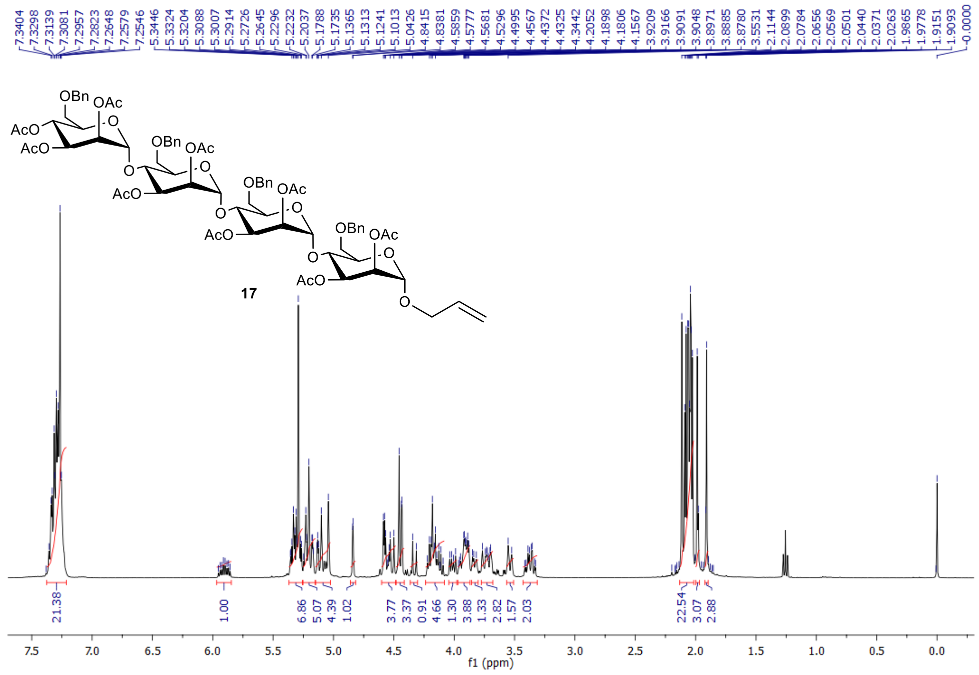

^{13}C-NMR (100.61 MHz) spectrum of allyl 2,3,4-tri-*O*-acetyl-6-*O*-benzyl-α-D-mannopyranosyl-(1→4)-2,3-di-*O*-acetyl-6-*O*-benzyl-α-D-mannopyranosyl-(1→4)-2,3-di-*O*-acetyl-6-*O*-benzyl-α-D-mannopyranosyl-(1→4)-2,3-di-*O*-acetyl-6-*O*-benzyl-α-D-mannopyranoside **17**.

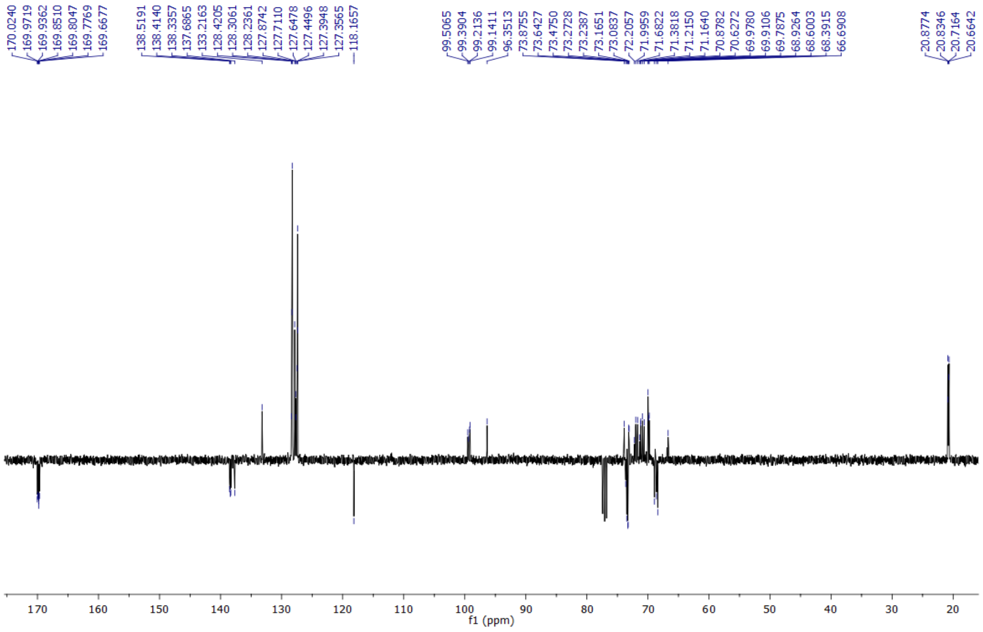

¹H-NMR (400 MHz) spectrum of allyl 6-*O*-benzyl-α-D-mannopyranosyl-(1→4)-6-*O*-benzyl-α-D-mannopyranosyl-(1→4)-6-*O*-benzyl-α-D-mannopyranosyl-(1→4)-6-*O*-benzyl-α-D-mannopyranoside **18**.

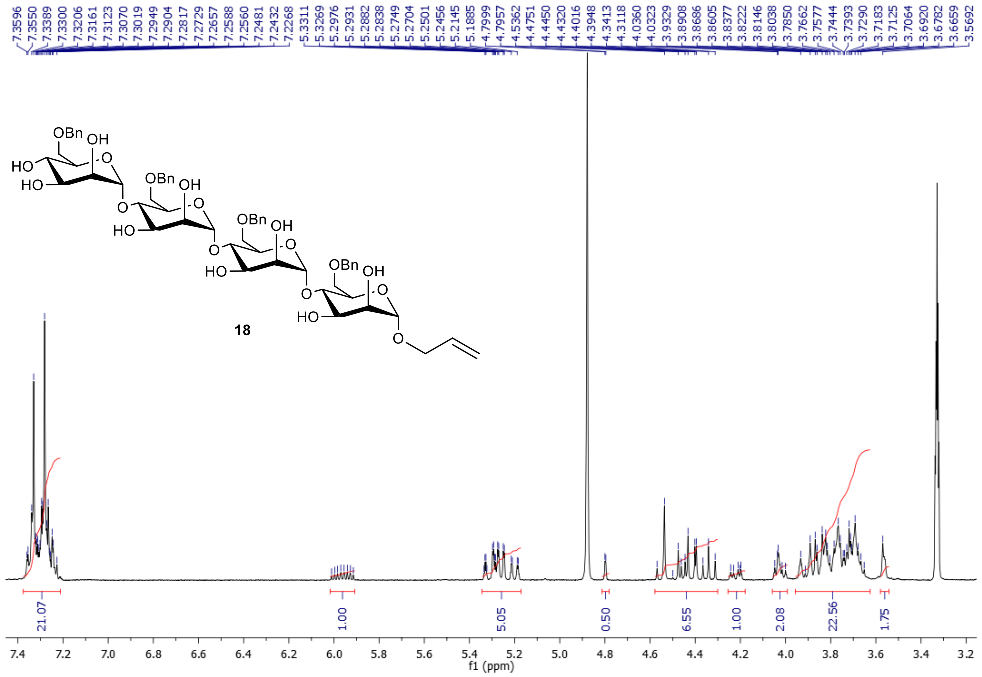

¹³C-NMR (100.61 MHz) spectrum of allyl 6-*O*-benzyl-α-D-mannopyranosyl-(1→4)-6-*O*-benzyl-α-D-mannopyranosyl-(1→4)-6-*O*-benzyl-α-D-mannopyranosyl-(1→4)-6-*O*-benzyl-α-D-mannopyranoside **18**.

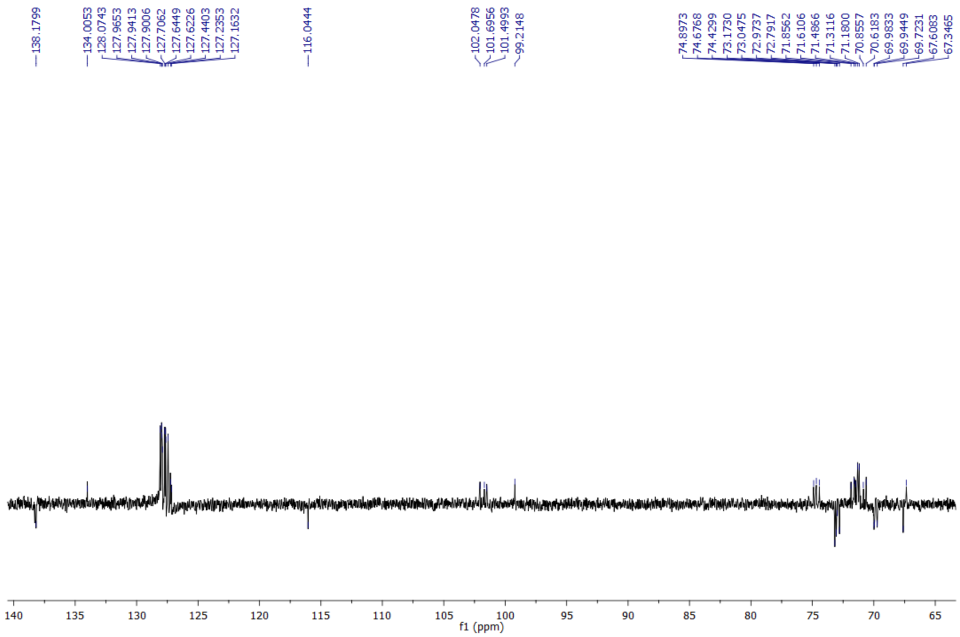

^{1}H-NMR (400 MHz) spectrum of propyl α-D-mannopyranosyl-(1→4)-α-D-mannopyranosyl-(1→4)-α-D-mannopyranosyl-(1→4)-α-D-mannopyranoside **1**.

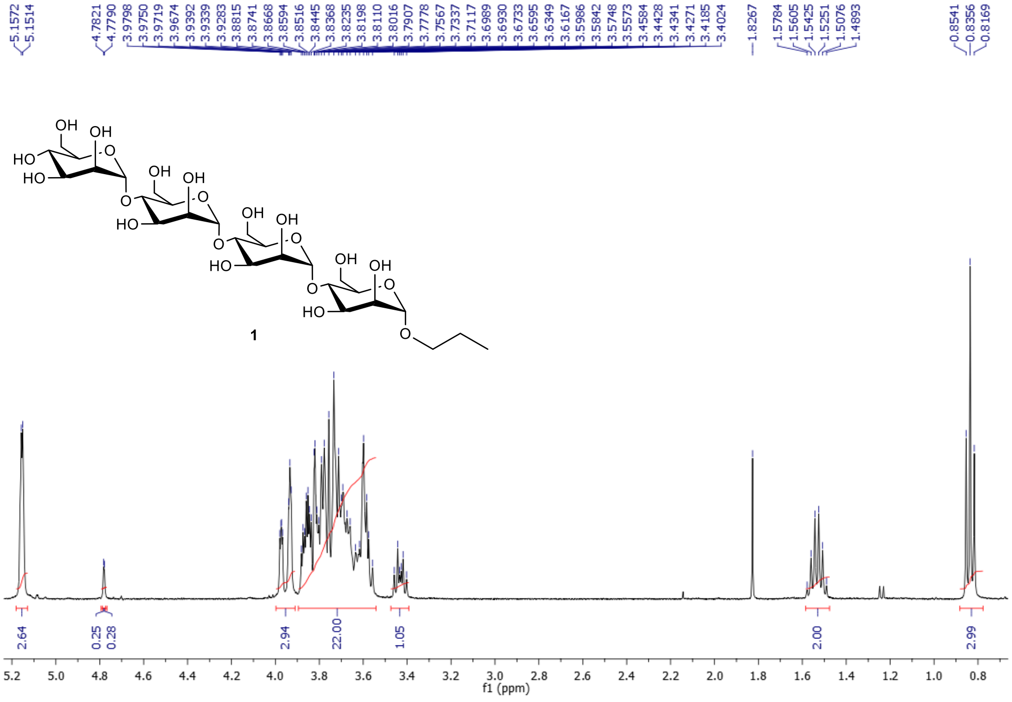

^{13}C-NMR (100.61 MHz) spectrum of propyl α-D-mannopyranosyl-(1→4)-α-D-mannopyranosyl-(1→4)-α-D-mannopyranosyl-(1→4)-α-D-mannopyranoside **1**.

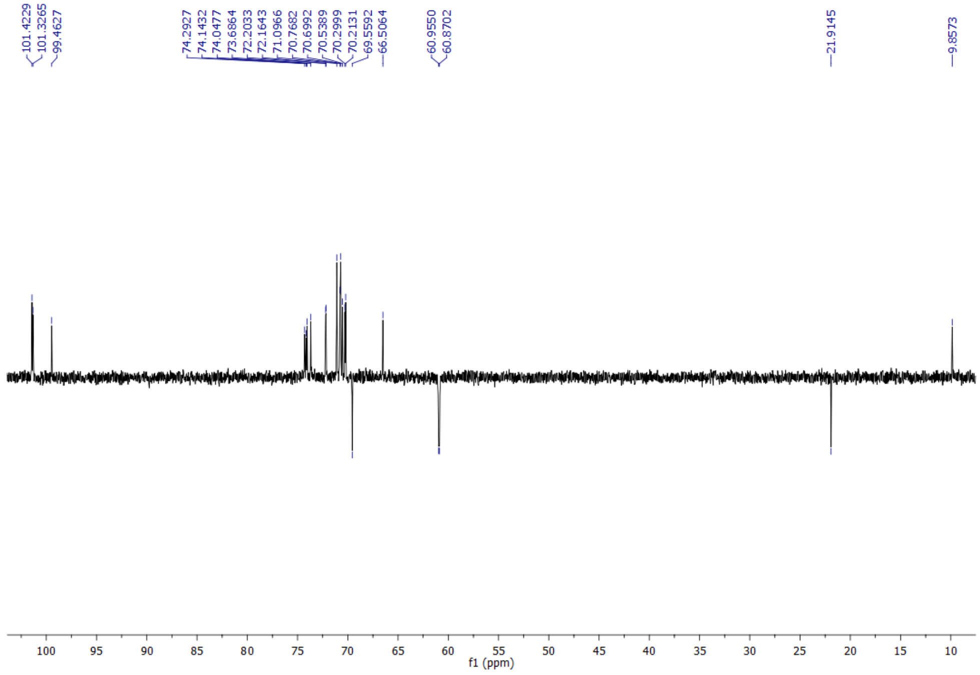

^{1}H-NMR (400 MHz) spectrum of allyl 2,3,4-tri-*O*-acetyl-6-*O*-benzyl-α-D-mannopyranosyl-(1→4)-2,3-di-*O*-acetyl-6-*O*-benzyl-α-D-mannopyranosyl-(1→4)-2,3-di-*O*-acetyl-6-*O*-benzyl-α-D-mannopyranose **19**.

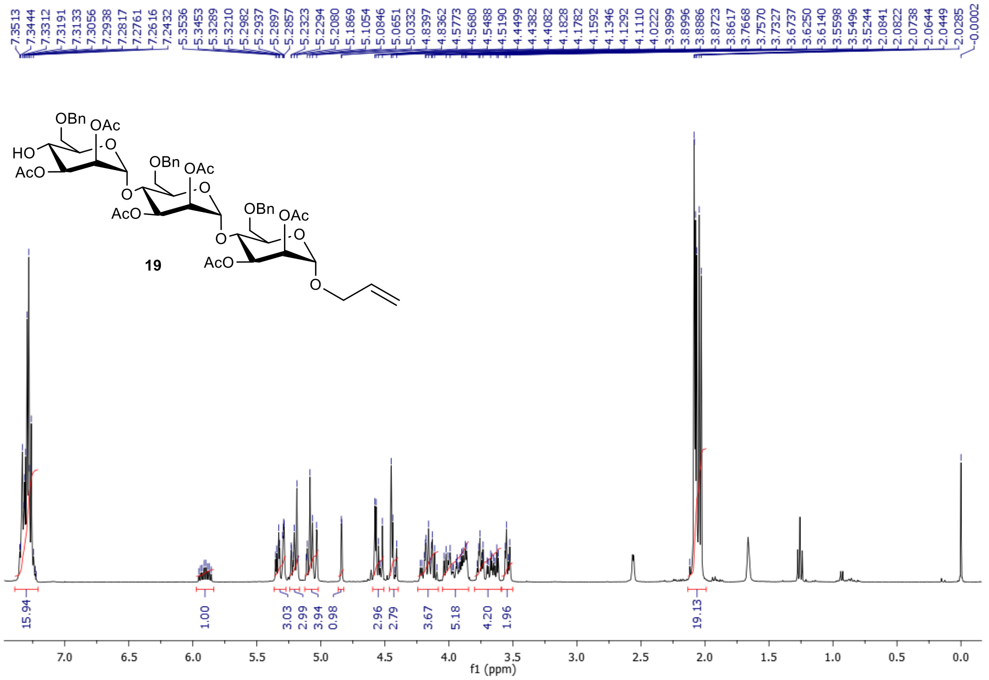

^{13}C-NMR (100.61 MHz) spectrum of allyl 2,3,4-tri-*O*-acetyl-6-*O*-benzyl-α-D-mannopyranosyl-(1→4)-2,3-di-*O*-acetyl-6-*O*-benzyl-α-D-mannopyranosyl-(1→4)-2,3-di-*O*-acetyl-6-*O*-benzyl-α-D-mannopyranose **19**.

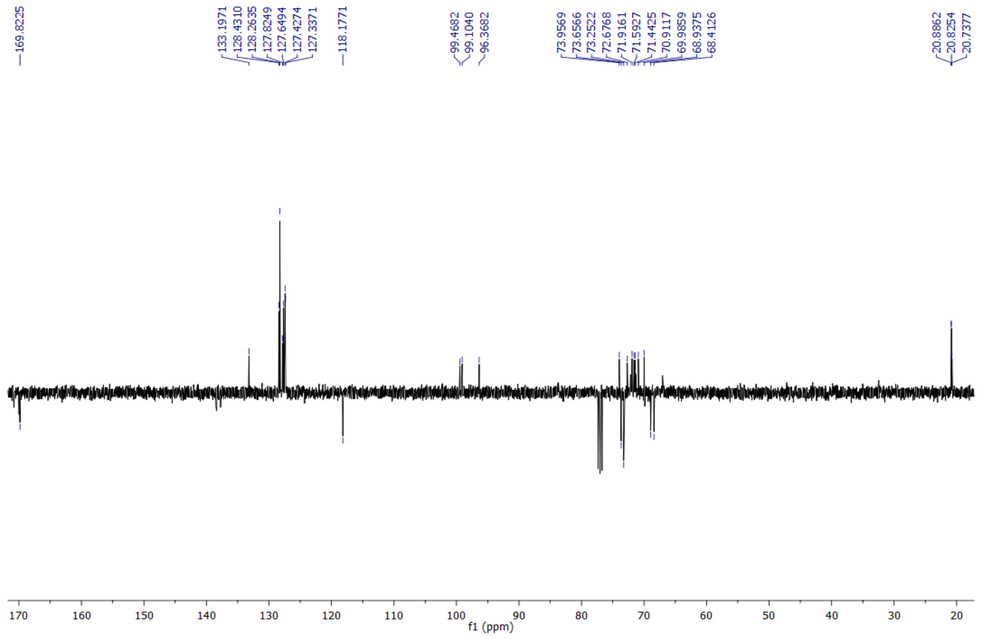

^{1}H-NMR (400 MHz) spectrum of allyl 6-*O*-benzyl-α-D-mannopyranosyl-(1→4)-6-*O*-benzyl-α-D-mannopyranosyl-(1→4)-6-*O*-benzyl-α-D-mannopyranoside **20**.

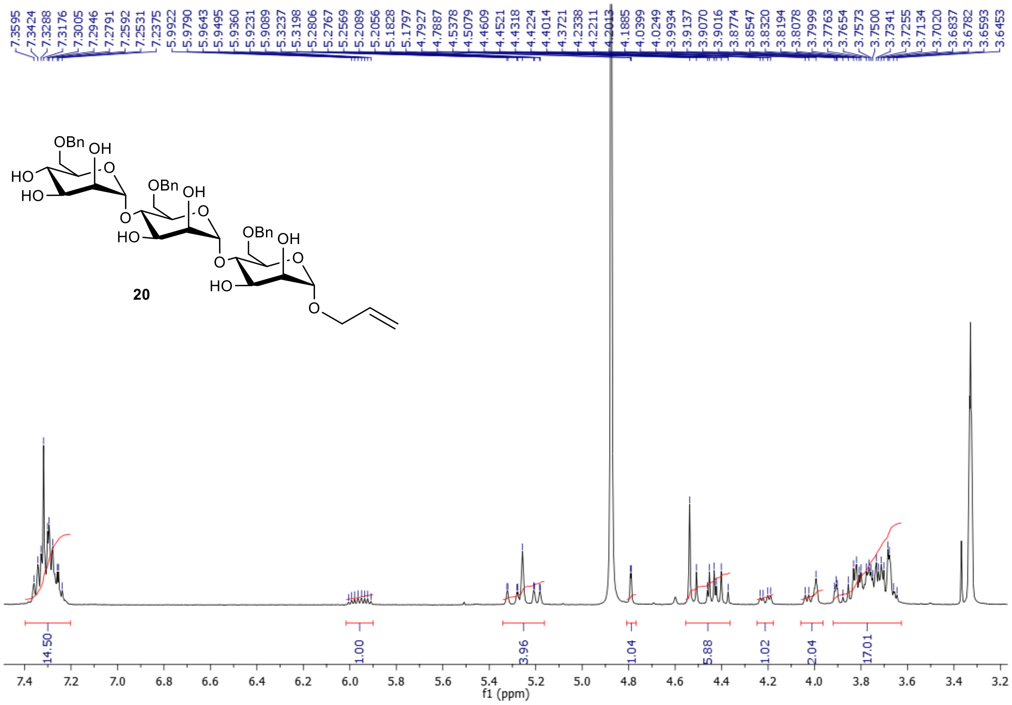

^{13}C-NMR (100.61 MHz) spectrum of allyl 6-*O*-benzyl-α-D-mannopyranosyl-(1→4)-6-*O*-benzyl-α-D-mannopyranosyl-(1→4)-6-*O*-benzyl-α-D-mannopyranoside **20**.

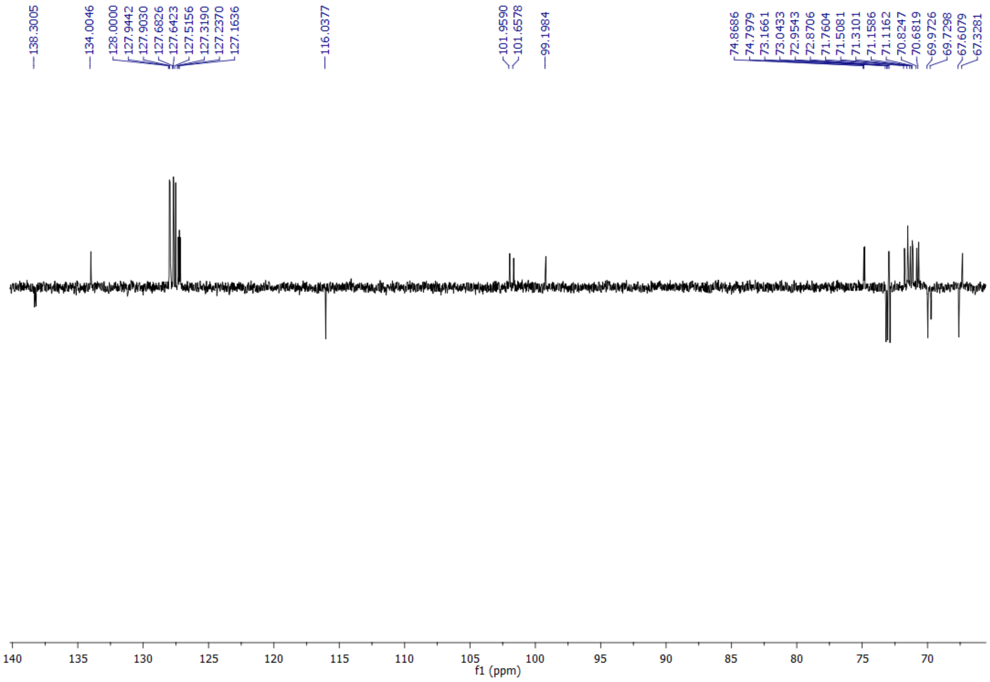

^{1}H-NMR (400 MHz) spectrum of propyl-α-D-mannopyranosyl-(1→4)-α-D-mannopyranosyl-(1→4)-α-D-mannopyranoside **2**.

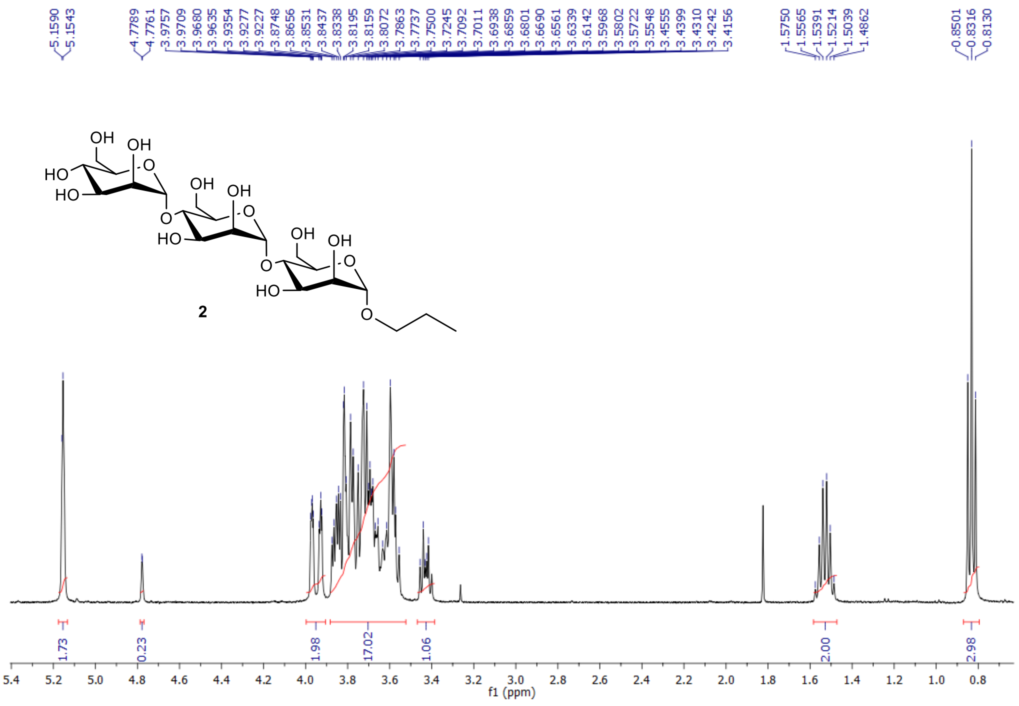

^{13}C-NMR (100.61 MHz) spectrum of propyl-α-D-mannopyranosyl-(1→4)-α-D-mannopyranosyl-(1→4)-α-D-mannopyranoside **2**.

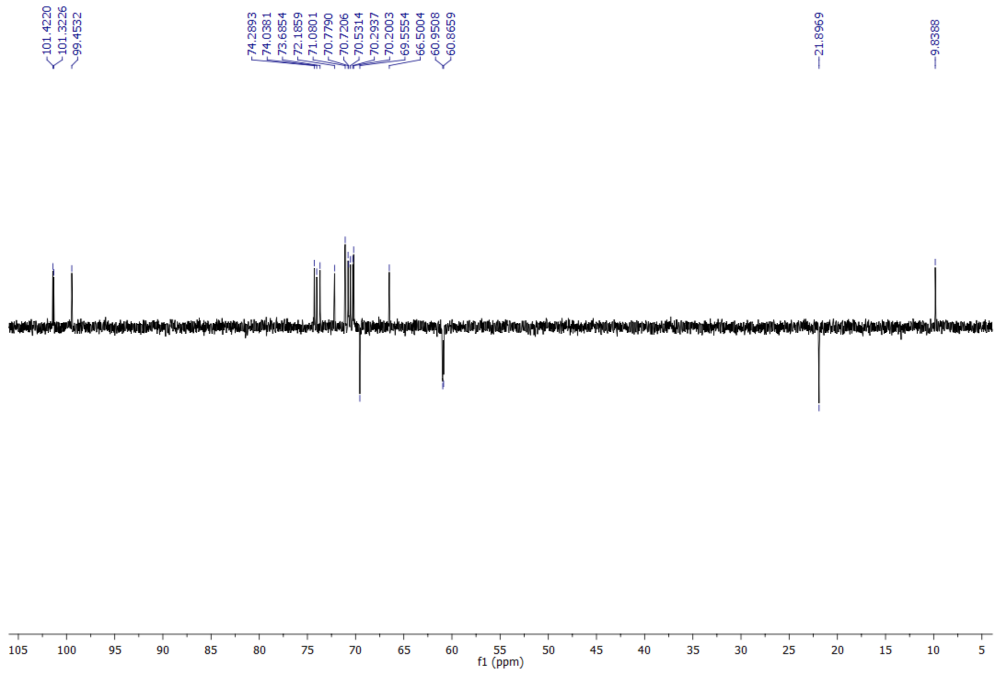

^{1}H-NMR (400 MHz) spectrum of allyl 2-*O*-acetyl-6-*O*-benzyl-4-*O*-*tert*-butyldimethylsilyl-3-*O*-methyl-α-D-mannopyranoside **24**.

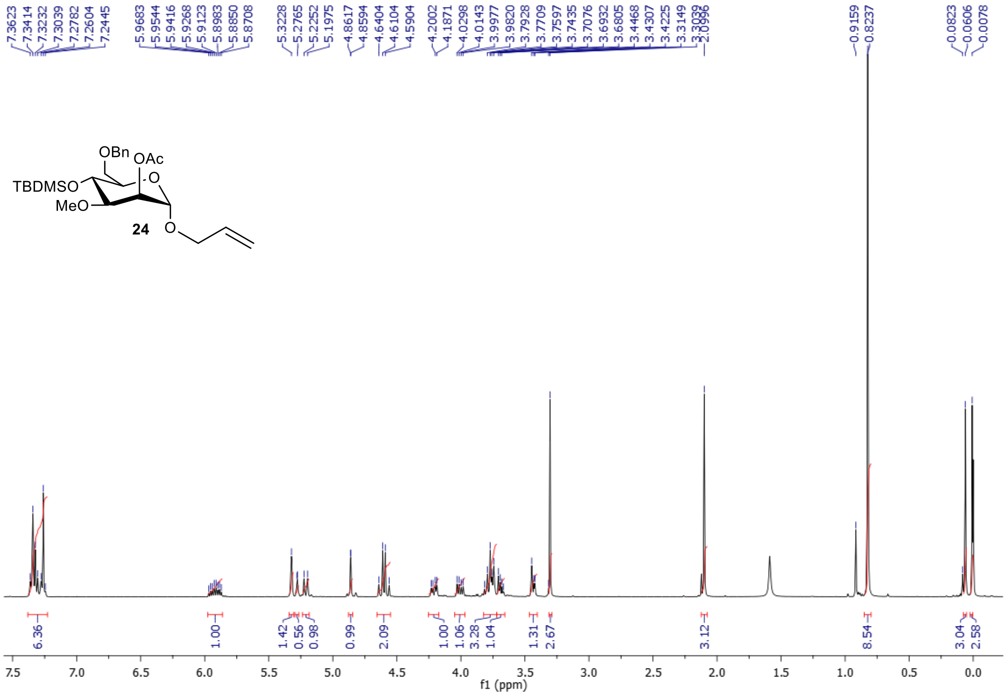

^{13}C-NMR (100.61 MHz) spectrum of allyl 2-*O*-acetyl-6-*O*-benzyl-4-*O*-*tert*-butyldimethylsilyl-3-*O*-methyl-α-D-mannopyranoside **24**.

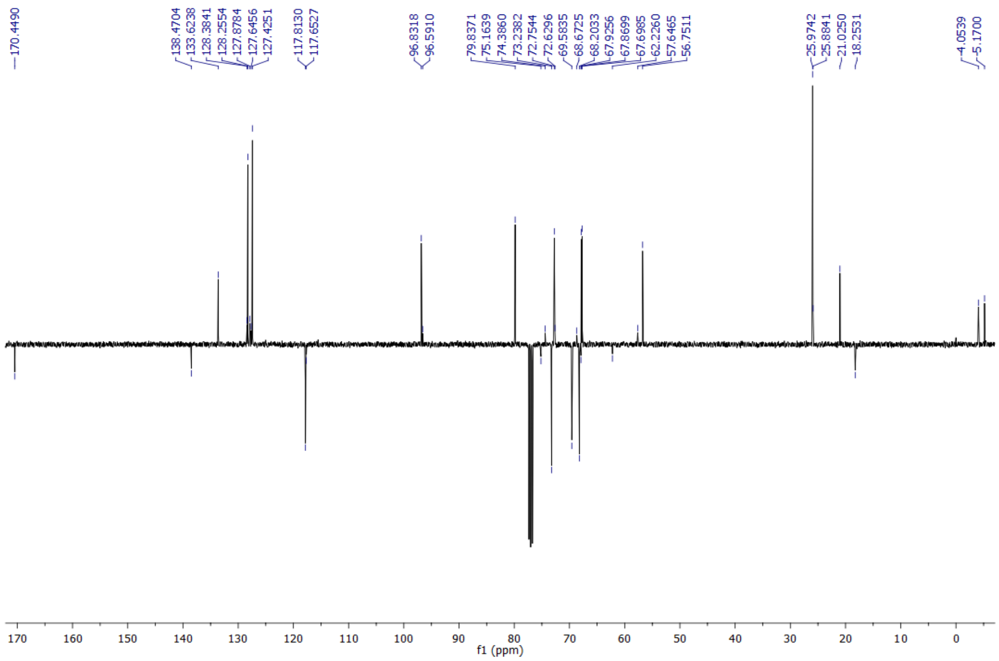

^{1}H-NMR (400 MHz) spectrum of 2-*O*-acetyl-6-*O*-benzyl-4-*O*-*tert*-butyldimethylsilyl-3-*O*-methyl-(α/β)-D-mannopyranoside **25**.

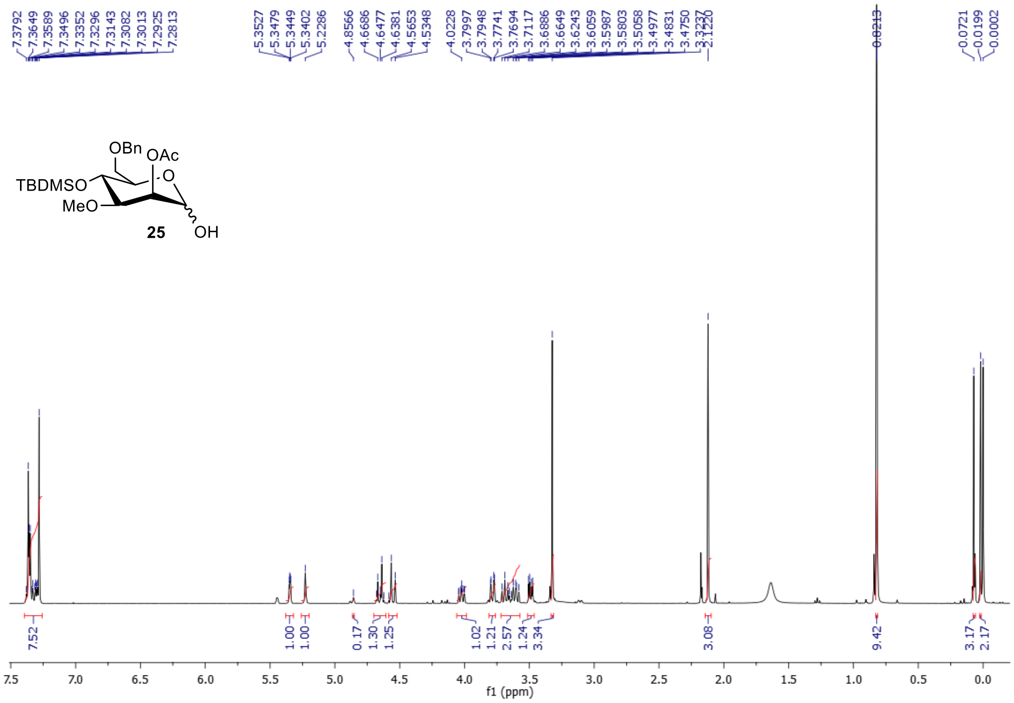

^{13}C-NMR (100.61 MHz) spectrum of 2-*O*-acetyl-6-*O*-benzyl-4-*O*-*tert*-butyldimethylsilyl-3-*O*-methyl-(α/β)-D-mannopyranoside **25**.

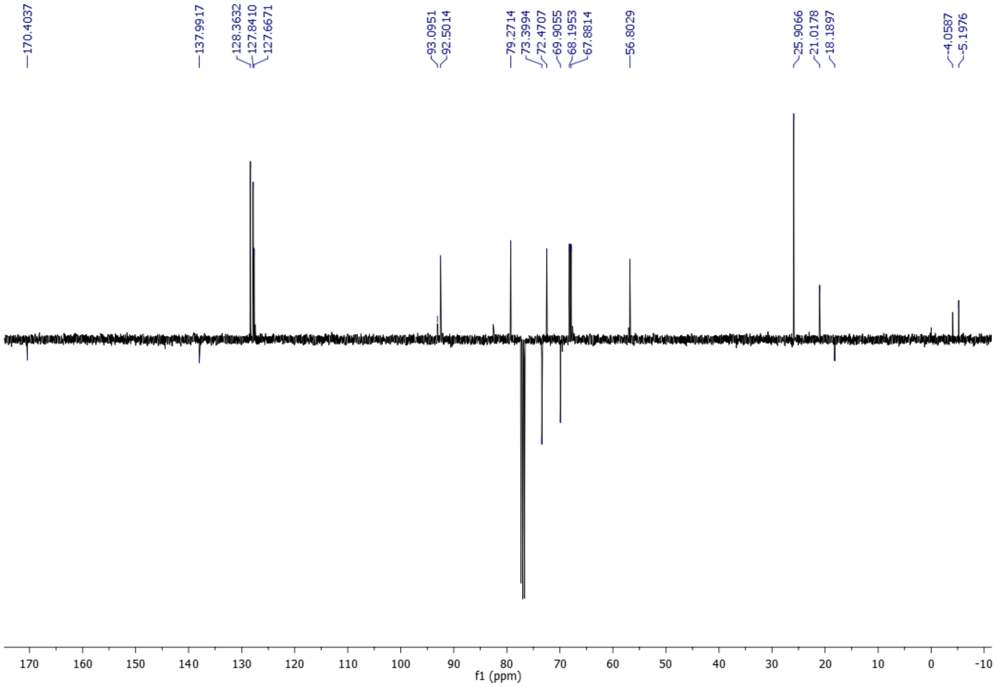

^{1}H-NMR (400 MHz) spectrum of 2-*O*-acetyl-6-*O*-benzyl-4-*O*-*tert*-butyldimethylsilyl-3-*O*-methyl-α-D-mannopyranoside trichloroacetimidate **26**.

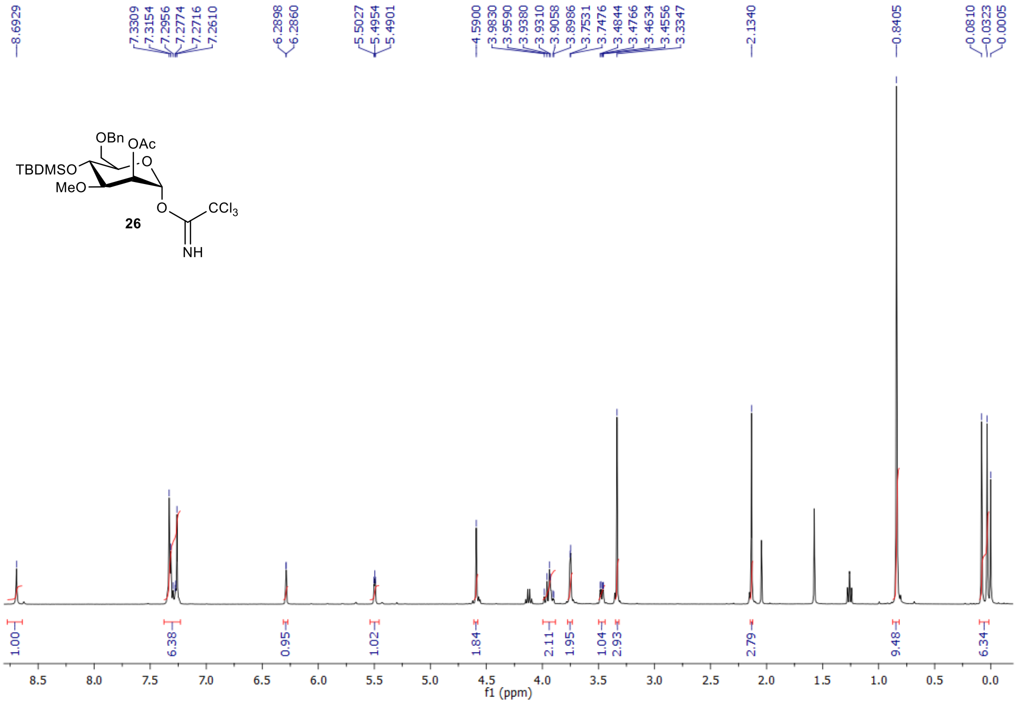

^{13}C-NMR (100.61 MHz) spectrum of 2-*O*-acetyl-6-*O*-benzyl-4-*O*-*tert*-butyldimethylsilyl-3-*O*-methyl-α-D-mannopyranoside trichloroacetamidate **26**.

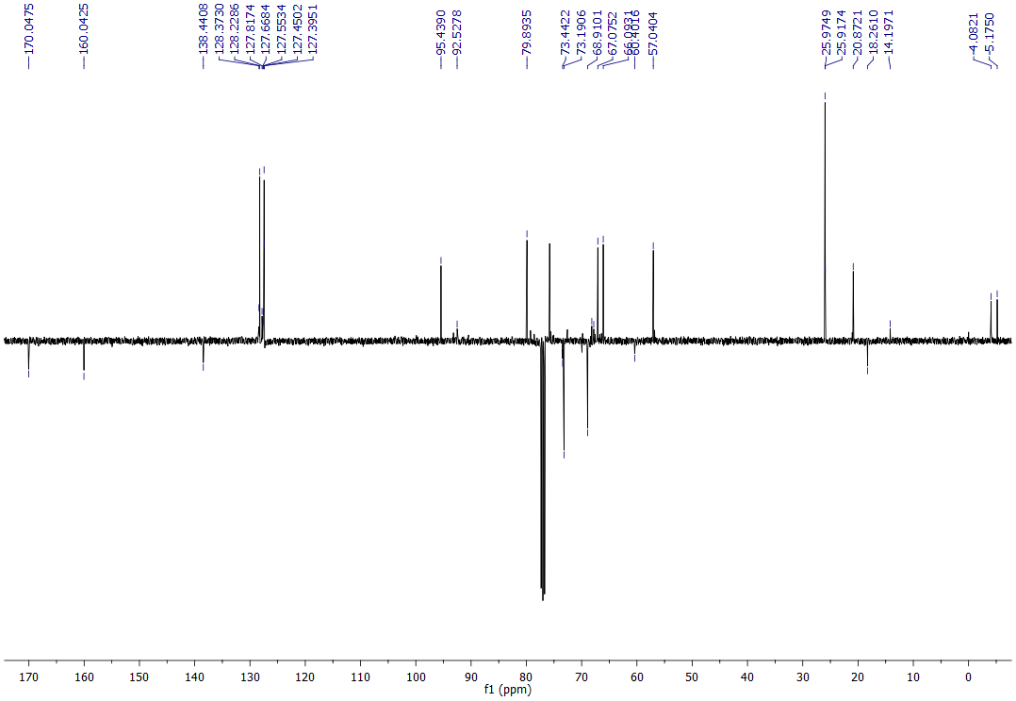

^{1}H-NMR (400 MHz) spectrum of allyl 2-*O*-acetyl-6-*O*-benzyl-3-*O*-methyl-α-D-mannopyranosyl-(1→4)-2-*O*-acetyl-6-*O*-benzyl-3-*O*-methyl-α-D-mannopyranoside **30**.

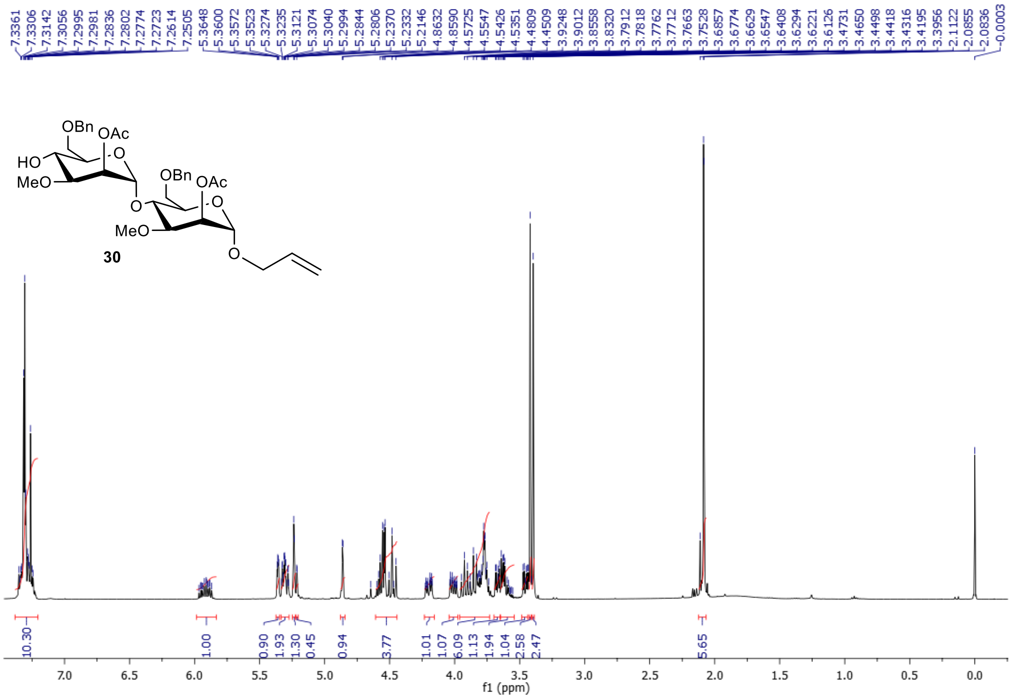

^{13}C-NMR (100.61 MHz) spectrum of allyl 2-*O*-acetyl-6-*O*-benzyl-3-*O*-methyl-α-D-mannopyranosyl-(1→4)-2-*O*-acetyl-6-*O*-benzyl-3-*O*-methyl-α-D-mannopyranoside **30**.

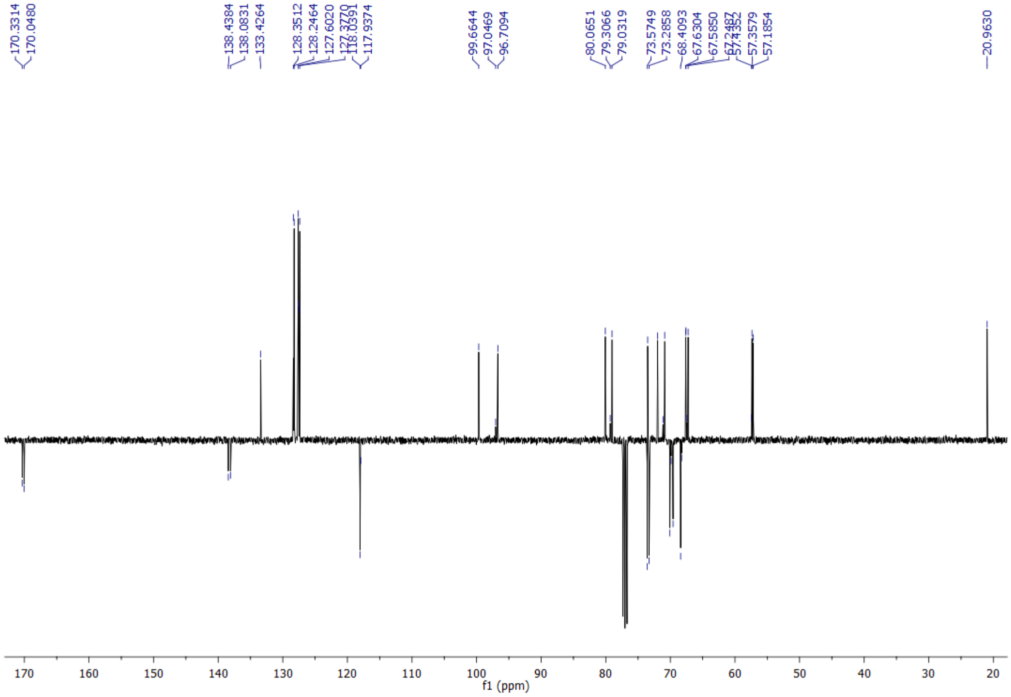

^{1}H-NMR (400 MHz) spectrum of 2,4-di-*O*-acetyl-6-*O*-benzyl-3-*O*-methyl-α-D-mannopyranosyl-(1→4)-2-*O*-acetyl-6-*O*-benzyl-3-*O*-methyl-α-D-mannopyranosyl trichloroacetimidate **33**.

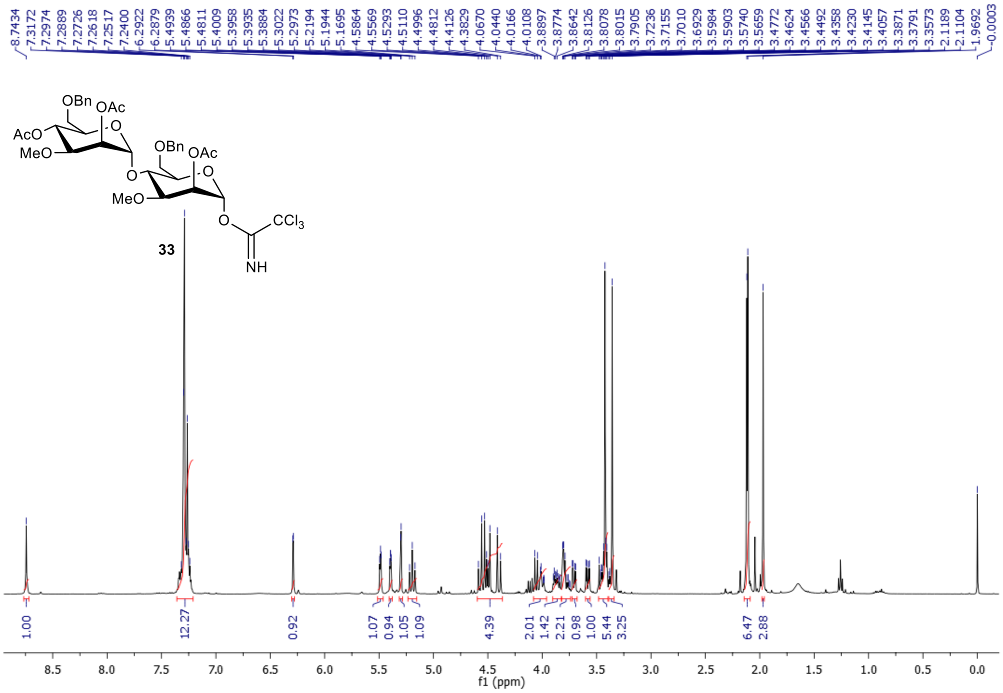

^{13}C-NMR (100.61 MHz) spectrum of 2,4-di-*O*-acetyl-6-*O*-benzyl-3-*O*-methyl-α-D-mannopyranosyl-(1→4)-2-*O*-acetyl-6-*O*-benzyl-3-*O*-methyl-α-D-mannopyranosyl trichloroacetimidate **33**.

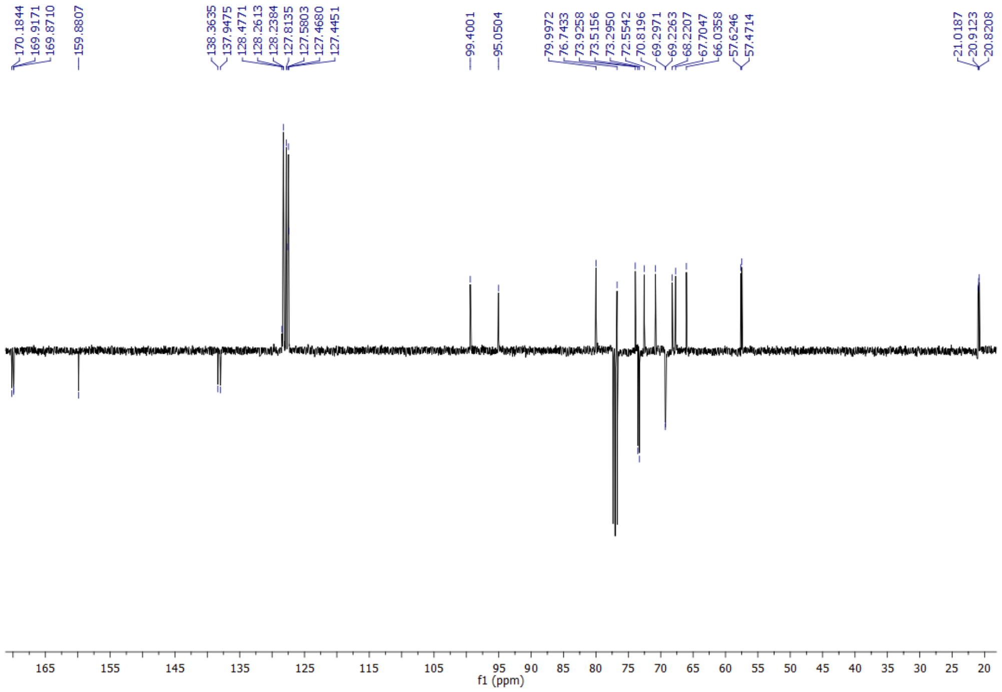

^{1}H-NMR (400 MHz) spectrum of allyl 2,4-di-*O*-acetyl-6-*O*-benzyl-3-*O*-methyl-α-D-mannopyranosyl-(1→4)-2-*O*-acetyl-6-*O*-benzyl-3-*O*-methyl-α-D-mannopyranosyl-(1→4)-2-*O*-acetyl-6-*O*-benzyl-3-*O*-methyl-α-D-mannopyranosyl-(1→4)-2-*O*-acetyl-6-*O*-benzyl-3-*O*-methyl-α-D-mannopyranoside **34**.

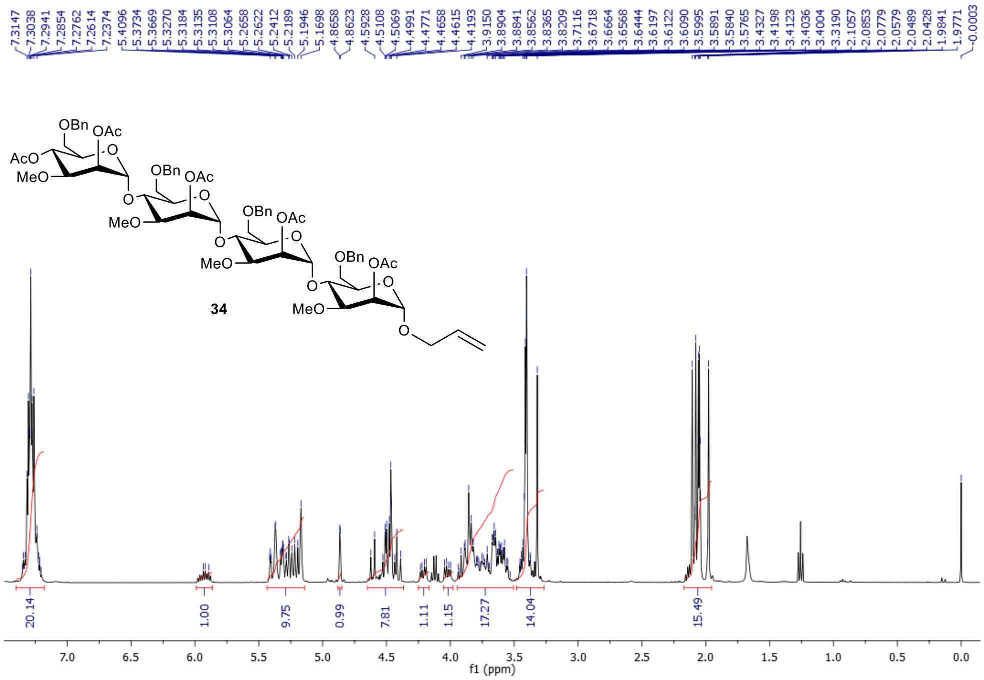

^{13}C-NMR (100.61 MHz) spectrum of allyl 2,4-di-*O*-acetyl-6-*O*-benzyl-3-*O*-methyl-α-D-mannopyranosyl-(1→4)-2-*O*-acetyl-6-*O*-benzyl-3-*O*-methyl-α-D-mannopyranosyl-(1→4)-2-*O*-acetyl-6-*O*-benzyl-3-*O*-methyl-α-D-mannopyranosyl-(1→4)-2-*O*-acetyl-6-*O*-benzyl-3-*O*-methyl-α-D-mannopyranoside **34**.

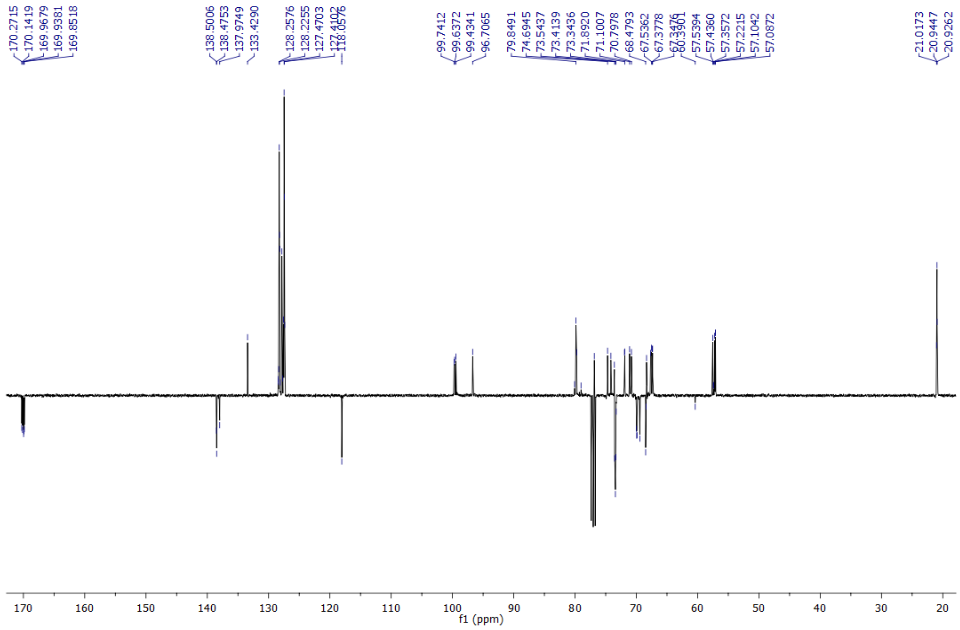

^{1}H-NMR (400 MHz) spectrum of allyl 6-*O*-benzyl-3-*O*-methyl-α-D-mannopyranosyl-(1→4)-6-*O*-benzyl-3-*O*-methyl-α-D-mannopyranosyl-(1→4)-6-*O*-benzyl-3-*O*-methyl-α-D-mannopyranosyl-(1→4)-6-*O*-benzyl-3-*O*-methyl-α-D-mannopyranoside **35**.

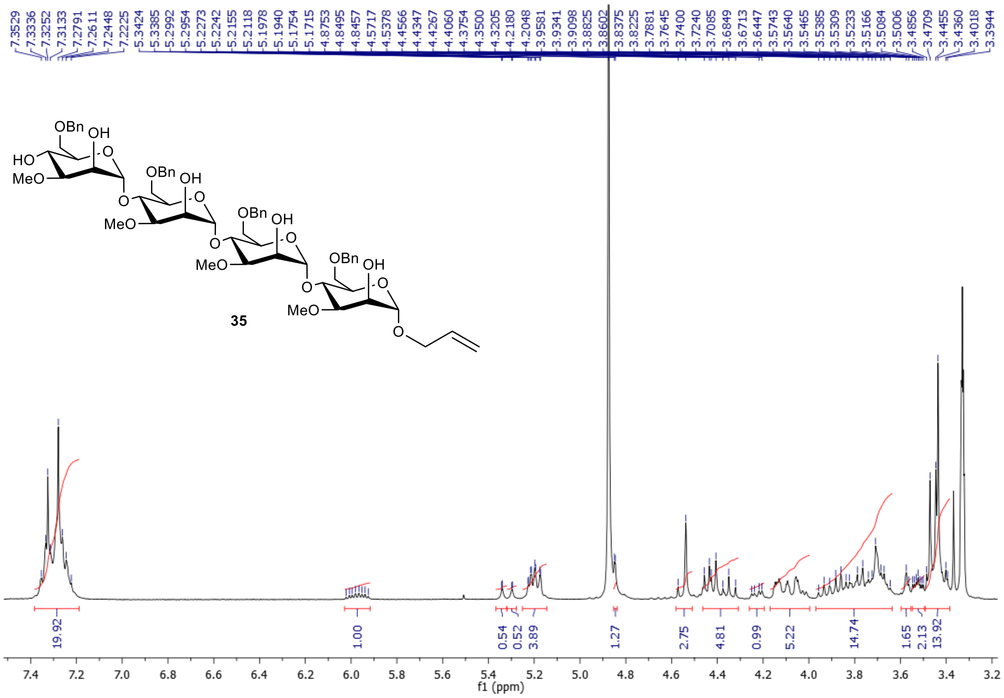

^{13}C-NMR (100.61 MHz) spectrum of allyl 6-*O*-benzyl-3-*O*-methyl-α-D-mannopyranosyl-(1→4)-6-*O*-benzyl-3-*O*-methyl-α-D-mannopyranosyl-(1→4)-6-*O*-benzyl-3-*O*-methyl-α-D-mannopyranosyl-(1→4)-6-*O*-benzyl-3-*O*-methyl-α-D-mannopyranoside **35**.

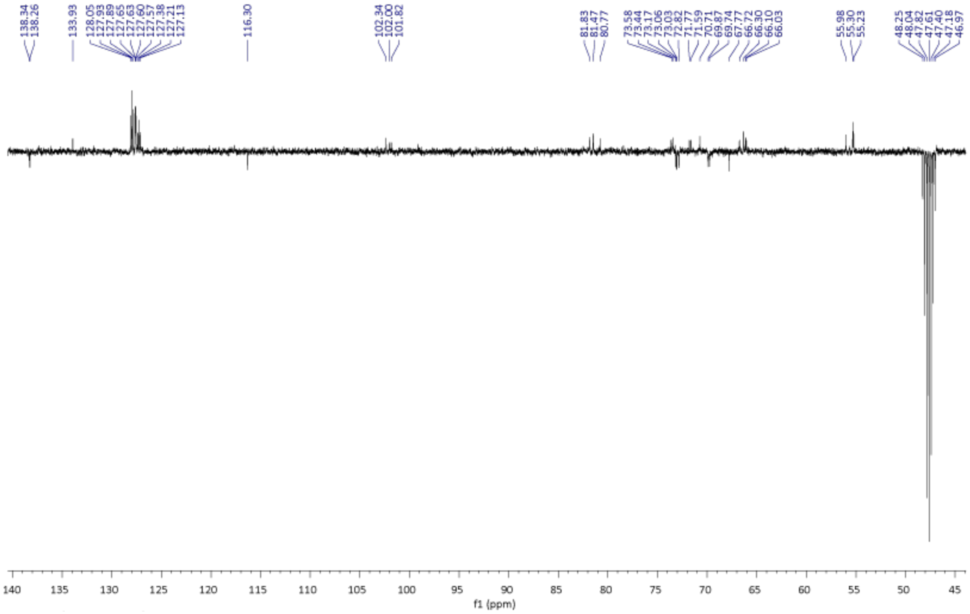

^{1}H-NMR (400 MHz) spectrum of allyl 2,4-di-*O*-acetyl-6-*O*-benzyl-3-*O*-methyl-α-D-mannopyranosyl-(1→4)-2-*O*-acetyl-6-*O*-benzyl-3-*O*-methyl-α-D-mannopyranosyl-(1→4)-2-*O*-acetyl-6-*O*-benzyl-3-*O*-methyl-α-D-mannopyranoside **36**.

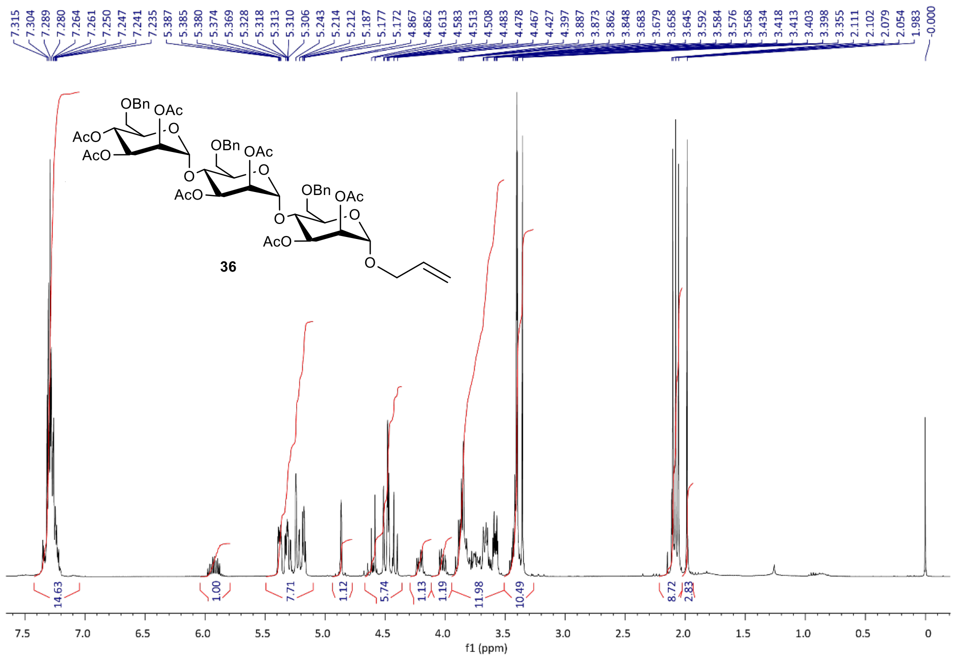

^{13}C-NMR (100.61 MHz) spectrum of allyl 2,4-di-*O*-acetyl-6-*O*-benzyl-3-*O*-methyl-α-D-mannopyranosyl-(1→4)-2-*O*-acetyl-6-*O*-benzyl-3-*O*-methyl-α-D-mannopyranosyl-(1→4)-2-*O*-acetyl-6-*O*-benzyl-3-*O*-methyl-α-D-mannopyranoside **36**.

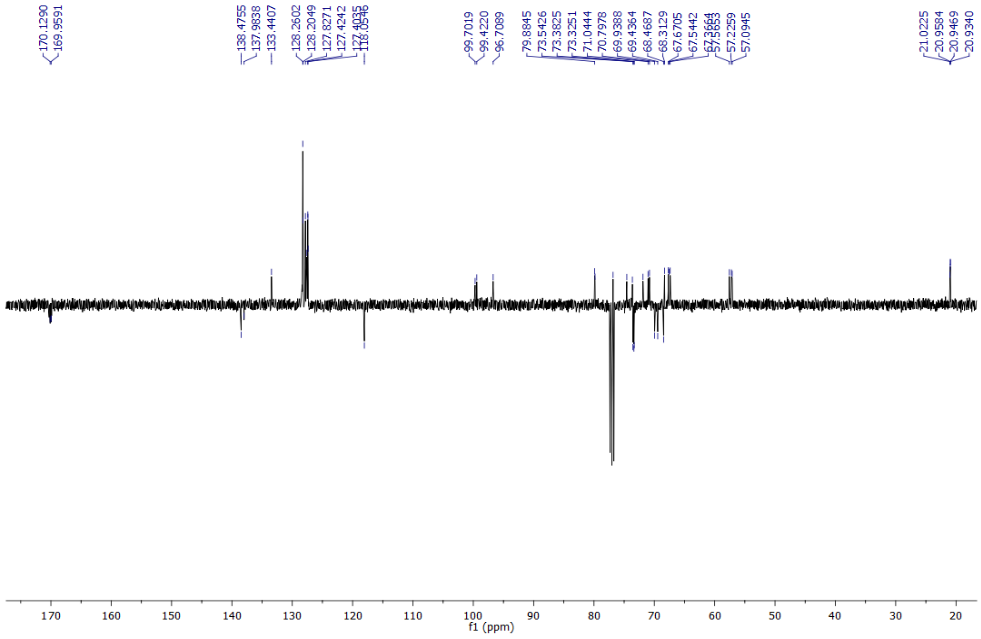

^{1}H-NMR (400 MHz) spectrum of Propyl 3-*O*-methyl-α-D-mannopyranosyl-(1→4)-3-*O*-methyl-α-D-mannopyranosyl-(1→4)-3-*O*-methyl-α-D-mannopyranosyl-(1→4)-3-*O*-methyl-α-D-mannopyranoside **3**.

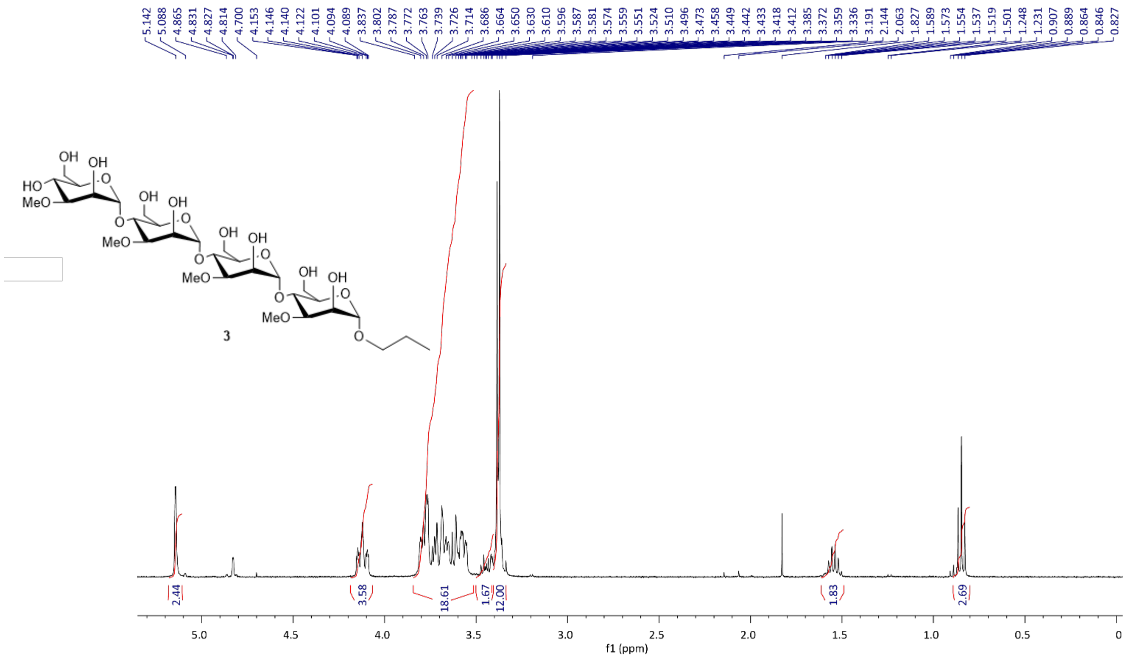

^{13}C-NMR (100.61 MHz) spectrum of Propyl 3-*O*-methyl-α-D-mannopyranosyl-(1→4)-3-*O*-methyl-α-D-mannopyranosyl-(1→4)-3-*O*-methyl-α-D-mannopyranosyl-(1→4)-3-*O*-methyl-α-D-mannopyranoside **3**.

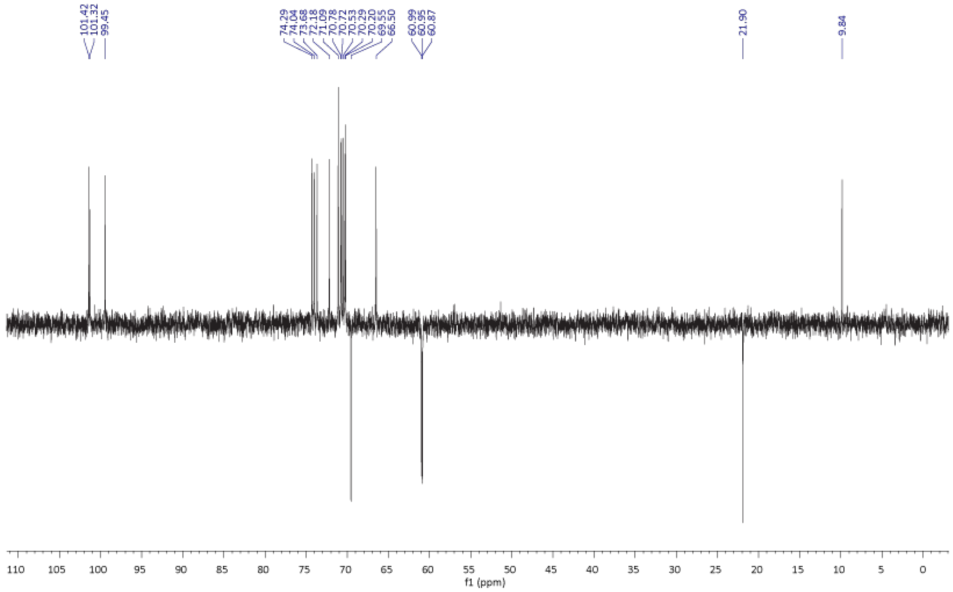

^{1}H-NMR (400 MHz) spectrum of allyl 6-*O*-benzyl-3-*O*-methyl-α-D-mannopyranosyl-(1→4)-6-*O*-benzyl-3-*O*-methyl-α-D-mannopyranosyl-(1→4)-6-*O*-benzyl-3-*O*-methyl-α-D-mannopyranoside **37**.

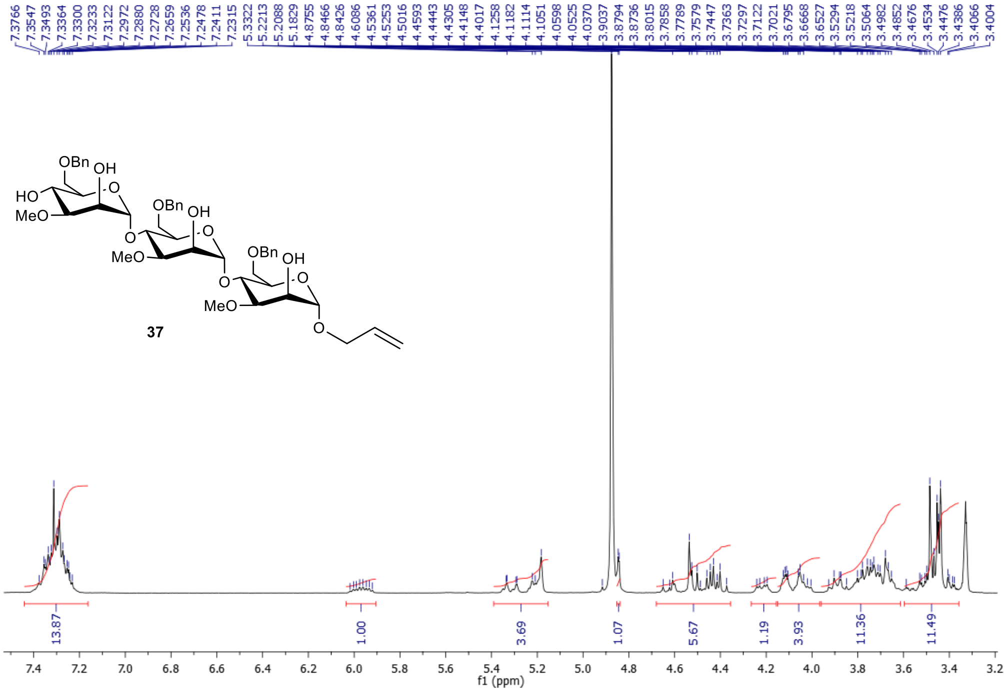

^{13}C-NMR (100.61 MHz) spectrum of allyl 6-*O*-benzyl-3-*O*-methyl-α-D-mannopyranosyl-(1→4)-6-*O*-benzyl-3-*O*-methyl-α-D-mannopyranosyl-(1→4)-6-*O*-benzyl-3-*O*-methyl-α-D-mannopyranoside **37**.

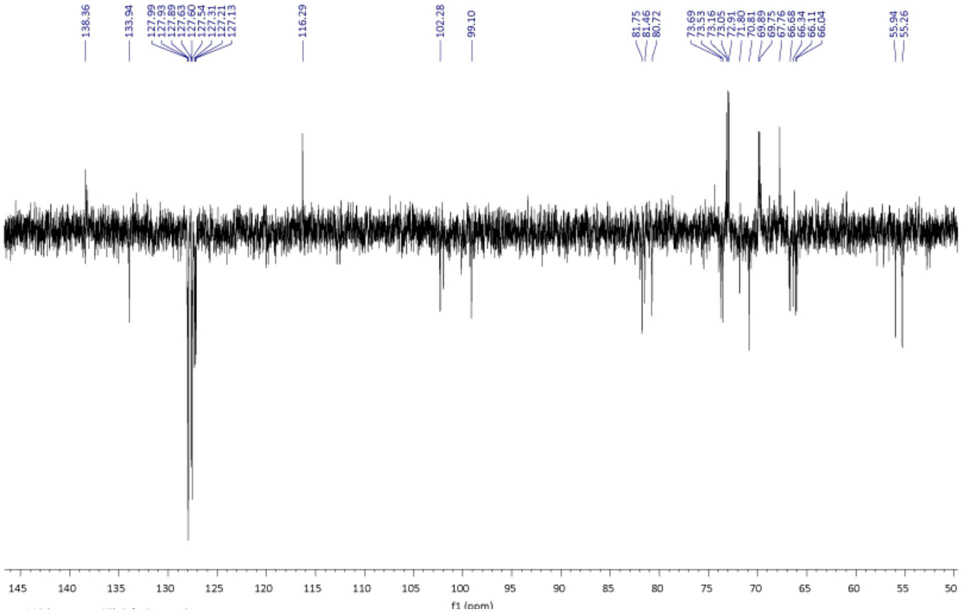

^{1}H-NMR (400 MHz) spectrum of propyl 3-*O*-methyl-α-D-mannopyranosyl-(1→4)-3-*O*-methyl-α-D-mannopyranosyl-(1→4)-3-*O*-methyl-α-D-mannopyranoside **4**.

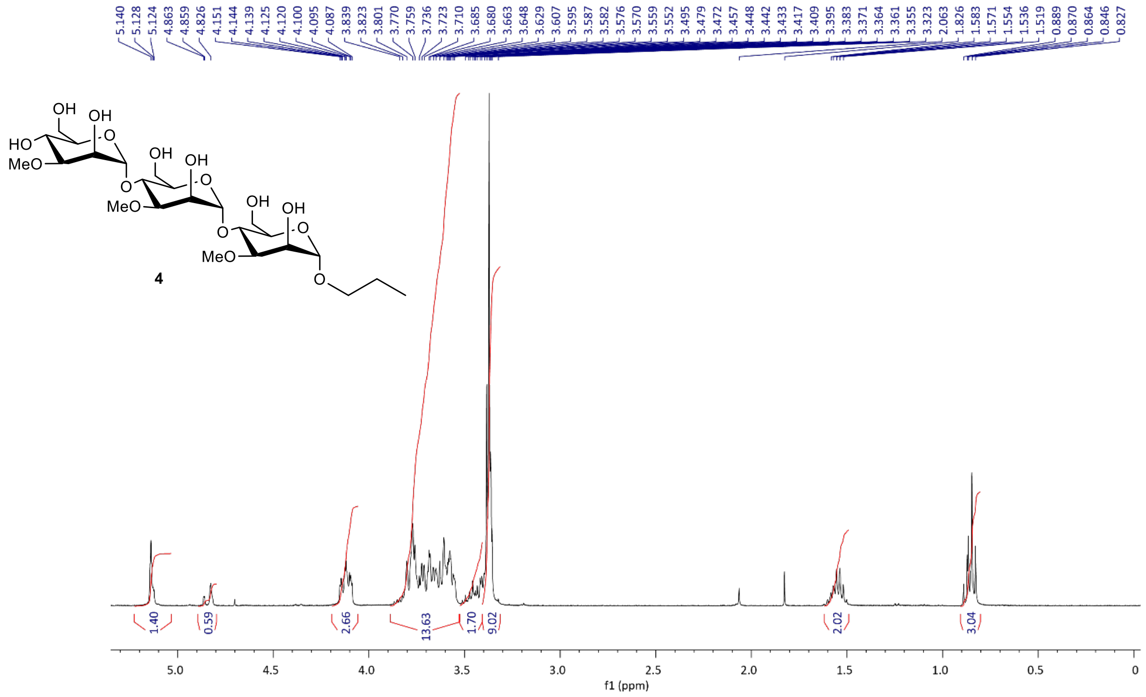

^{13}C-NMR (100.61 MHz) spectrum of propyl 3-*O*-methyl-α-D-mannopyranosyl-(1→4)-3-*O*-methyl-α-D-mannopyranosyl-(1→4)-3-*O*-methyl-α-D-mannopyranoside **4**.

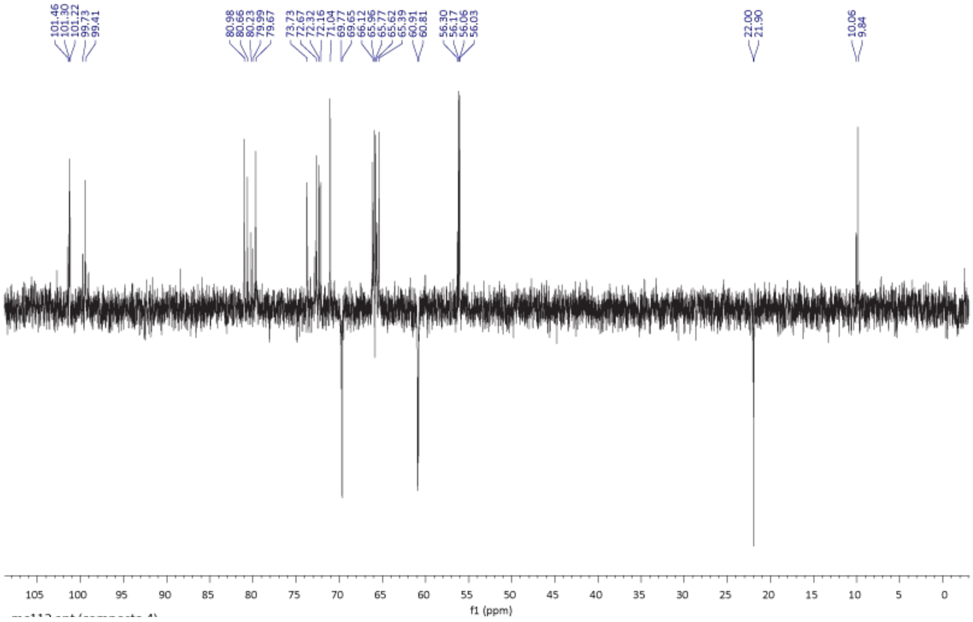

Supplementary Figures

A

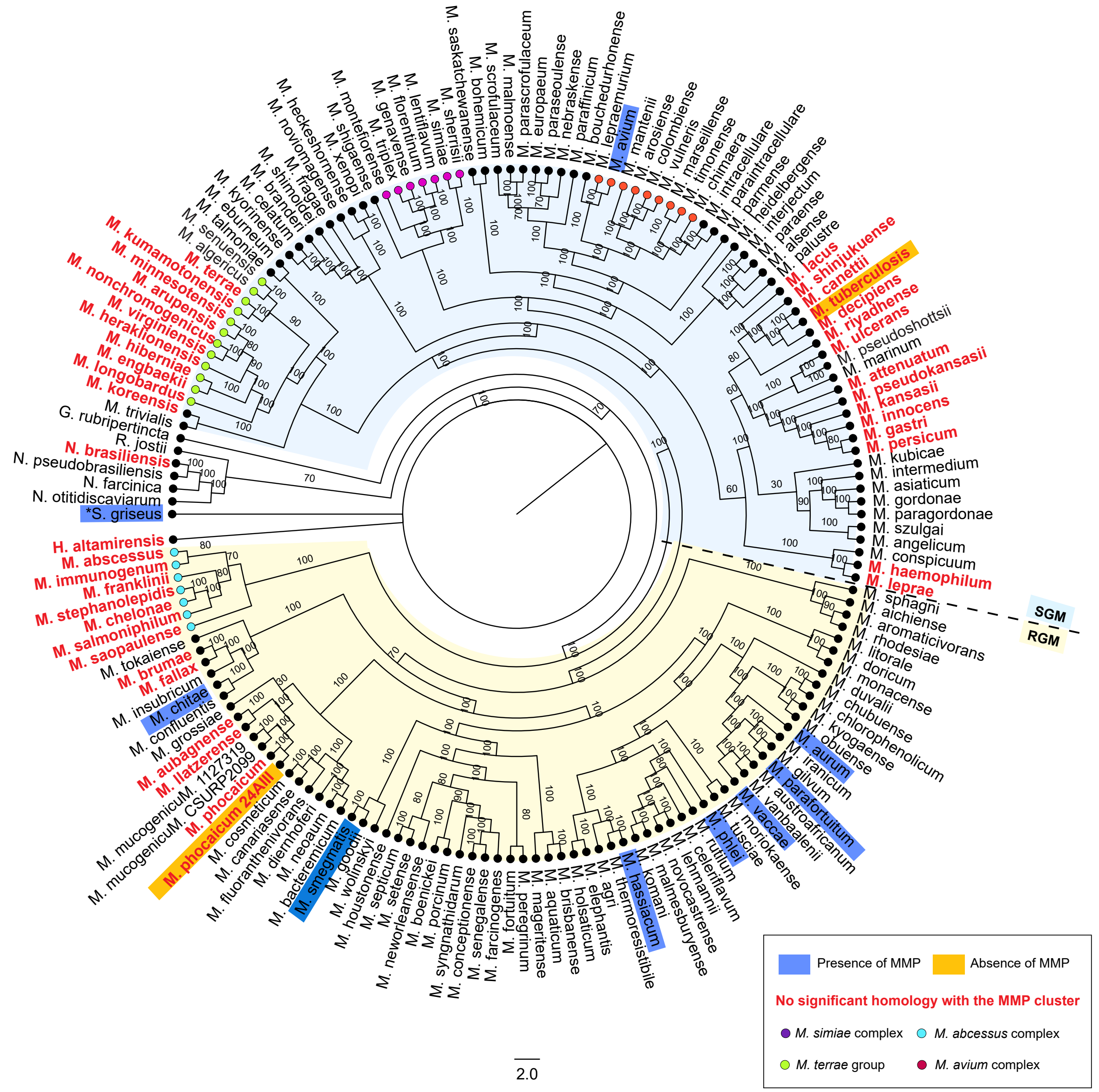
M. avium
M. hassiacum
M. smegmatis
M. chitae
M. phlei
M. vaccae
M. parafortuitum
M. aurum
*S. griseus
M. tuberculosis
M. phocaicum 24AIII
SGM
RGM
Presence of MMP
Absence of MMP
No significant homology with the MMP cluster
M. simiae complex
M. abcessus complex
M. terrae group
M. avium complex
2.0

B

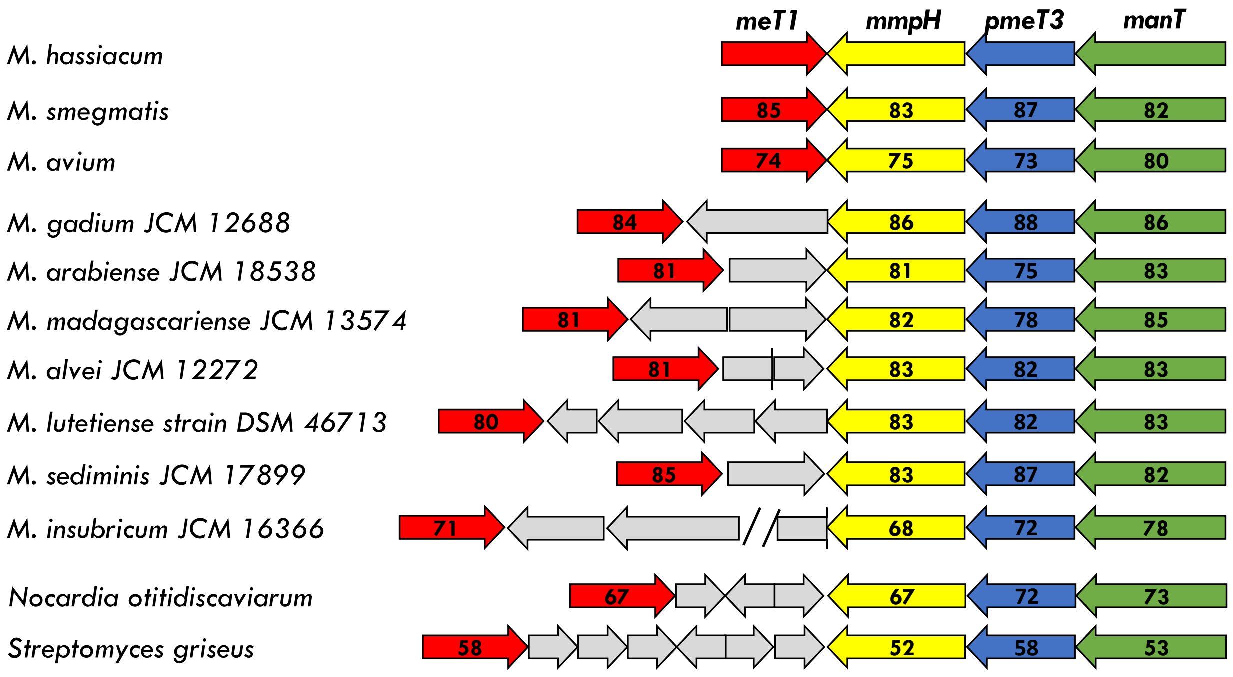
meT1
mmpH
pmeT3
manT
M. hassiacum
M. smegmatis
85
83
87
82
M. avium
74
75
73
80
M. gadium JCM 12688
84
86
88
86
M. arabiense JCM 18538
81
81
75
83
M. madagascariense JCM 13574
81
82
78
85
M. alvei JCM 12272
81
83
82
83
M. lutetiense strain DSM 46713
80
83
82
83
M. sediminis JCM 17899
85
83
87
82
M. insubricum JCM 16366
71
68
72
78
Nocardia otitidiscaviarum
67
67
72
73
Streptomyces griseus
58
52
58
53

Fig. S1. **Distribution and organization of the MMP biosynthetic gene cluster in Actinobacteria**. (**A**) Phylogenetic cladogram for the presence of the MMP gene cluster in bacterial genomes as represented in Fig. 1 indicating bootstrap branch support values. The phylogenetic relationship for the complete genomes of 162 mycobacterial species and related actinobacteria (Table S1) was inferred using the bcgTree pipeline [6]. Previous experimental evidence of the presence of MMP was taken into consideration [7, 8, 9]. If not indicated in the cladogram, the strain listed in Table S1 was considered representative of the species. **Streptomyces griseus* has an acetylated form of MMP (AMMP). (**B**) Organization of the MMP biosynthetic gene cluster (arrows) in Actinobacteria. Genome analysis of 177 species of *Mycobacterium* revealed that the arrangement of the MMP gene cluster is highly conserved in *Mycobacterium* spp. A different genomic arrangement from that of *M. hassiacum*, *M. smegmatis* and *M. avium* was only rarely detected. Red, *meT1*, 1-*O*-methyltransferase gene; yellow, *mmpH*, MMP hydrolase or MMP α–endomannosidase gene; blue, *pmeT3*, putative 3-*O*-methyltransferase gene; green, *manT*, mannosyltransferase gene. Percentage of amino acid sequence identity to *M. hassiacum* proteins is indicated inside arrows. Grey arrows indicate genes absent from the typical MMP gene cluster.

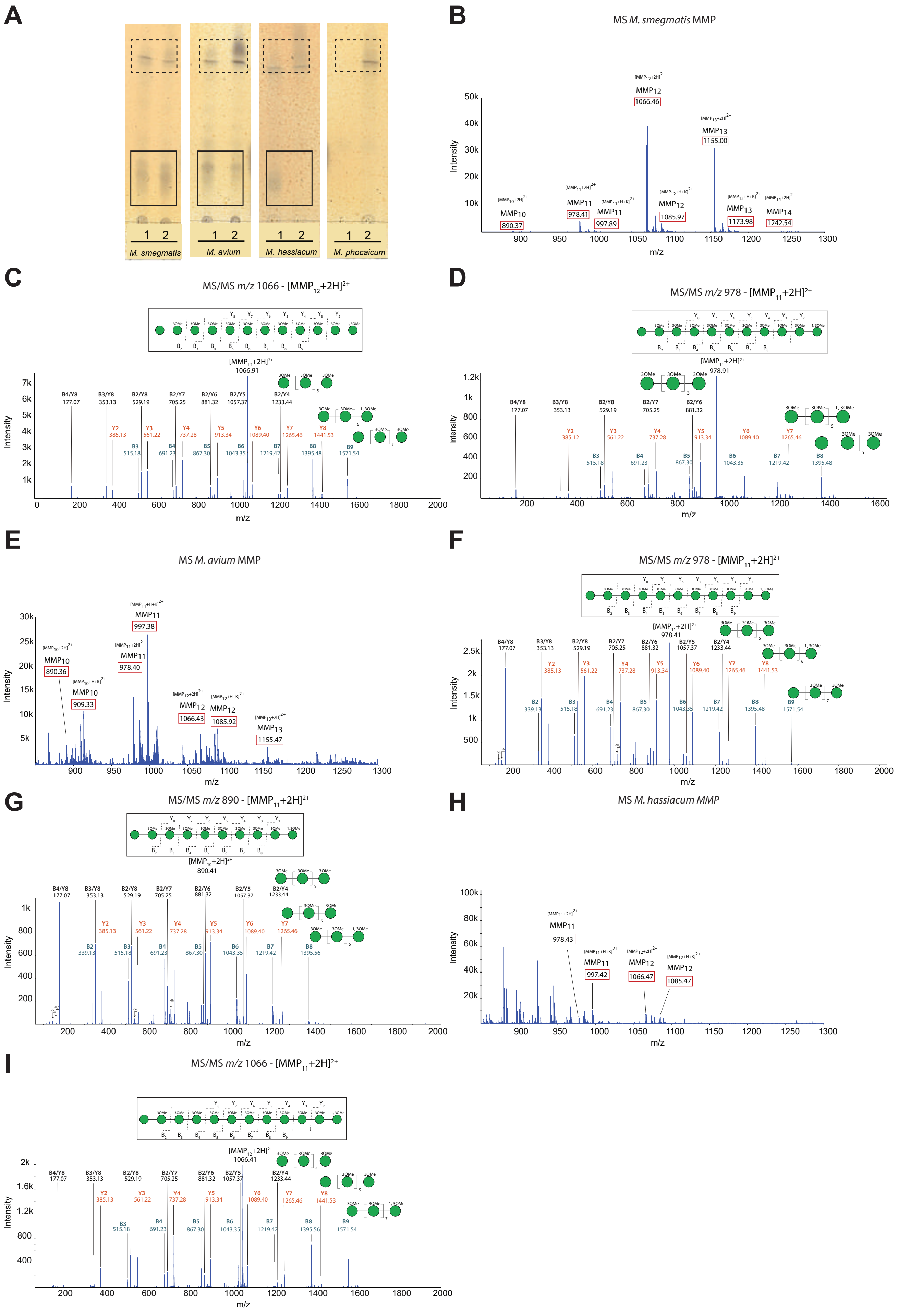
A
1 2
M. smegmatis
M. avium
M. hassiacum
M. phocaicum
B
MS M. smegmatis MMP
MMP10 890.37
MMP11 978.41
MMP11 997.89
MMP12 1066.46
MMP12 1085.97
MMP13 1155.00
MMP13 1173.98
MMP14 1242.54
Intensity
m/z
C
MS/MS m/z 1066 - [MMP12+2H]2+
[MMP12+2H]2+ 1066.91
D
MS/MS m/z 978 - [MMP11+2H]2+
[MMP11+2H]2+ 978.91
E
MS M. avium MMP
MMP10 890.36
MMP10 909.33
MMP11 978.40
MMP11 997.38
MMP12 1066.43
MMP12 1085.92
MMP13 1155.47
F
MS/MS m/z 978 - [MMP11+2H]2+
[MMP11+2H]2+ 978.41
G
MS/MS m/z 890 - [MMP11+2H]2+
[MMP10+2H]2+ 890.41
H
MS M. hassiacum MMP
MMP11 978.43
MMP11 997.42
MMP12 1066.47
MMP12 1085.47
I
MS/MS m/z 1066 - [MMP11+2H]2+
[MMP12+2H]2+ 1066.41

Fig. S2. Analysis of the content of polymethylated polysaccharides in different mycobacteria. (**A**) Extraction of polymethylated polysaccharides from *M. smegmatis, M. avium, M. hassiacum, M. phocaicum* (see Table S2) and TLC analysis were performed as described in the Methods section. Fractions 1 and 2 were eluted with 60 and 80% (v/v) methanol, respectively. Rectangles delimit the spots corresponding to MGLP (dashed line) and MMP (solid line). (**B**-**I**) Positive ion mode ESI-TOF spectra of MMP samples. The proposed assignment of the glycan moieties was based on the ESI-MS/MS analyses and on published structures [10, 11]. (**B**) MS spectrum of *M. smegmatis* MMP; (**C**) MS/MS spectrum of the *M. smegmatis* MMP ion at m/z 1066 assigned to $[MMP_{12}+2H]^{2+}$; (**D**) MS/MS spectrum of the *M. smegmatis* MMP ion at m/z 978 assigned to $[MMP_{11}+2H]^{2+}$ (**E**) MS spectrum of *M. avium* MMP; (**F**) MS/MS spectrum of the *M. avium* MMP ion at m/z 978 assigned to $[MMP_{11}+2H]^{2+}$; (**G**) MS/MS spectrum of the *M. avium* MMP ion at m/z 890 assigned to $[MMP_{10}+2H]^{2+}$; (**H**) MS spectrum of *M. hassiacum* MMP; (**I**) MS/MS spectrum of the *M. hassiacum* MMP ion at m/z 1066 assigned to $[MMP_{12}+2H]^{2+}$. Product ion nomenclature follows that proposed by Domon and Costello [12]. Fragmentation pathways of the Y- and B-type glycosidic cleavages are shown. The representation of glycans follows the guidelines of Symbol Nomenclature for Glycans [13].

A

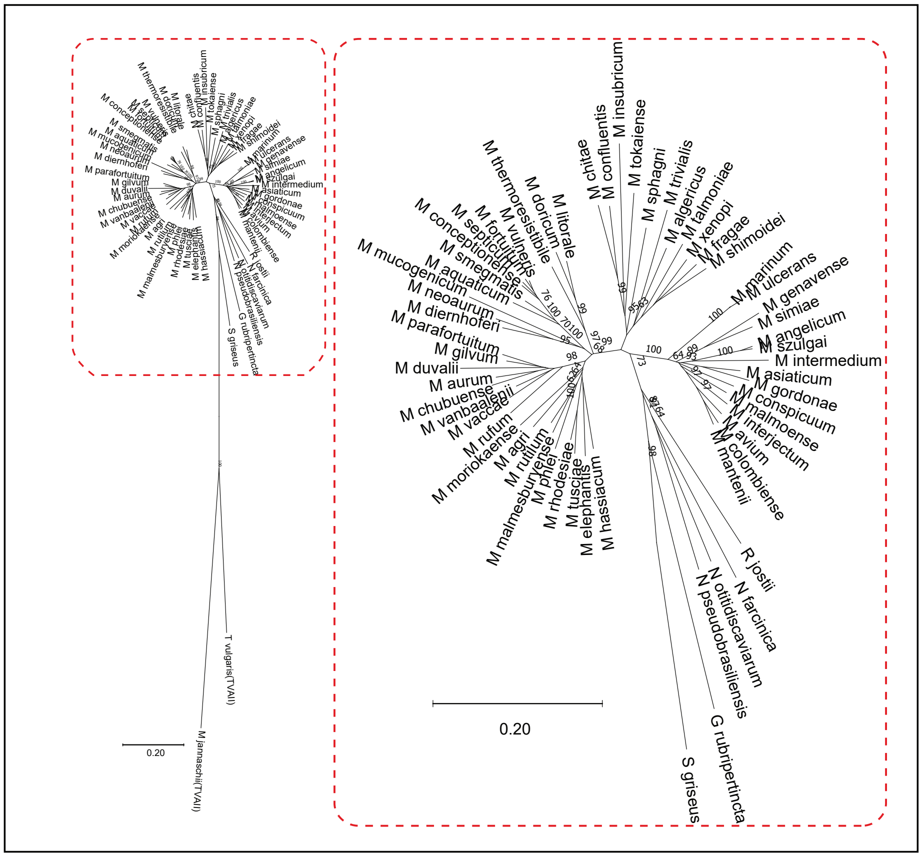

M insubricum
M confluentis
M chitae
M tokaiense
M sphagni
M trivialis
M algericus
M talmoniae
M xenopi
M fragae
M shimoidei
M marinum
M ulcerans
M genavense
M simiae
M angelicum
M szulgai
M intermedium
M asiaticum
M gordonae
M conspicuum
M malmoense
M interjectum
M avium
M colombiense
M mantenii
R jostii
N farcinica
N otitidiscaviarum
N pseudobrasiliensis
G rubripertincta
S griseus
M hassiacum
M elephantis
M tusciae
M rhodesiae
M phlei
M rutilum
M malmesburyense
M moriokaense
M agri
M rufum
M vaccae
M vanbaalenii
M chubuense
M aurum
M duvalii
M gilvum
M parafortuitum
M diernhoferi
M neoaurum
M mucogenicum
M aquaticum
M smegmatis
M conceptionense
M septicum
M fortuitum
M vulneris
M thermoresistibile
M doricum
M litorale
T vulgaris(TVAII)
M jannaschii(TVAII)
0.20

B

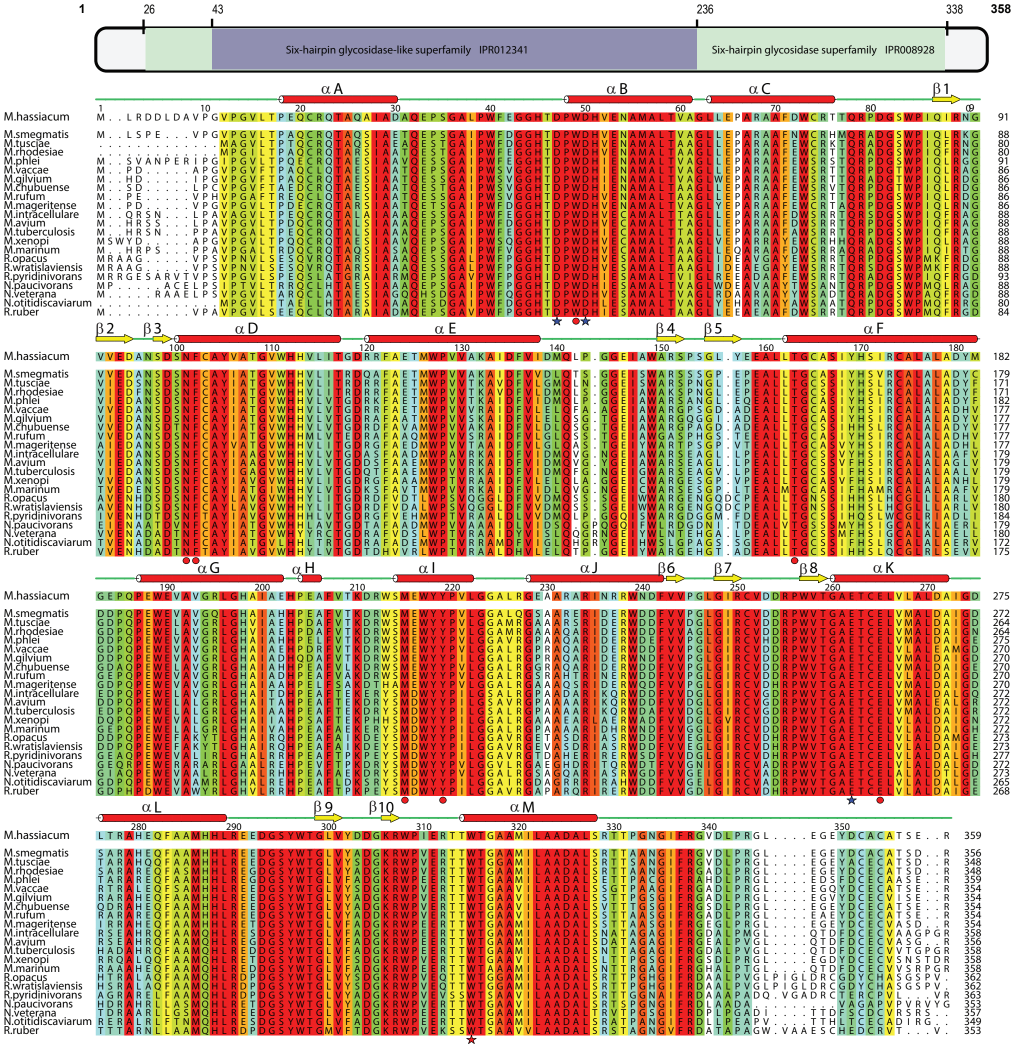

Six-hairpin glycosidase-like superfamily IPR012341
Six-hairpin glycosidase superfamily IPR008928
M.hassiacum
M.smegmatis
M.tusciae
M.rhodesiae
M.phlei
M.vaccae
M.gilvum
M.chubuense
M.rufum
M.mageritense
M.intracellulare
M.avium
M.tuberculosis
M.xenopi
M.marinum
R.opacus
R.wratislaviensis
R.pyridinivorans
N.paucivorans
N.veterana
N.otitidiscaviarum
R.ruber

Fig. S3. Analysis of the amino acid sequences of MmpH and relevant homologues. (**A**) Phylogenetic reconstruction of 61 MmpH amino acid sequences from actinobacteria and relevant distant homologues (Table S5) using the Neighbor-Joining method [14]. The optimal tree is drawn to scale and evolutionary distances were computed using the Poisson correction method [15]. The scale bar indicates the number of amino acid substitutions per site. Analyses were performed with MEGA X [16]. The only characterized sugar hydrolases with minimal detectable sequence homology with MmpH were the glucoamylase-type enzyme (TGA) from *Thermoactinomyces vulgaris* and from *Methanococcus jannaschii* [17]. These enzymes most efficiently hydrolyze small maltooligosaccharides such as maltotetraose and maltose. (**B**) Representation of protein families and functionally important domains predicted using InterProScan [18] and amino acid sequence alignment of representative MmpH orthologues from Actinobacteria. Background colors represent residue conservation (red: identical residues, orange to blue: decreasing conservation of amino acid properties; white: dissimilar residues). The amino acid numbering and the secondary structure elements depicted above the alignment are based on the MmpH three-dimensional structure (same color code as in Fig. 3A). Predicted residues responsible for substrate recognition at the (-1) subsite (red stars; except for the catalytic base (Glu262) and acid (Asp47 or Asp50) presumably responsible for the inverting mechanism (blue)) and the adjacent subsite for MmpH endo-mannosidase activity (red circles) are marked below the alignment. Figure prepared with Aline [19].

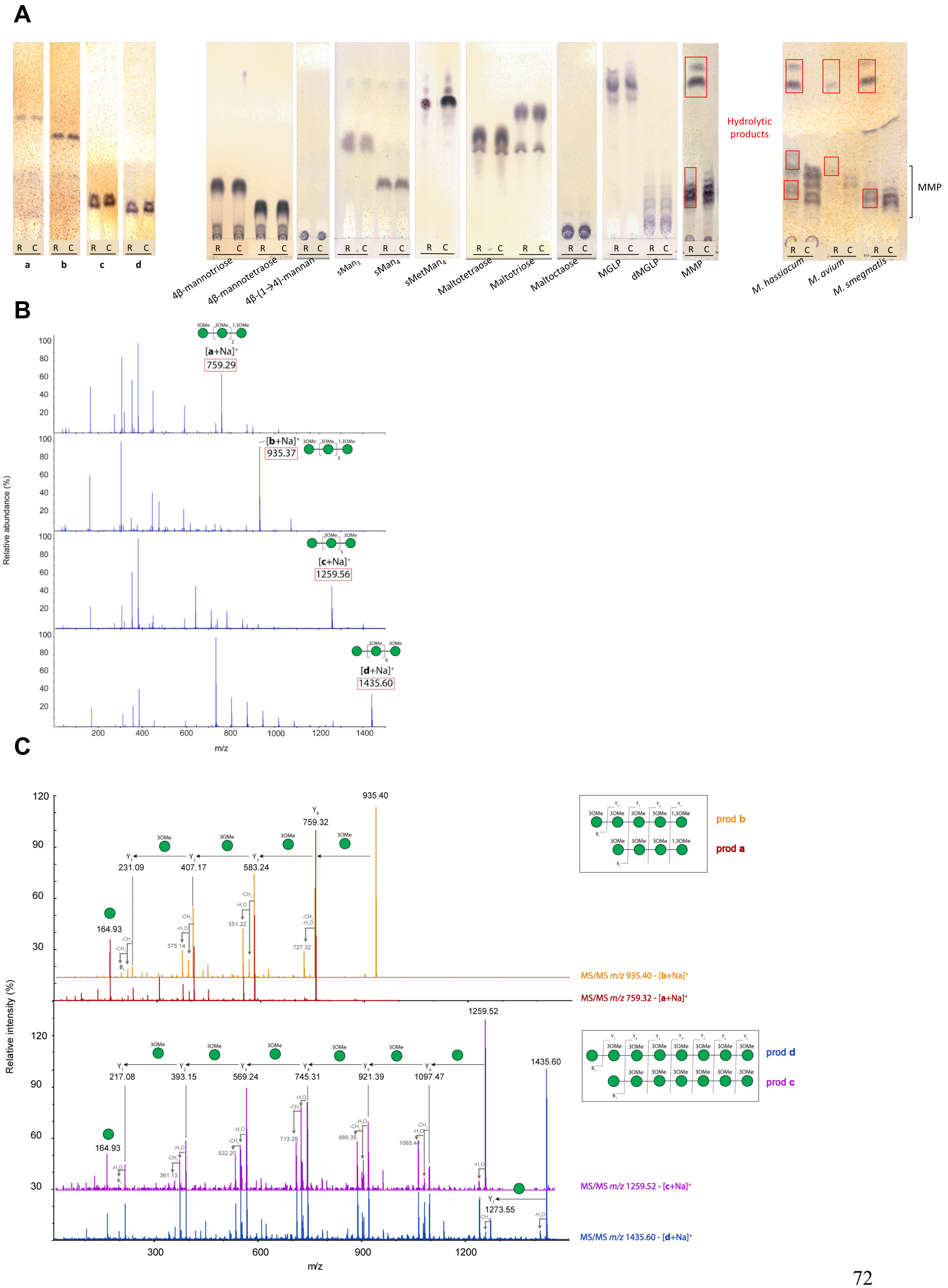
A
R C
a
b
c
d
4β-mannotriose
4β-mannotetraose
4β-(1→4)-mannan
sMan3
sMan4
sMetMan4
Maltotetraose
Maltotriose
Maltoctaose
MGLP
dMGLP
MMP
Hydrolytic products
M. hassiacum
M. avium
M. smegmatis
MMP
B
[a+Na]+
759.29
[b+Na]+
935.37
[c+Na]+
1259.56
[d+Na]+
1435.60
Relative abundance (%)
m/z
C
935.40
759.32
231.09
407.17
583.24
164.93
prod b
prod a
MS/MS m/z 935.40 - [b+Na]+
MS/MS m/z 759.32 - [a+Na]+
1259.52
1435.60
217.08
393.15
569.24
745.31
921.39
1097.47
1273.55
prod d
prod c
MS/MS m/z 1259.52 - [c+Na]+
MS/MS m/z 1435.60 - [d+Na]+
Relative intensity (%)
m/z

Fig. S4. Analysis of MmpH activity. (**A**) TLC analysis of MmpH activity: left panel - using its own reaction products as substrate; middle panel - toward 4β-mannotriose, 4β-mannotetraose, β-(1→4)-mannan, synthetic 4α-oligomannosides (sMan$_3$, sMan$_4$, sMetMan$_4$), maltotetraose, maltopentaose, maltooctaose, MGLP, deacylated MGLP (dMGLP) and MMP; right panel - toward MMP extracted from *M. hassiacum*, *M. avium* and *M. smegmatis*. R, reaction in the presence of MmpH; C, control reaction in the absence of MmpH. Purified products: **a**, tetramannoside; **b**, pentamannoside; **c**, heptamannoside; **d**, octamannoside. Synthetic 4α-oligomannosides: propylated 4α-mannotriose (sMan$_3$), 4α-mannotetraose (sMan$_4$) and 3,3',3'',3'''-tetra-*O*-methyl-4α-mannotetraose (sMetMan$_4$). MmpH hydrolytic products are indicated by red boxes. (**B**) MS and (**C**) MS/MS analysis of purified mannosyl oligosaccharides after hydrolysis of MMP with MmpH. ESI-TOF spectra acquired in positive ion mode of samples **a** to **d.** The $[M+Na]^+$ ions for each sample are boxed in red. The structures overlaid on the spectra correspond to the molecules identified in each sample. MS/MS analysis of samples a to d showing typical fragments formed under ESI-MS/MS conditions of $[M+Na]^+$ ions of mannosyl oligosaccharides. Loss of a mannose (-162 Da) or methyl mannose (-176 Da) residue due to glycosidic bond cleavage are the predominant events, as well as loss of water (-18 Da) and methylation (-14 Da)[20]. The representation of glycans follows the guidelines of Symbol Nomenclature for Glycans [13]. A filled green circle represents a mannose residue.

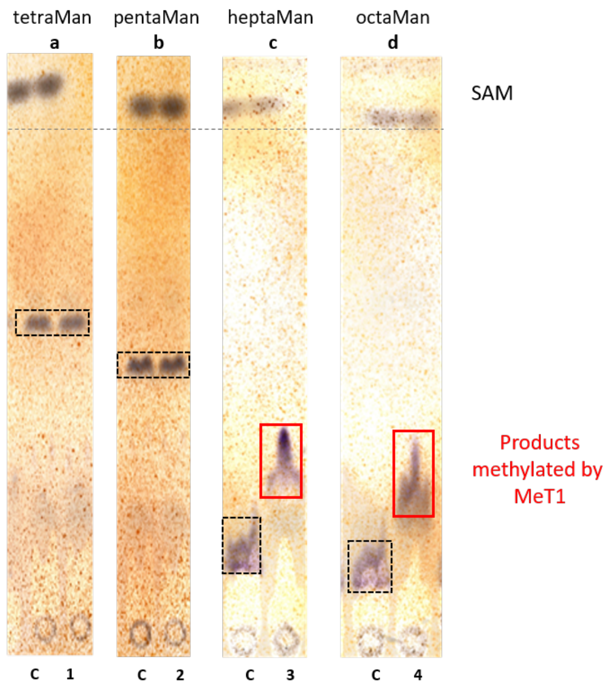

Fig. S5. TLC analysis of MeT1 activity using the natural MmpH products as substrates. Lane 1, reaction with tetramannoside **a**; lane 2, reaction with pentamannoside **b**; lane 3, reaction with heptamannoside **c**; lane 4, reaction with octamannoside **d**; C, control reactions without MeT1. MeT1 products are indicated in red boxes. MmpH products **a** to **d** are indicated in dashed black boxes. SAM, S-adenosyl-methionine.

A

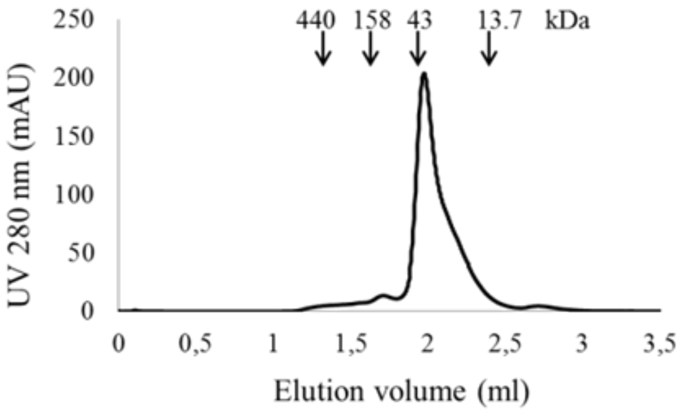

B

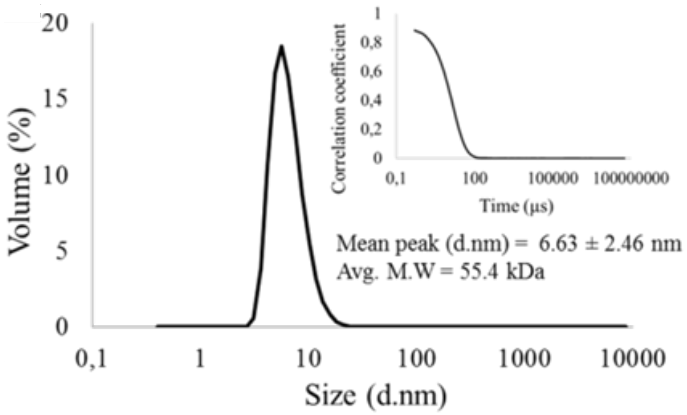

Fig. S6. Analysis of the oligomeric state of MmpH. Analysis of the oligomeric state of MmpH by SEC and DLS. (**A**) Analytical size exclusion chromatography profile. Purified MmpH (60 µg) was separated on a Superdex 200 5/150 column (GE Healthcare) with 20 mM BTP pH 7.5, 100 mM NaCl as mobile phase. Elution volumes of protein standards are indicated by arrows. (**B**) Size distribution by volume of MmpH (at 1 mg/mL), determined by DLS in the same buffer as in (**A**). The hydrodynamic radius of the mean peak, its corresponding average molecular weight and the correlation coefficient plot are also represented.

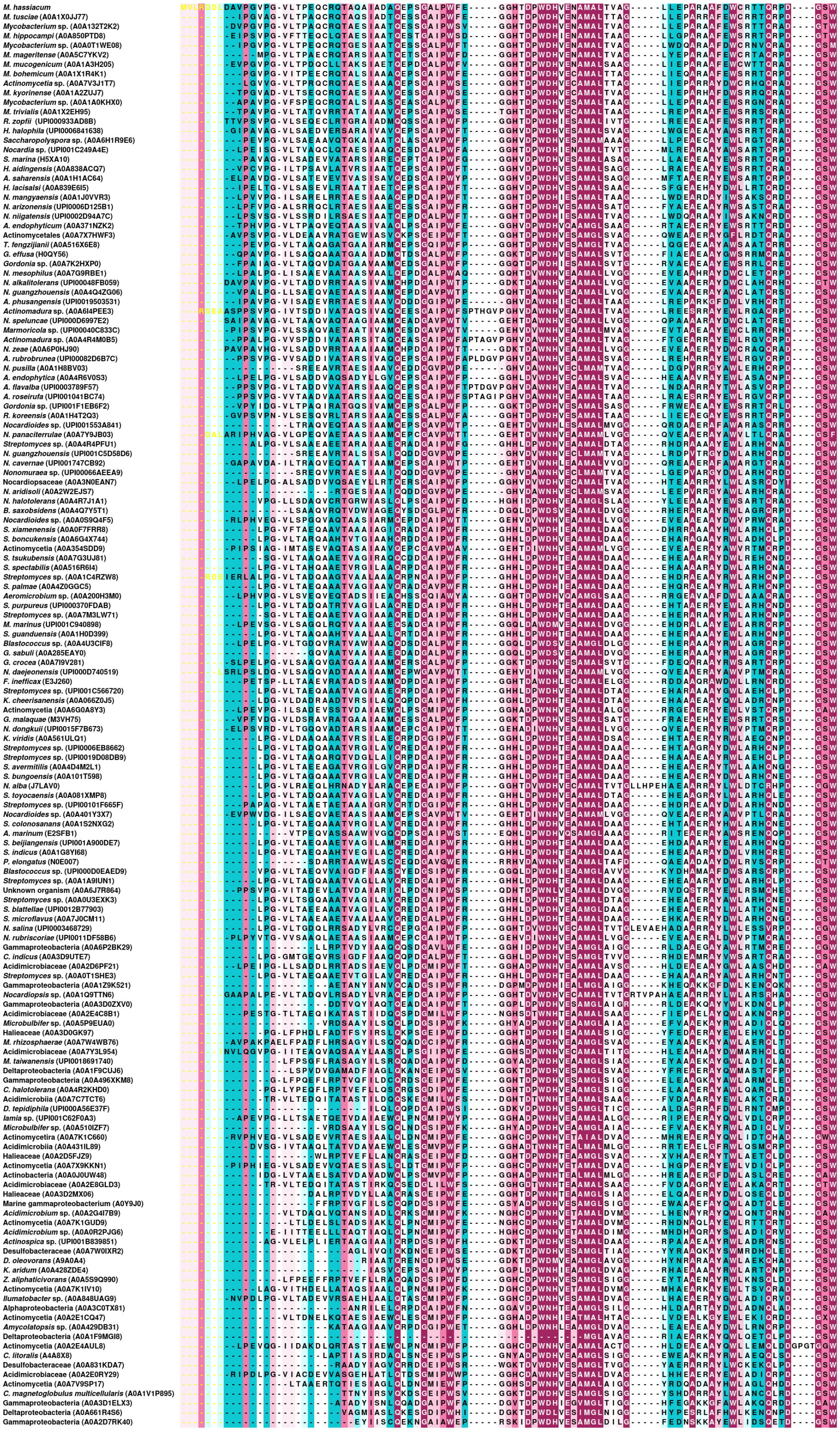

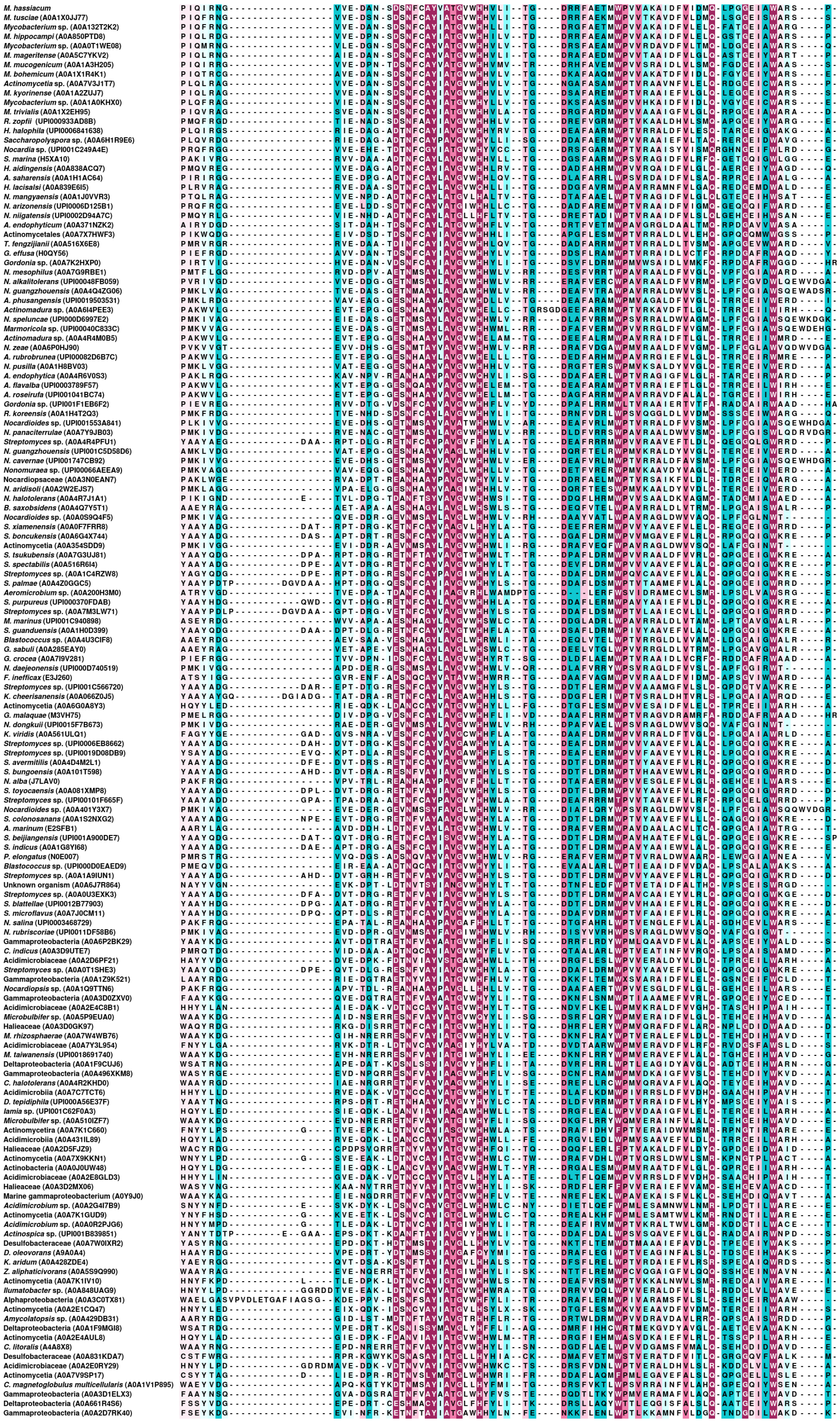

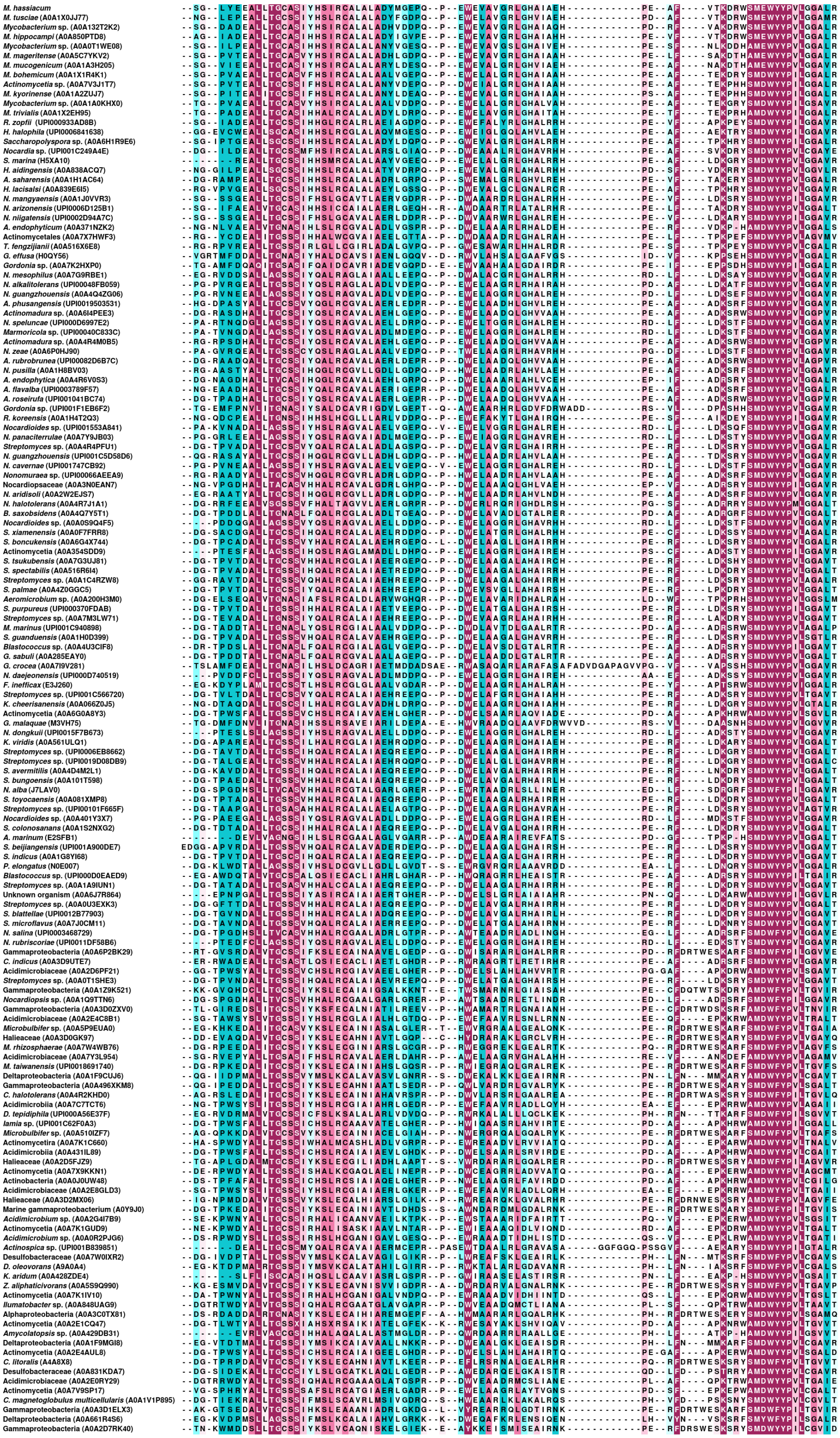

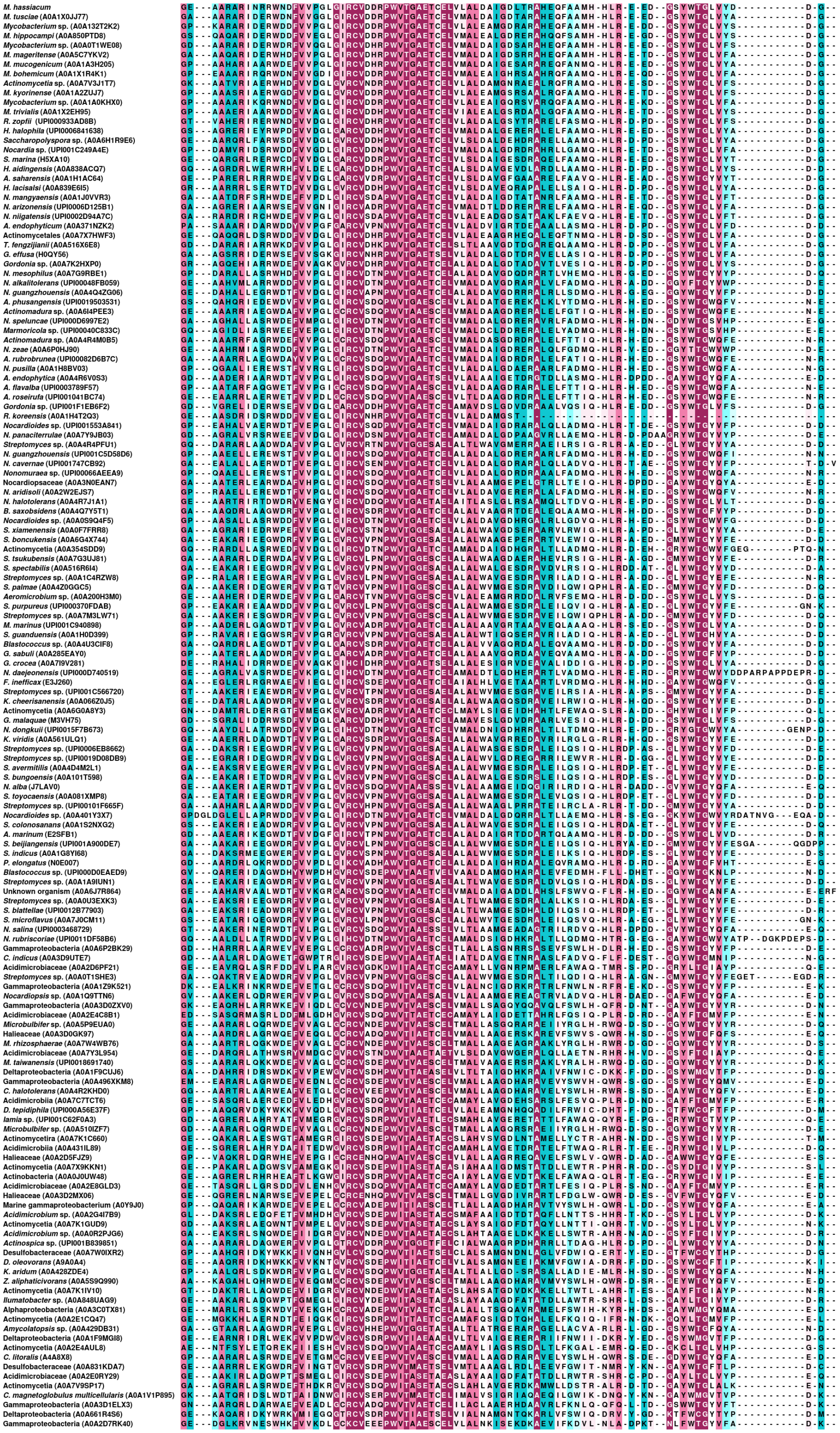

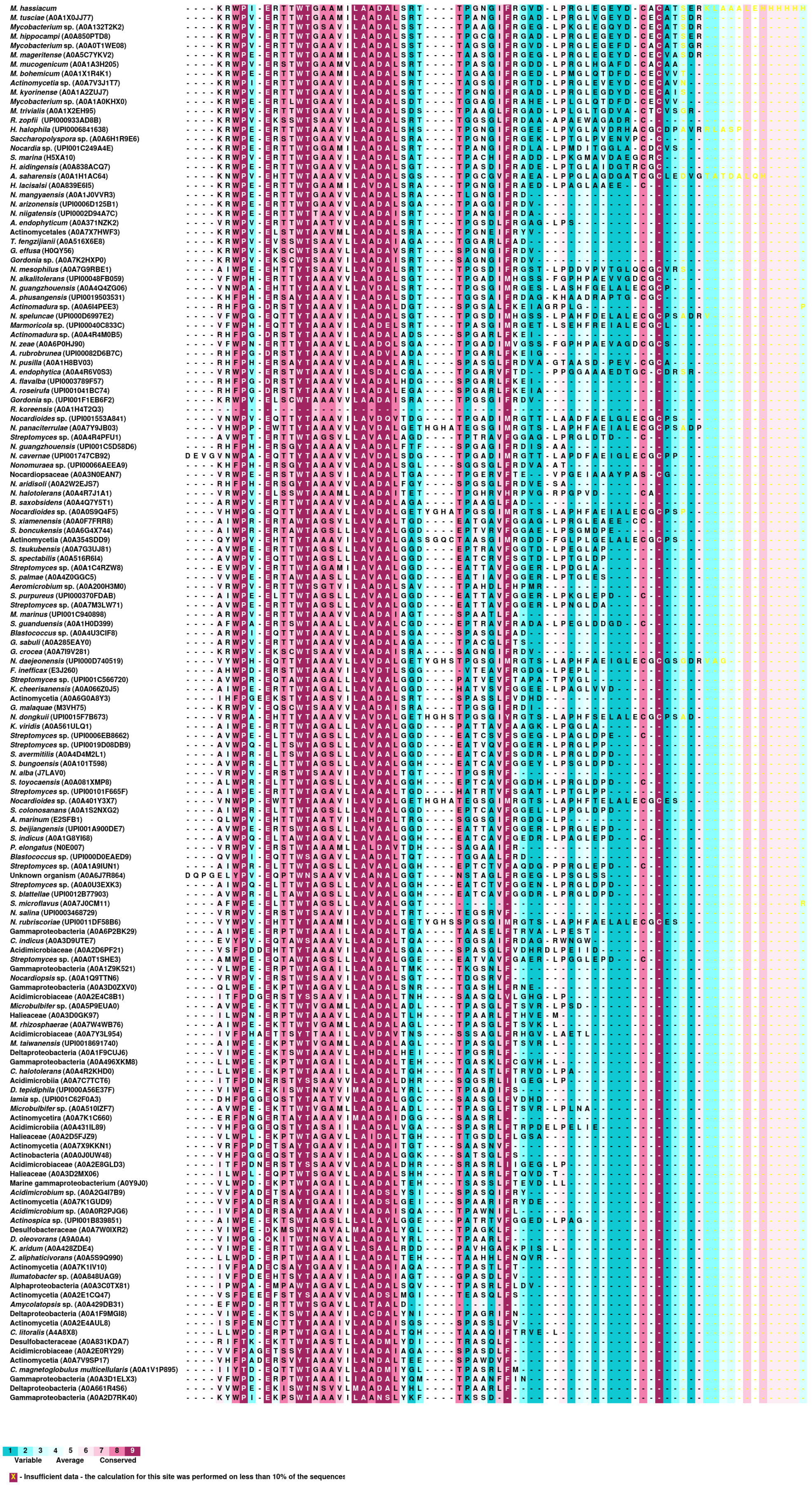
1 2 3 4 5 6 7 8 9
Variable
Average
Conserved
X - Insufficient data - the calculation for this site was performed on less than 10% of the sequences

Fig. S7. ConSurf color-coded sequence alignment for MmpH. The organism name and UniProt entry code are indicated before each sequence. The color-code key is given at the end of the alignment.

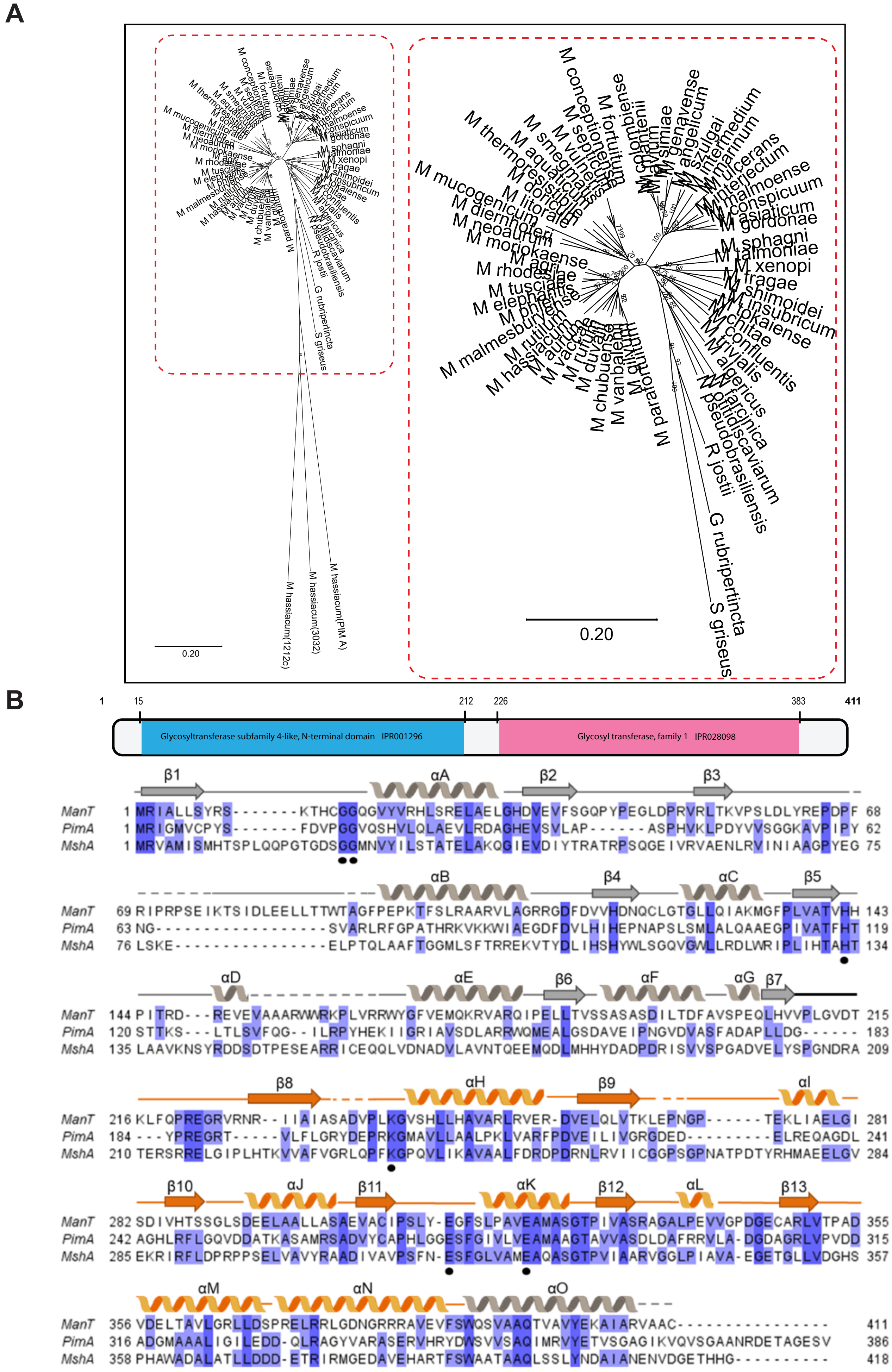
A
0.20
M hassiacum(1212c)
M hassiacum(3032)
M hassiacum(PIM A)
G rubripertincta
S griseus
R jostii
B
1
15
212
226
383
411
Glycosyltransferase subfamily 4-like, N-terminal domain IPR001296
Glycosyl transferase, family 1 IPR028098
ManT
PimA
MshA

Fig. S8. Analysis of the amino acid sequences of ManT and relevant homologues. (**A**) Phylogenetic reconstruction based on 61 ManT amino acid sequences and 3 relevant distant homologues (Table S6) using the Neighbor-Joining method [14]. The optimal tree is drawn to scale and evolutionary distances were computed using the Poisson correction method [15]. The scale bar indicates the number of amino acid substitutions per site. Analyses were performed with MEGA X [16]. (**B**) Representation of protein families and functionally important domains predicted using InterProScan [18] and amino acid sequence alignment of ManT with two representative GT-B fold members. The amino acid sequences of ManT from *M. hassiacum*, PimA from *M. smegmatis* and MshA from *Corynebacterium glutamicum* were aligned using Clustal Omega [21] and represented with Jalview [22]. Strictly conserved amino acids are represented in dark blue boxes. The secondary structure elements of ManT are represented above the alignment and colored grey (N-terminal domain) or orange (C-terminal domain). Dashed lines represent the segments excluded from the atomic model of ManT. The proposed GPGTF/GT-B superfamily signature residues are indicated by black dots.

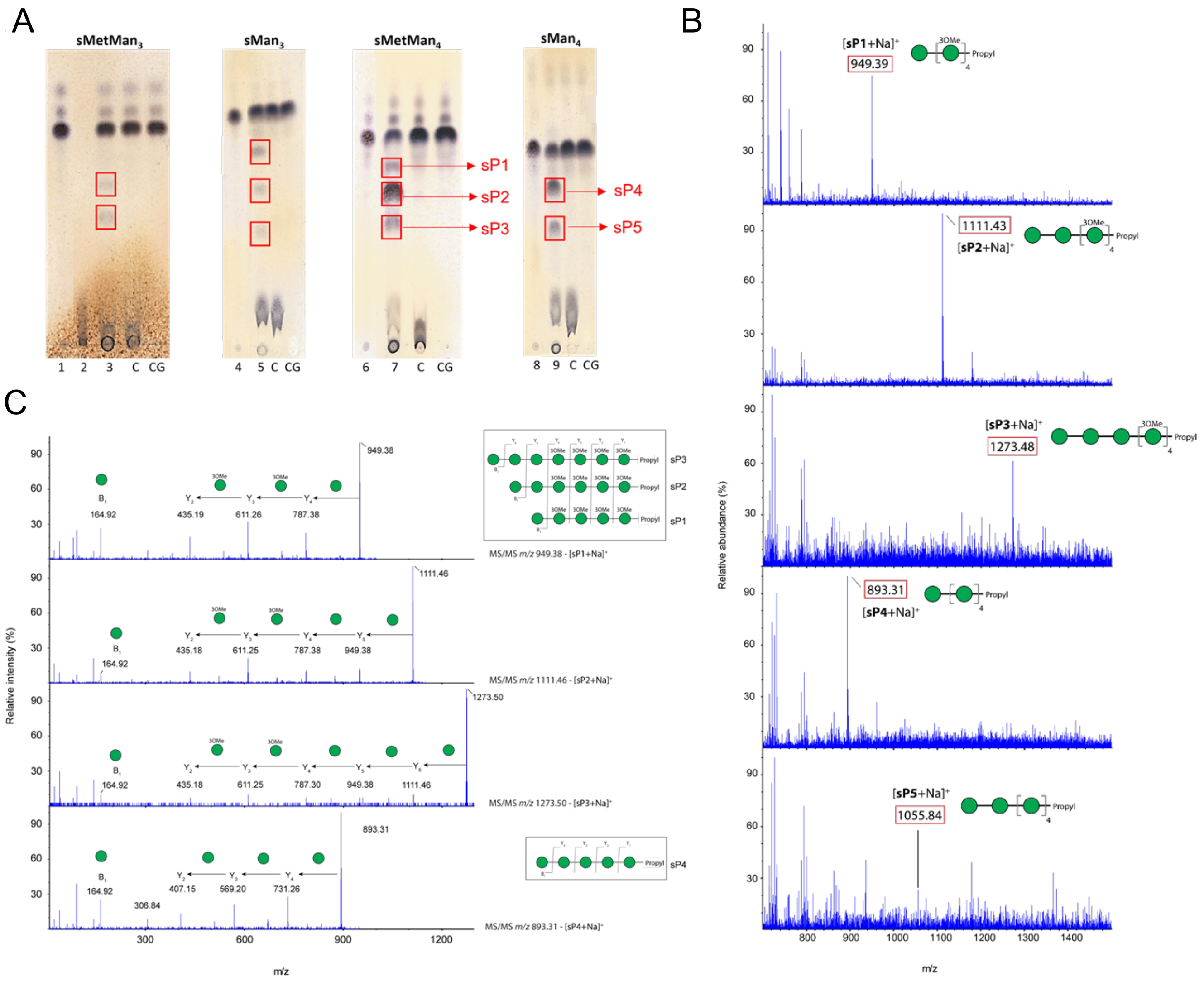

Fig. S9. Analysis of ManT activity. (**A**) TLC analysis of the activity of ManT with chemically synthesized substrates. Lane 1, sMetMan3 standard; lane 2, GDP-Man; lane 3, reaction with sMetMan3; lane 4, sMetMan4 standard; lane 5, reaction with sMetMan4; lane 6, sMan3 standard; lane 7, reaction with sMan3; lane 8, sMan4 standard; lane 9, reaction with sMan4; C, Control reaction without enzyme; CG, Control reaction without GDP-Man. ManT products (sP1 to sP5) are indicated in red boxes. (**B**) MS and (**C**) MS/MS analysis of ManT products with synthetic tetramannosides as substrates. ESI-TOF spectra were acquired in positive ion mode. The $[M+Na]^+$ ions are identified in red boxes. Product ion nomenclature follows that proposed by Domon and Costello [12] and the fragmentation pathways, Y- and B-type glycosidic cleavages are shown.

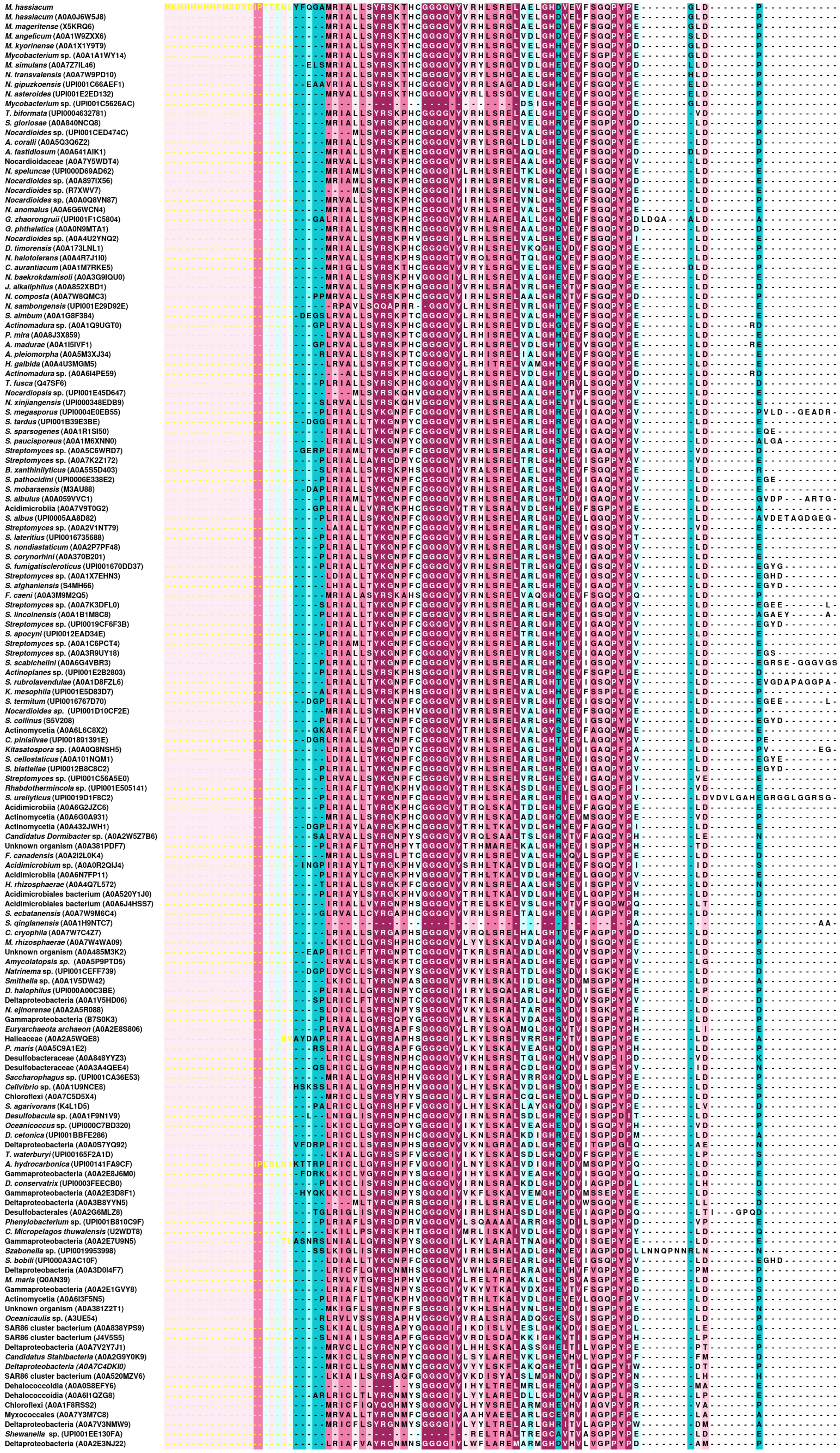

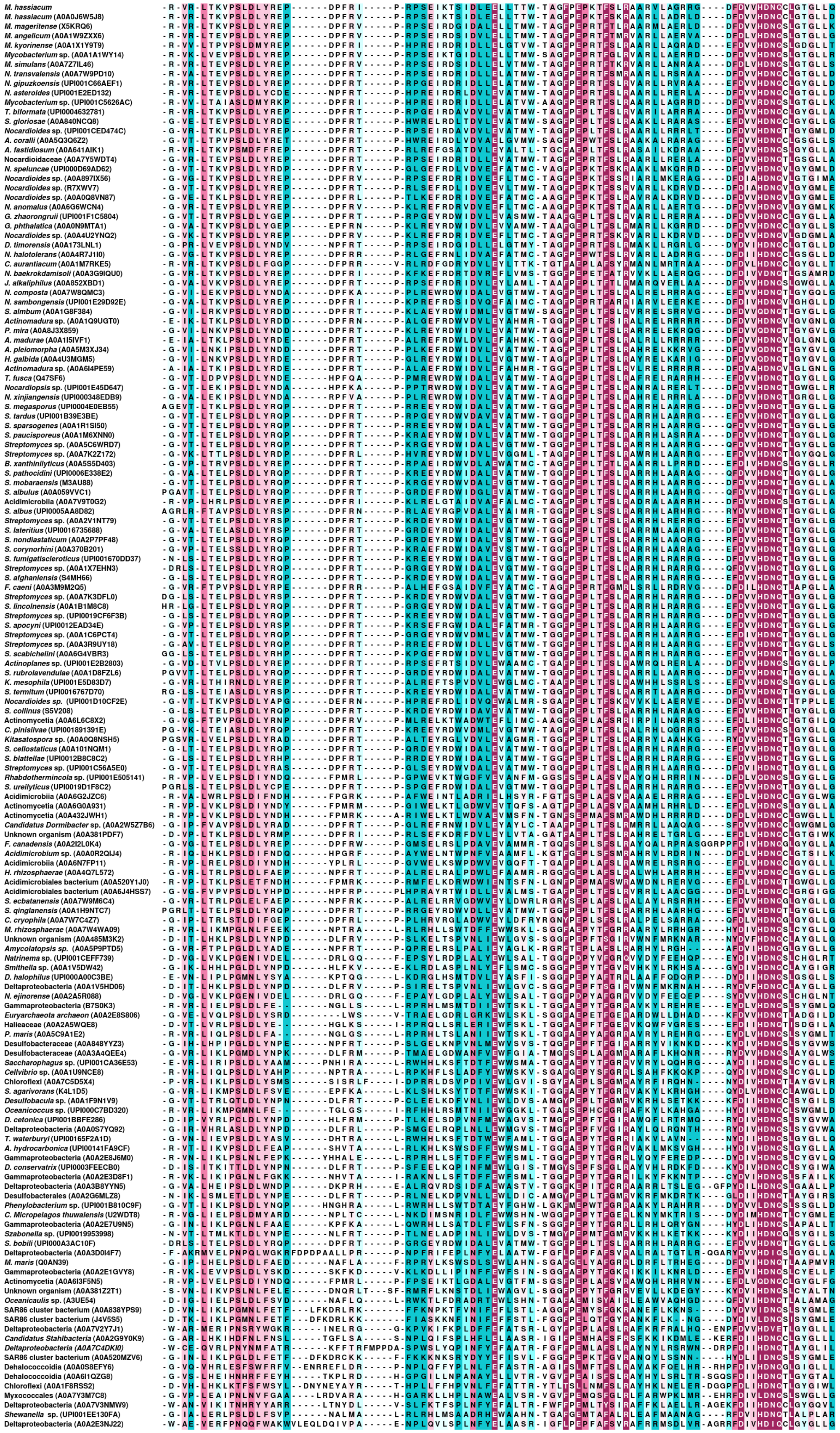

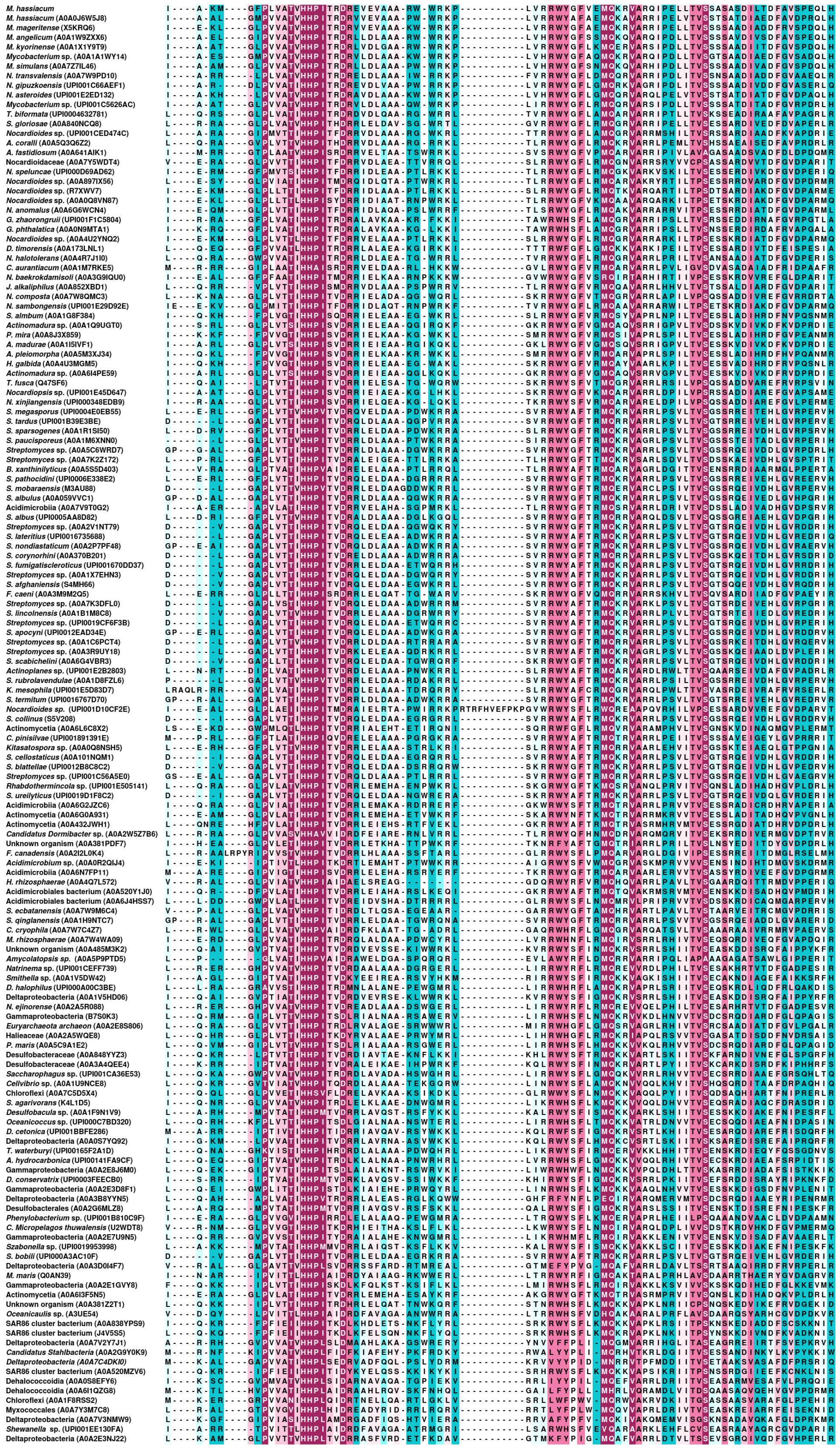

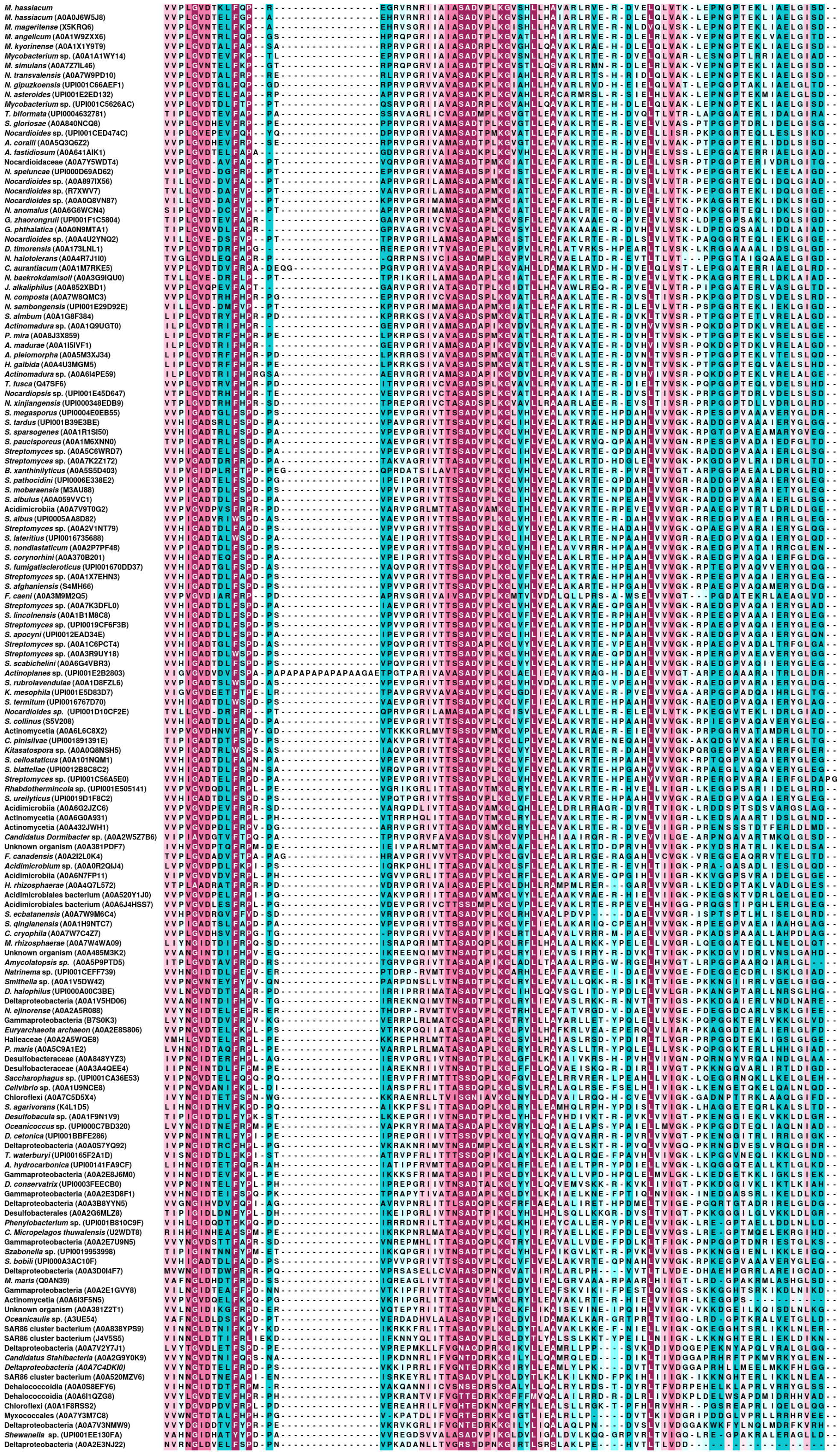

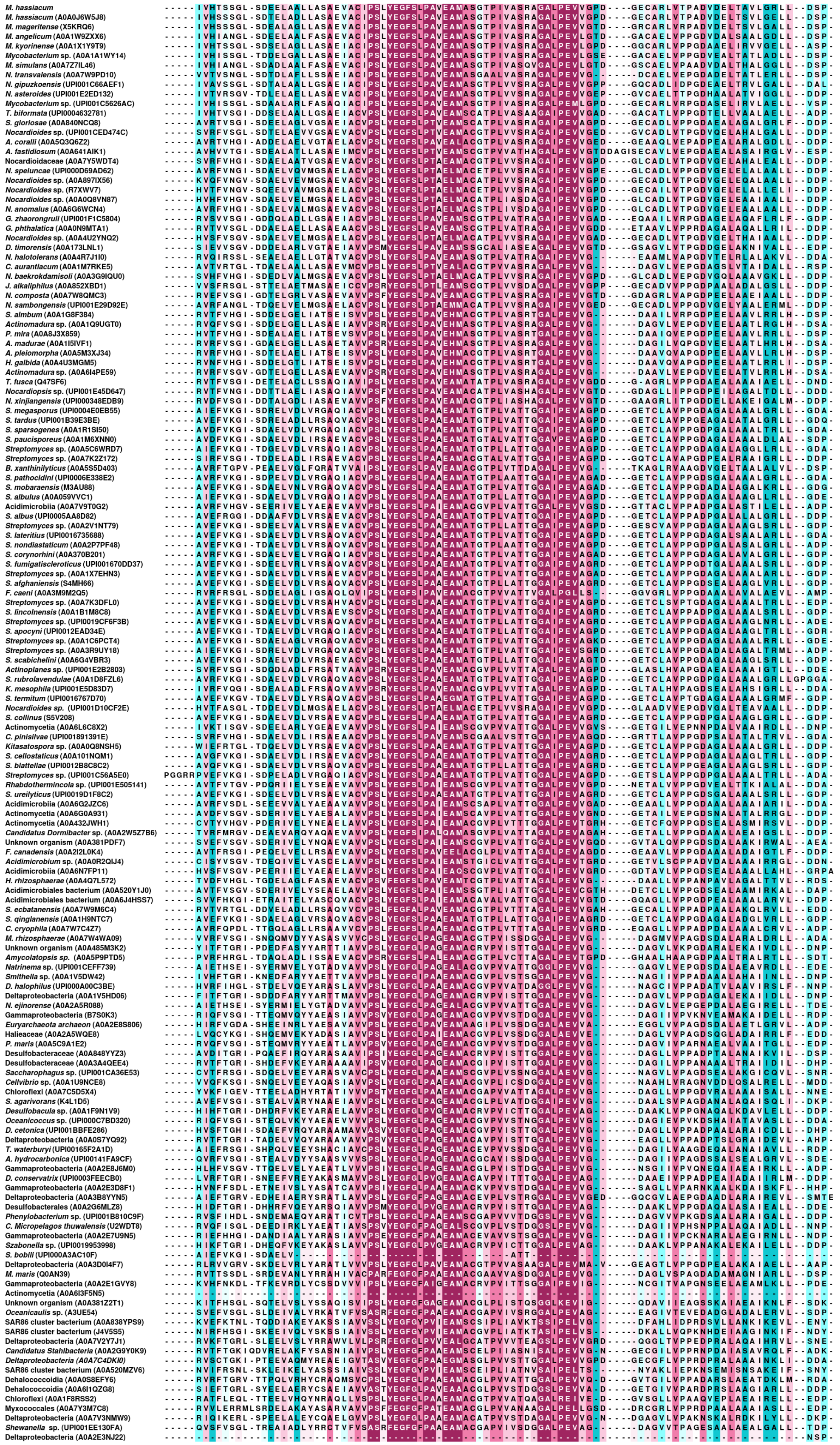

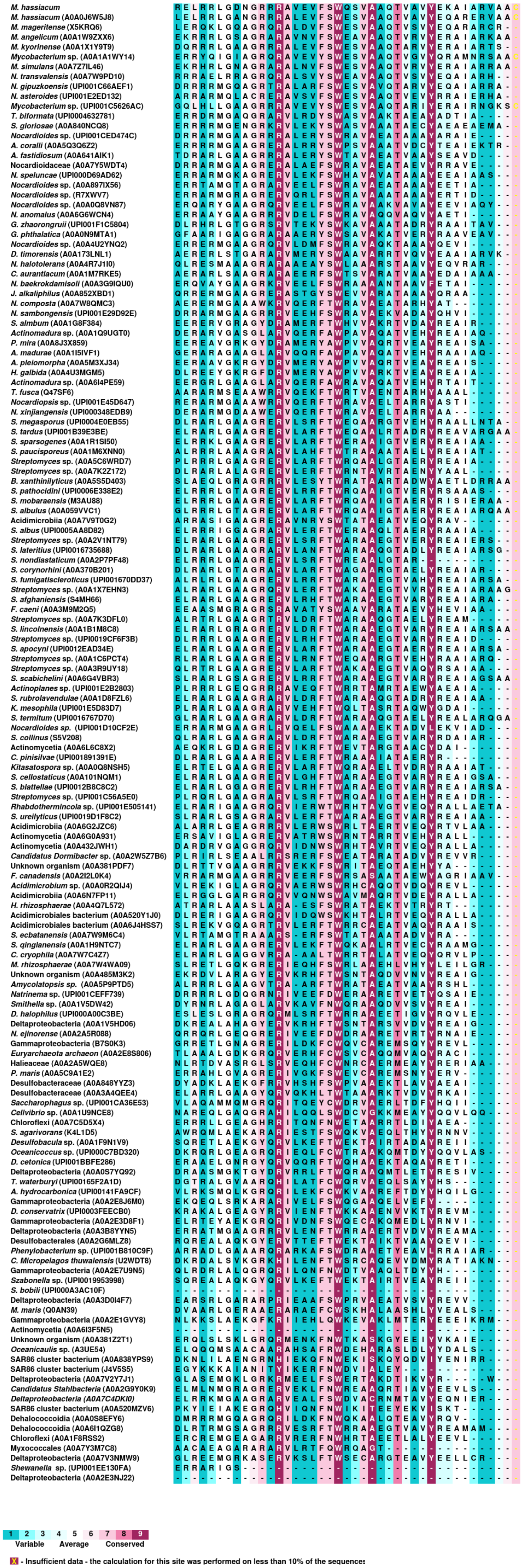
1 2 3 4 5 6 7 8 9
Variable Average Conserved
X - Insufficient data - the calculation for this site was performed on less than 10% of the sequences

Fig. S10. ConSurf color-coded sequence alignment for ManT. The organism name and UniProt entry code are indicated before each sequence. The color-code key is given at the end of the alignment.

A

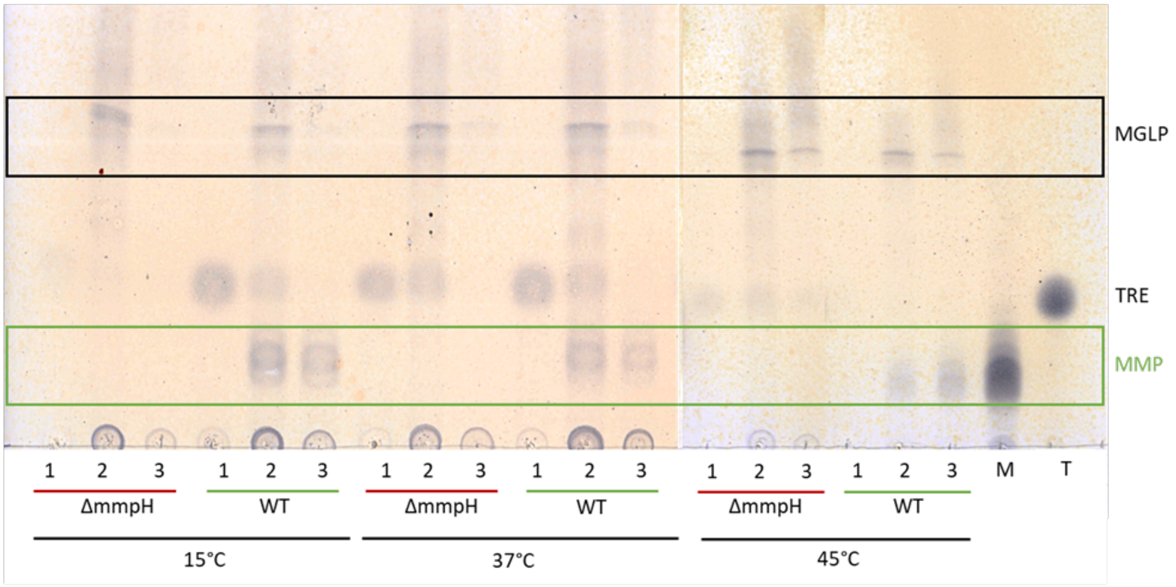

B

15 °C			37 °C			45 °C		
Replicate	Strain	GT (h)	Replicate	Strain	GT (h)	Replicate	Strain	GT (h)
1	mutant	79.7	1	mutant	2.9	1	mutant	2.4
	WT	71.4		WT	2.9		WT	2.4
2	mutant	61.9	2	mutant	2.2	2	mutant	2.1
	WT	45.3		WT	2.2		WT	2.1
3	mutant	64.8	3	mutant	3.0	3	mutant	2.0
	WT	56.8		WT	3.2		WT	2.0

Fig. S11. Effect of MMP absence on the growth rate of ΔmmpH *M. smegmatis* mutant in GBM at different temperatures. (**A**) TLC analysis of PMPS purified from *M. smegmatis* WT and ΔmmpH mutant at different temperatures. Lanes 1, 2 and 3 are fractions eluted with 40, 60 and 80% (v/v) methanol, respectively. M, MMP standard; T (TRE), trehalose standard. MGLP is highlighted by a black box and MMP by a green box. (**B**) Calculated generation times (GT) at the indicated temperatures.

Supplementary Tables

Table S1. Proteomes of actinobacterial species used for phylogenetic tree construction

RefSeq assembly accession	Assembly name	Organism	Strain
GCF_003568625.1	ASM356862v1	*Gordonia rubripertincta*	CWB2
GCF_001653095.1	ASM165309v1	*Hoyosella altamirensis*	NBRC 109631
GCF_001942505.1	ASM194250v1	*Mycobacterium abscessus* subsp. *abscessus*	ATCC 19977
GCF_002553505.1	ASM255350v1	*Mycobacterium agri*	CCUG37673
GCF_010726245.1	ASM1072624v1	*Mycobacterium aichiense*	JCM 6376
GCF_002086455.1	ASM208645v1	*Mycobacterium algericus*	DSM 45454
GCF_002086635.1	ASM208663v1	*Mycobacterium alsense*	DSM 45230
GCF_002086155.1	ASM208615v1	*Mycobacterium angelicum*	DSM 45057
GCF_002086485.1	ASM208648v1	*Mycobacterium aquaticum*	RW6
GCF_000559085.1	ASM55908v2	*Mycobacterium aromaticivorans*	JS19b1
GCF_002086125.1	ASM208612v1	*Mycobacterium arosiense*	DSM 45069
GCF_002086515.1	ASM208651v1	*Mycobacterium arupensis*	DSM 44942
GCF_002086545.1	ASM208654v1	*Mycobacterium asiaticum*	DSM 44297
GCF_900566085.1	LAUMK41	*Mycobacterium attenuatum*	MK41
GCF_010730955.1	ASM1073095v1	*Mycobacterium aubagnense*	JCM 15296
GCF_900637195.1	50279_F01	*Mycobacterium aurum*	NCTC10437
GCF_002968275.1	ASM296827v1	*Mycobacterium austroafricanum* [a]	ITM-2016-00315
GCF_000174035.1	ASM17403v1	*Mycobacterium avium* subsp. *avium*	ATCC 25291
GCF_002086115.1	ASM208611v1	*Mycobacterium bacteremicum*	DSM 45578
GCF_010731295.1	ASM1073129v1	*Mycobacterium boenickei*	JCM 15653
GCF_002102025.1	ASM210202v1	*Mycobacterium bohemicum*	DSM 44277
GCF_002086165.1	ASM208616v1	*Mycobacterium bouchedurhonense*	DSM 45439
GCF_002086575.1	ASM208657v1	*Mycobacterium branderi*	DSM 44624
GCF_001570425.1	ASM157042v1	*Mycobacterium brisbanense*	JCM15654
GCF_002553575.1	ASM255357v2	*Mycobacterium brumae*	CIP1034565
GCF_002101555.1	ASM210155v1	*Mycobacterium canariasense*	CCUG 47953
GCF_000253375.1	ASM25337v1	*Mycobacterium canettii*	CIPT 140010059
GCF_002101595.1	ASM210159v1	*Mycobacterium celatum*	DSM 44243
GCF_010731795.1	ASM1073179v1	*Mycobacterium celeriflavum*	JCM 18439
GCF_001632805.1	ASM163280v1	*Mycobacterium chelonae*	CCUG 47445
GCF_002219285.1	ASM221928v1	*Mycobacterium chimaera*	DSM 44623
GCF_900637205.1	50279_G01	*Mycobacterium chitae*	NCTC10485
GCF_001552315.1	ASM155231v1	*Mycobacterium chlorophenolicum*	NBRC 15527
GCF_002086595.1	ASM208659v1	*Mycobacterium chubuense*	DSM 44219
GCF_002105755.1	ASM210575v1	*Mycobacterium colombiense*	CECT 3035
GCF_002102065.1	ASM210206v1	*Mycobacterium conceptionense*	CCUG 50187

GCF_010729895.1	ASM1072989v1	*Mycobacterium confluentis*	JCM 13671
GCF_010730195.1	ASM1073019v1	*Mycobacterium conspicuum*	JCM 14738
GCF_005670675.1	ASM567067v1	*Mycobacterium cosmeticum*	DSM 44829
GCF_002104675.1	ASM210467v1	*Mycobacterium decipiens*	TBL 1200985
GCF_002553495.1	ASM255349v1	*Mycobacterium diernhoferi*	IP141170001
GCF_010728155.1	ASM1072815v1	*Mycobacterium doricum*	JCM 12405
GCF_010726645.1	ASM1072664v1	*Mycobacterium duvalii*	JCM 6396
GCF_004354905.1	ASM435490v1	*Mycobacterium eburneum*	DSM 44358
GCF_002086605.1	ASM208660v1	*Mycobacterium elephantis*	FI-09383
GCF_002101585.1	ASM210158v1	*Mycobacterium engbaekii*	ATCC 27353
GCF_002102155.1	ASM210215v1	*Mycobacterium europaeum*	DSM 45397
GCF_010726955.1	ASM1072695v1	*Mycobacterium fallax*	JCM 6405
GCF_000723385.1	PRJEB5746_assembly_1	*Mycobacterium farcinogenes*	DSM 43637
GCF_002101635.1	ASM210163v1	*Mycobacterium florentinum*	DSM 44852
GCF_011758805.1	ASM1175880v1	*Mycobacterium fluoranthenivorans*	DSM 44556
GCF_001307545.1	ASM130754v1	*Mycobacterium fortuitum*	CT6
GCF_002102185.1	ASM210218v1	*Mycobacterium fragae*	DSM 45731
GCF_004355025.1	ASM435502v1	*Mycobacterium franklinii*	DSM 45524
GCF_002102175.1	ASM210217v1	*Mycobacterium gastri*	DSM 43505
GCF_000526915.1	ASM52691v1	*Mycobacterium genavense*	ATCC 51234
GCF_000184435.1	ASM18443v1	*Mycobacterium gilvum*	Spyr1
GCF_001187505.1	ASM118750v1	*Mycobacterium goodii*	X7B
GCF_002101675.1	ASM210167v1	*Mycobacterium gordonae*	DSM 44160
GCF_008329645.1	ASM832964v1	*Mycobacterium grossiae*	DSM 104744
GCF_000340435.2	ASM34043v3	*Mycobacterium haemophilum*	ATCC 29548
GCF_000300375.1	ASM30037v1	*Mycobacterium hassiacum*	DSM 44199
GCF_001077755.1	ASM107775v1	*Mycobacterium heckeshornense*	RLE
GCF_010730745.1	ASM1073074v1	*Mycobacterium heidelbergense*	JCM 14842
GCF_001021505.1	ASM102150v1	*Mycobacterium heraklionensis*	Davo
GCF_010729485.1	ASM1072948v1	*Mycobacterium hiberniae*	JCM 13571
GCF_001722355.1	ASM172235v1	*Mycobacterium holsaticum*	M7
GCF_900078665.2	PRJEB13221	*Mycobacterium houstonense*	ATCC 49403
GCF_002101665.1	ASM210166v1	*Mycobacterium immunogenum*	ATCC 700505
GCF_900566055.1	LAUMK13	*Mycobacterium innocens*	MK13
GCF_010731615.1	ASM1073161v1	*Mycobacterium insubricum*	JCM 16366
GCF_900078675.2	PRJEB13236	*Mycobacterium interjectum*	ATCC 51457
GCF_002086275.1	ASM208627v1	*Mycobacterium intermedium*	DSM 44049
GCF_000277125.1	ASM27712v1	*Mycobacterium intracellulare*	ATCC 13950
GCF_002101705.1	ASM210170v1	*Mycobacterium iranicum*	DSM 45541
GCF_000157895.3	ASM15789v2	*Mycobacterium kansasii*	ATCC 12478
GCF_001050035.1	Mycobacterium komanii GPK 1020	*Mycobacterium komanii*	GPK 1020
GCF_010731835.1	ASM1073183v1	*Mycobacterium koreensis*	JCM 19956

GCF_002101745.1	ASM210174v1	*Mycobacterium kubicae*	CIP 106428
GCF_002086285.1	ASM208628v1	*Mycobacterium kumamotonensis*	DSM 45093
GCF_003254575.1	ASM325457v1	*Mycobacterium kyogaense*	NCTC 11659
GCF_002101735.1	ASM210173v1	*Mycobacterium kyorinense*	DSM 45166
GCF_002102215.1	ASM210221v1	*Mycobacterium lacus*	DSM 44577
GCF_002245535.1	ASM224553v1	*Mycobacterium lehmannii*	CECT 8763
GCF_001373395.1	PRJEB8430_assembly_1	*Mycobacterium lentiflavum*	CSUR P1491
GCF_000195855.1	ASM19585v1	*Mycobacterium leprae TN*	TN
GCF_002291465.1	ASM229146v1	*Mycobacterium lepraemurium*	Hawaii
GCF_010731695.1	ASM1073169v1	*Mycobacterium litorale*	JCM 17423
GCF_000878195.1	ASM87819v1	*Mycobacterium llatzerense*	CLUC14
GCF_002102265.1	ASM210226v1	*Mycobacterium longobardus*	DSM 45394
GCF_010727475.1	ASM1072747v1	*Mycobacterium mageritense*	JCM 12375
GCF_001050015.1	Mycobacterium malmesburii WCM 7299	*Mycobacterium malmesburyense*	WCM 7299
GCF_001686735.1	ASM168673v1	*Mycobacterium malmoense*	E1298
GCF_002086335.1	ASM208633v1	*Mycobacterium mantenii*	DSM 45255
GCF_000723425.2	E11	*Mycobacterium marinum*	E11
GCF_002086345.1	ASM208634v1	*Mycobacterium marseillense*	DSM 45437
GCF_002086405.1	ASM208640v1	*Mycobacterium minnesotensis*	DSM 45633
GCF_010731575.1	ASM1073157v1	*Mycobacterium monacense*	JCM 15658
GCF_003112775.1	ASM311277v1	*Mycobacterium montefiorense*	BS
GCF_010726085.1	ASM1072608v1	*Mycobacterium moriokaense*	JCM 6375
GCF_001668705.1	ASM166870v1	*Mycobacterium mucogenicum*	1127319.6
GCF_004359045.1	ASM435904v1	*Mycobacterium mucogenicum*	24AIII
GCF_001291445.1	Mycobacterium mucogenicum	*Mycobacterium mucogenicum*	CSUR P2099
GCF_002102255.1	ASM210225v1	*Mycobacterium nebraskense*	DSM 44803
GCF_000691525.1	ATCC25795_good_1 (paired) assembly	*Mycobacterium neoaurum*	ATCC 25795
GCF_001245615.1	Mycobacterium neworleansense assembly 1	*Mycobacterium neworleansense*	ATCC 49404
GCF_002101775.1	ASM210177v1	*Mycobacterium nonchromogenicus*	DSM 44164
GCF_002086415.1	ASM208641v1	*Mycobacterium noviomagense*	DSM 45145
GCF_001570485.1	ASM157048v1	*Mycobacterium novocastrense*	JCM18114
GCF_001044245.1	ASM104424v1	*Mycobacterium obuense*	DSM 44075
GCF_002101785.1	ASM210178v1	*Mycobacterium palustre*	DSM 44572
GCF_002101815.1	ASM210181v1	*Mycobacterium paraense*	IEC26
GCF_001907675.1	ASM190767v1	*Mycobacterium paraffinicum*	M11
GCF_010725485.1	ASM1072548v1	*Mycobacterium parafortuitum*	JCM 6367
GCF_003614435.1	ASM361443v1	*Mycobacterium paragordonae*	49061
GCF_002104735.1	ASM210473v1	*Mycobacterium paraintracellulare*	KCTC 29084
GCF_000164135.1	ASM16413v1	*Mycobacterium parascrofulaceum*	ATCC BAA-614
GCF_010731655.1	ASM1073165v1	*Mycobacterium paraseoulense*	JCM 16952

GCF_002102335.1	ASM210233v1	*Mycobacterium parmense*	DSM 44553
GCF_002102345.1	ASM210234v1	*Mycobacterium peregrinum*	DSM 43271
GCF_002086675.1	ASM208667v1	*Mycobacterium persicum*	AFPC-000227
GCF_001582005.1	ASM158200v1	*Mycobacterium phlei*	DSM 43070
GCF_010731115.1	ASM1073111v1	*Mycobacterium phocaicum*	JCM 15301
GCF_002086835.1	ASM208683v1	*Mycobacterium porcinum*	IP141460001
GCF_900566075.1	MK142	*Mycobacterium pseudokansasii*	MK142
GCF_003584745.1	ASM358474v1	*Mycobacterium pseudoshottsii*	JCM 15466
GCF_002086695.1	ASM208669v1	*Mycobacterium rhodesiae*	DSM 44223
GCF_002101845.1	ASM210184v1	*Mycobacterium riyadhense*	DSM 45176
GCF_900108565.1	IMG-taxon 2636415969 annotated assembly	*Mycobacterium rutilum*	DSM 45405
GCF_004924335.1	ASM492433v1	*Mycobacterium salmoniphilum*	DSM 43276
GCF_002086715.1	ASM208671v1	*Mycobacterium saopaulense*	CCUG 66554
GCF_010729105.1	ASM1072910v1	*Mycobacterium saskatchewanense*	JCM 13016
GCF_002086735.1	ASM208673v1	*Mycobacterium scrofulaceum*	DSM 43992
GCF_001012795.1	ASM101279v1	*Mycobacterium senegalense*	CK1
GCF_002101885.1	ASM210188v1	*Mycobacterium senuensis*	DSM 44999
GCF_012396425.1	ASM1239642v1	*Mycobacterium septicum*	ATCC 700731
GCF_000805385.1	ASM80538v1	*Mycobacterium setense*	DSM 45070
GCF_002102355.1	ASM210235v1	*Mycobacterium sherrisii*	ATCC BAA-832
GCF_002356315.1	ASM235631v1	*Mycobacterium shigaense*	JCM 32072
GCF_900417275.1	PRJEB26812	*Mycobacterium shimoidei*	P7336
GCF_010730055.1	ASM1073005v1	*Mycobacterium shinjukuense*	JCM 14233
GCF_010727605.1	ASM1072760v1	*Mycobacterium simiae*	JCM 12377
GCF_001457595.1	NCTC8159	*Mycobacterium smegmatis*	NCTC8159
GCF_002250655.1	ASM225065v1	*Mycobacterium sphagni*	ATCC 33027
GCF_002356335.1	ASM235633v1	*Mycobacterium stephanolepidis*	NJB0901
GCF_001942625.1	ASM194262v1	*Mycobacterium syngnathidarum*	27335
GCF_002116635.1	ASM211663v1	*Mycobacterium szulgai*	DSM 44166
GCF_002967005.1	ASM296700v1	*Mycobacterium talmoniae*	ATCC BAA-2683
GCF_002101955.1	ASM210195v1	*Mycobacterium terrae*	CIP 104321
GCF_000234585.1	ASM23458v1	*Mycobacterium thermoresistibile*	ATCC 19527
GCF_010723675.1	ASM1072367v1	*Mycobacterium timonense*	JCM 30726
GCF_010725885.1	ASM1072588v1	*Mycobacterium tokaiense*	JCM 6373
GCF_002102415.1	ASM210241v1	*Mycobacterium triplex*	DSM 44626
GCF_002102395.1	ASM210239v1	*Mycobacterium trivialis*	DSM 44153
GCF_000195955.2	ASM19595v2	*Mycobacterium tuberculosis*	H37Rv
GCF_002086795.1	ASM208679v1	*Mycobacterium tusciae*	DSM 44338
GCF_000013925.1	ASM1392v2	*Mycobacterium ulcerans* [b]	Agy99
GCF_002355775.1	ASM235577v1	*Mycobacterium ulcerans* subsp. *shinshuense* [b]	ATCC 33728
GCF_000295825.1	ASM29582v1	*Mycobacterium vaccae*	ATCC 25954
GCF_000015305.1	ASM1530v1	*Mycobacterium vanbaalenii* [a]	PYR-1

GCF_002967035.1	ASM296703v1	*Mycobacterium virginiensis*	GF75
GCF_002104765.1	ASM210476v1	*Mycobacterium vulneris*	DSM 45247
GCF_002101965.1	ASM210196v1	*Mycobacterium wolinskyi*	ATCC 700010
GCF_009936235.1	ASM993623v1	*Mycobacterium xenopi*	JCM 15661T
GCF_000250675.2	ASM25067v3	*Nocardia brasiliensis*	HUJEG-1
GCF_001182745.1	NCTC11134	*Nocardia farcinica*	NCTC11134
GCF_007362295.1	ASM736229v1	*Nocardia otitidiscaviarum*	NEB252
GCF_001613105.1	ASM161310v1	*Nocardia pseudobrasiliensis*	NBRC 108224
GCF_000014565.1	ASM1456v1	*Rhodococcus jostii*	RHA1
GCF_000010605.1	ASM1060v1	*Streptomyces griseus*	NBRC 13350

[a] - for ease of representation collapsed into *M. vanbaalenii* branch

[b] - for ease of representation collapsed into *M. ulcerans* branch

Table S2. Average Nucleotide Identity (ANI) and Genome to Genome Distance (GGD) pairwise values

Query genome	Reference genome	OrthoANIu value (%)	GGD F2 (%)
24A III	*M. mucogenicum* CSUR_P2099	92.07	45.8
24A III	*M. mucogenicum* 1127319	92.12	46.2
24A III	*M. mucogenicum* DSM 44124	92.01	45.9
24A III	*M. phocaicum* JCM 15301	95.27	62.0
24A III	*M. phocaicum* DSM 45104	95.25	61.9
CSUR_P2099	*M. mucogenicum* 1127319	96.40	73.5
JCM_15301	*M. phocaicum DSM* 45104	99.93	99.9

The pairwise values were calculated with the OrthoANIu algorithm (https://www.ezbiocloud.net/tools/ani) [23] and the Genome to Genome Distance Calculator 2.1 (http://ggdc.dsmz.de/ggdc.php#) [24] using the Formula 2 option. Taxa pairs with ANI>95% or a GGD>70% were considered to belong to the same species. Taxa with ANI≤97% and GGD≤80% were considered subspecies [25]. The *M. phocaicum* strain 24A III (RGM lacking the MMP cluster) used to probe for the presence of MMP had been initially identified as *M. mucogenicum* based on partial 16s rRNA, *rpoB* and *hsp65* genes [26]. However, the recently completed genome sequence [27] and phylogenetic analyses based on a comparison of ANI and GGD values to those of *M. phocaicum* strains (GCA_010731115.1 and GCA_005670655.1) revealed that it likely represents a subspecies of *M. phocaicum* [28, 29].

Table S3. Oligonucleotides used in this work

Code	Name	Sequence (5’-3’)
V1	D47A_F	GCGGTCATACCGCACCGTGGGACCAC
V2	D47A_R	CGCCAGTATGGCGTGGCACCCTGGTG
V3	D50A_F	ACCGATCCGTGGGCACACGTTGAAAAC
V4	D50A_R	TGGCTAGGCACCCGTGTGCAACTTTTG
V5	E262A_F	TGACCGGTGCAGCAACCTGTGAACTGG
V6	E262A_R	ACTGGCCACGTCGTTGGACATTGACC
A1	82F_KpnI	ATA**GGTACC**ATGAGCCATGCCGACACCGCACT
A2	81R_BamHI	ACGGATCCTATTGGACCGGACTGGTTTTCGC
A3	81F_BamHI	ATA**GGATCC**AACGCCTCCGGATGCTCGG
A4	83R_HindIII	ATT**AAGCTT**GTGCTGACAGTCGACTTCGACCGG
A5	MF1R	CGACGGATCGTGCGCACGGCTG
A6	MF2F	TCTGCAGGCATTGTTCGGGCGTCAG
A7	MF3R	AGTCCATCGCGGCCACCCAGGAG
A8	MF4F	CCCCGTTGCGGTTCCTGTTCATCG

The restriction sites used are underlined, with those inserted for genetic manipulation in bold type.

Table S4. Data collection and refinement statistics [a]

Dataset	MmpH (native)	MmpH (Se-Met)	ManT
**Data Collection**			
Source	ALBA BL13-XALOC	ESRF BM30A	ALBA BL13-XALOC
Wavelength (Å)	0.9809	0.9799	1.0332
Resolution range (Å)	148.54 - 1.35 (1.42 - 1.35)	49.10 - 2.01 (2.12 - 2.01)	66.59 - 2.75 (2.88 - 2.75)
Space group	$P2_1$	$P2_12_12_1$	C2
Unit cell dimensions	a=47.0 Å b=51.8 Å c=149.1 Å β=95.1°	a=51.9 Å b=91.6 Å c=152.8 Å	a=136.3 Å b=77.4 Å c=101.2Å β=106.5°
Reflections (measured/unique)	1,020,715 / 157,006 (135,908 / 22,871)	628,335 / 47,481 (48,811 / 5,525)	92,036 / 26,403 (12,268 / 3,487)
Multiplicity	6.5 (5.9)	13.2 (8.7)	3.5 (3.5)
Completeness (%)	99.9 (100.0)	96.1 (78.4)	99.8 (99.6)
Mean (I) / σ (I)	16.4 (1.3)	10.0 (2.3)	9.8 (1.50)
Wilson B-factor	16.2	20.5	83.5
R-merge	0.055 (1.323)	0.176 (0.844)	0.063 (0.945)
CC 1/2	1 (0.588)	0.998 (0.881)	0.997 (0.668)
Monomers per asymmetric unit	2	2	2
Matthews coefficient ($Å^3$ Da^{-1})	2.20	2.20	2.65
Solvent content (%)	44.0	44.2	53.7
**Refinement**			
R-work	0.1424 (0.3202)		0.2706 (0.3772)
R-free	0.1768 (0.3541)		0.2867 (0.3818)
Number of atoms:			
non-hydrogen	6,211		4,801
macromolecules	5,491		4,801
ligands	87		0
water	681		0
Protein residues	691		683

RMSD bonds (Å)	0.005		0.002
RMSD angles (°)	0.73		0.49
Ramachandran favored (%)	98.8		93.9
Ramachandran outliers (%)	0		0
Clashscore	2.03		3.99
B-factor:			
average	23.4		102.6
macromolecules	21.6		102.6
ligands	47.8		-
solvent	36.7		-
PDB entry	7QSJ		7QSG
SBGrid Data Bank entry	https://doi.org/10.15785/SBGRID/874	https://doi.org/10.15785/SBGRID/875	https://doi.org/10.15785/SBGRID/873

[a] Values in parenthesis correspond to the highest resolution shell.

Table S5. Protein sequences used to build the MmpH phylogenetic tree shown in Fig. S3

Accession	Organism	Accession	Organism	Accession	Organism
WP_005201451.1	*G. rubripertincta*	WP_011892000.1	*M. gilvum*	WP_113964144.1	*M. shimodei*
WP_097943299.1	*M. agri*	WP_069433854.1	*M. gordonae*	WP_044506088.1	*M. simiae*
WP_083035905.1	*M. algericus*	VCT92674.1	*M. hassiacum*	AFP42736.1	*M. smegmatis*
WP_083112734.1	*M. angelicum*	WP_083030981.1	*M. insubricum*	WP_094484562.1	*M. sphagni*
WP_095533839.1	*M. aquaticum*	WP_066912851.1	*M. interjectum*	WP_085671962.1	*M. szulgai*
WP_065120663.1	*M. asiaticum*	WP_069419969.1	*M. intermedium*	WP_071021972.1	*M. talmoniae*
WP_048630751.1	*M. aurum*	WP_163687739.1	*M. litorale*	WP_003927812.1	*M. thermoresistibile*
QGW31891.1	*M. avium*	WP_064496876	*M. jannaschii(TGA)*	WP_115282045.1	*M. tokaiense*
WP_126334007.1	*M. chitae*	WP_090346317.1	*M. malmesburyense*	WP_085111029.1	*M. trivialis*
WP_014818213.1	*M. chubuense*	WP_065446796.1	*M. malmoense*	WP_148685145.1	*M. tusciae*
WP_007771157.1	*M. colombiense*	WP_083092518.1	*M. mantenii*	WP_096370737.1	*M. ulcerans*
WP_165763094.1	*M. conceptionense*	CDM76990.1	*M. marinum*	EJZ10724.1	*M. vaccae*
WP_109788403.1	*M. confluentis*	WP_110314255.1	*M. moriokaense*	ABM16481.1	*M. vanbaalenii*
WP_085231021.1	*M. conspicuum*	WP_064980928.1	*M. mucogenicum*	CDO29253.1	*M. vulneris*
WP_073859553.1	*M. diernhoferi*	WP_030137593.1	*M. neoaurum*	WP_039890256.1	*M. xenopi*
WP_085188183.1	*M. doricum*	WP_083146228.1	*M. parafortuitum*	PFX04803.1	*N. farcinica*
WP_098004287.1	*M. duvalii*	EID09467.1	*M. phlei*	WP_081873014.1	*N. otitidiscaviarum*
RWA20409.1	*M. elephantis*	AEV73050.1	*M. rhodesiae*	WP_067998058.1	*N. pseudobrasiliensis*
WP_165613916.1	*M. fortuitum*	KGI70338.1	*M. rufum*	WP_073358185.1	*R. jostii*
WP_085199919.1	*M. fragae*	WP_083410381.1	*M. rutilum*	WP_030754970.1	*S. griseus*
WP_025737205.1	*M. genavense*	WP_044518968.1	*M. septicum*	BAA97041	*T. vulgaris(TGA)*

Table S6. Protein sequences used to build the ManT phylogenetic tree shown in Fig. S8

Accession	Organism	Accession	Organism	Accession	Organism
WP_143932442.1	*G. rubripertincta*	WP_069433853.1	*M. gordonae*	WP_113964975.1	*M. shimoidei*
WP_097943295.1	*M. agri*	WP_005631138.1	*M. hassiacum*	WP_061555791.1	*M. simiae*
WP_083036126.1	*M. algericus*	WP_018353810	*M. hassiacum 1212c*	ABK71966.1	*M. smegmatis*
WP_083112736.1	*M. angelicum*	WP_085977480	*M. hassiacum 3032*	WP_094484560.1	*M. sphagni*
WP_083170427.1	*M. aquaticum*	WP_018353659	*M. hassiacum PIM A*	WP_085671960.1	*M. szulgai*
WP_065145365.1	*M. asiaticum*	WP_083031008.1	*M. insubricum*	OHV05893.1	*M. talmoniae*
WP_048630753.1	*M. aurum*	ORV85870.1	*M. interjectum*	WP_003927810.1	*M. thermoresistibile*
ETB16456.1	*M. avium*	WP_069419971.1	*M. intermedium*	WP_115280870.1	*M. tokaiense*
WP_126334005.1	*M. chitae*	WP_134057371.1	*M. litorale*	WP_085111031.1	*M. trivialis*
WP_014818215.1	*M. chubuense*	WP_090346320.1	*M. malmesburyense*	WP_006242395.1	*M. tusciae*
WP_065056813.1	*M. colombiense*	WP_065483121.1	*M. malmoense*	BAV41499.1	*M. ulcerans*
WP_064894206.1	*M. conceptionense*	WP_083092513.1	*M. mantenii*	EJZ10726.1	*M. vaccae*
WP_085151102.1	*M. confluentis*	CDM76992.1	*M. marinum*	ABM16483.1	*M. vanbaalenii*
WP_085231023	*M. conspicuum*	WP_114740629.1	*M. moriokaense*	CDO29255.1	*M. vulneris*
WP_073859552.1	*M. diernhoferi*	WP_064980932.1	*M. mucogenicum*	EID13209.1	*M. xenopi*
WP_085188187.1	*M. doricum*	WP_036464681.1	*M. neoaurum*	WP_011209110.1	*N. farcinica*
WP_098004285.1	*M. duvalii*	WP_083146226.1	*M. parafortuitum*	WP_143983179.1	*N. otitidiscaviarum*
WP_064893231.1	*M. elephantis*	EID09465.1	*M. phlei*	WP_067998050.1	*N. pseudobrasiliensis*
WP_061263084.1	*M. fortuitum*	WP_041303346.1	*M. rhodesiae*	WP_011597009.1	*R. jostii*
WP_085199921.1	*M. fragae*	KGI70340.1	*M. rufum*	WP_037669326.1	*S. griseus*
WP_025737208.1	*M. genavense*	WP_083410380.1	*M. rutilum*		
WP_011891998.1	*M. gilvum*	WP_044513983.1	*M. septicum*		

REFERENCES

1. Davis BG, Fairbanks AJ. *Carbohydrate Chemistry*. OUP Higher Education Division (2002).

2. Liao W, Lu D. Synthesis of a hexasaccharide acceptor corresponding to the reducing terminus of mycobacterial 3-*O*-methylmannose polysaccharide (MMP). *Carbohydr. Res.* **296**, 171-182 (1996).

3. Chen L, Kong F. A practical synthesis of α-d-Manp-(1→3)-α-d-Manp-(1→2)-[α-d-Glcp-(1→3)]-α-d-Manp-(1→2)-α-d-Manp-(1→2)-α-d-Manp, an O-specific heterohexasaccharide fragment of *Citrobacter braakii* O7a, 3b, 1c. *Carbohydr. Res.* **338**, 2169-2175 (2003).

4. Poláková M, Roslund MU, Ekholm FS, Saloranta T, Leino R. Synthesis of β-(1→2)-Linked Oligomannosides. *Eur. J. Org. Chem.* **2009**, 870-888 (2009).

5. Ripoll-Rozada J*, et al.* Biosynthesis of mycobacterial methylmannose polysaccharides requires a unique 1-*O*-methyltransferase specific for 3-*O*-methylated mannosides. *Proc. Natl. Acad. Sci. U. S. A.* **116**, 835-844 (2019).

6. Ankenbrand MJ, Keller A, Chain F. bcgTree: automatized phylogenetic tree building from bacterial core genomes. *Genome* **59**, 783-791 (2016).

7. Weisman LS, Ballou CE. Biosynthesis of the mycobacterial methylmannose polysaccharide. Identification of an alpha 1-->4-mannosyltransferase. *J. Biol. Chem.* **259**, 3457-3463 (1984).

8. Tian X, Li A, Farrugia IV, Mo X, Crich D, Groves MJ. Isolation and identification of poly-α-(1→4)-linked 3-*O*-methyl-D-mannopyranose from a hot-water extract of *Mycobacterium vaccae*. *Carbohydr. Res.* **324**, 38-44 (2000).

9. Stadthagen G*, et al.* Genetic basis for the biosynthesis of methylglucose lipopolysaccharides in *Mycobacterium tuberculosis*. *J. Biol. Chem.* **282**, 27270-27276 (2007).

10. Maitra SK, Ballou CE. Heterogeneity and refined structtures of 3-*O*-methyl-D-mannose polysaccharides from *Mycobacterium smegmatis*. *J. Biol. Chem.* **252**, 2459-2469 (1977).

11. Yamada H, Cohen RE, Ballou CE. Characterization of 3-*O*-methyl-D-mannose polysaccharide precursors in *Mycobacterium smegmatis*. *J. Biol. Chem.* **254**, 1972-1979 (1979).

12. Domon B, Costello CE. A systematic nomenclature for carbohydrate fragmentations in FAB-MS/MS spectra of glycoconjugates. *Glycoconj. J.* **5**, 397-409 (1988).

13. Neelamegham S*, et al.* Updates to the Symbol Nomenclature for Glycans guidelines. *Glycobiology* **29**, 620-624 (2019).

14. Saitou N, Nei M. The neighbor-joining method: a new method for reconstructing phylogenetic trees. *Mol. Biol. Evol.* **4**, 406-425 (1987).

15. Zuckerkandl E, Pauling L. Evolutionary Divergence and Convergence in Proteins. In: *Evolving Genes and Proteins* (eds Bryson V, Vogel HJ). Academic Press (1965).

16. Kumar S, Stecher G, Li M, Knyaz C, Tamura K. MEGA X: Molecular Evolutionary Genetics Analysis across Computing Platforms. *Mol. Biol. Evol.* **35**, 1547-1549 (2018).

17. Uotsu-Tomita R, Tonozuka T, Sakai H, Sakano Y. Novel glucoamylase-type enzymes from *Thermoactinomyces vulgaris* and *Methanococcus jannaschii* whose genes are found in the flanking region of the α-amylase genes. *Appl. Microbiol. Biotechnol.* **56**, 465-473 (2001).

18. Blum M*, et al.* The InterPro protein families and domains database: 20 years on. *Nucleic Acids Res.* **49**, D344-D354 (2021).

19. Bond CS, Schuttelkopf AW. ALINE: a WYSIWYG protein-sequence alignment editor for publication-quality alignments. *Acta Crystallogr. D Biol. Crystallogr.* **65**, 510-512 (2009).

20. Moreira ASP, Coimbra MA, Nunes FM, Simões J, Domingues MRM. Evaluation of the Effect of Roasting on the Structure of Coffee Galactomannans Using Model Oligosaccharides. *J. Agric. Food Chem.* **59**, 10078-10087 (2011).

21. Madeira F*, et al.* The EMBL-EBI search and sequence analysis tools APIs in 2019. *Nucleic Acids Res.* **47**, W636-W641 (2019).

22. Waterhouse AM, Procter JB, Martin DMA, Clamp M, Barton GJ. Jalview Version 2—a multiple sequence alignment editor and analysis workbench. *Bioinformatics* **25**, 1189-1191 (2009).

23. Yoon SH, Ha SM, Lim J, Kwon S, Chun J. A large-scale evaluation of algorithms to calculate average nucleotide identity. *Antonie Van Leeuwenhoek* **110**, 1281-1286 (2017).

24. Meier-Kolthoff JP, Auch AF, Klenk H-P, Göker M. Genome sequence-based species delimitation with confidence intervals and improved distance functions. *BMC Bioinformatics* **14**, 60 (2013).

25. Tortoli E*, et al.* Genome-based taxonomic revision detects a number of synonymous taxa in the genus *Mycobacterium*. *Infect. Genet. Evol.* **75**, 103983 (2019).

26. Pereira SG*, et al.* Studies of antimicrobial resistance in rare mycobacteria from a nosocomial environment. *BMC Microbiol.* **19**, 62 (2019).

27. Tiago I, Alarico S, Maranha A, Coelho C, Pereira SG, Empadinhas N. High-Quality Draft Genome Sequences of Rare Nontuberculous Mycobacteria Isolated from Surfaces of a Hospital. *Microbiol. Resour. Announc.* **8**, e00496-00419 (2019).

28. Adekambi T. *Mycobacterium mucogenicum* group infections: a review. *Clin. Microbiol. Infect.* **15**, 911-918 (2009).

29. Tortoli E. Microbiological Features and Clinical Relevance of New Species of the Genus *Mycobacterium*. *Clin. Microbiol. Rev.* **27**, 727-752 (2014).
