# Supplementary material for: Self-recycling and partially conservative replication of mycobacterial methylmannose polysaccharides
Source: Commun Biol. 2023 Jan 27;6:108. doi: 10.1038/s42003-023-04448-3 (PMC9883506; doi:10.1038/s42003-023-04448-3)
Supplement: Supplementary file 3 — Description of Additional Supplementary Files [file 42003_2023_4448_MOESM3_ESM.pdf]

# Description of Additional Supplementary Files

**File name:** Supplementary Data 1
**Description:** Source data for all plots in Excel format.
